# A unified analysis of global riverine eDNA reveals common associations of fish biodiversity with drainage characteristics

**DOI:** 10.1038/s41559-026-03106-1

**Published:** 2026-06-24

**Authors:** Yan Zhang, Heng Zhang, Hiroshi Akashi, Camille P. Albouy, Kara J. Andres, José Barquín, Jeanine Brantschen, Richard E. Connon, Joseph M. Craine, Deirdre Gleeson, Alejandra Goldenberg-Vilar, Alexia M. González-Ferreras, Chelsea Hatzenbuhler, Kamil Hupało, Josephine Hyde, Wataru Iwasaki, Mark D. Johnson, Aron D. Katz, Vyacheslav V. Kuzovlev, Courtney E. Larson, Laurène A. Lecaudey, Florian Leese, Matthieu Leray, Feilong Li, Till-Hendrik Macher, Quentin Mauvisseau, María Morán-Luis, Georgia Nester, Helio Quintero, Tsilavina Ravelomanana, Merin Reji Chacko, Mattia Saccò, Naiara Sales, Tamara Schenekar, Martin Schletterer, Saskia Schmidt, Nicholas O. Schulte, Robin Schütz, Jinelle H. Sperry, Emma R. Stevens, Sarah A. Stinson, Steven Weiss, Fei Xia, Hui Zhang, Song Zhang, Wenjun Zhong, Shuo Zong, Loïc Pellissier, Xiaowei Zhang, Florian Altermatt

**Affiliations:** 1 State Key Laboratory of Water Pollution Control and Green Resource Recycling, School of the Environment, Nanjing University, Nanjing, China; 2 Department of Aquatic Ecology, Eawag, Swiss Federal Institute of Aquatic Science and Technology, Dübendorf, Switzerland; 3 Department of Evolutionary Biology and Environmental Studies, Universität Zürich, Zurich, Switzerland; 4 Department of Natural Sciences, Faculty of Arts and Sciences, Komazawa University, Setagaya, Japan; 5 Ecosystems and Landscape Evolution, Department of Environmental Systems Science, Institute of Terrestrial Ecosystems, ETH Zürich, Zurich, Switzerland; 6 Unit of Land Change Science, Swiss Federal Research Institute for Forest, Snow and Landscape Research (WSL), Birmensdorf, Switzerland; 7 Department of Ecology and Evolutionary Biology, Cornell University, Ithaca, NY USA; 8 School of Biological Sciences, Illinois State University, Normal, IL USA; 9 IHCantabria—Instituto de Hidráulica Ambiental de la Universidad de Cantabria, Santander, Spain; 10 Center for Ocean Solution, Hopkins Marine Station, Monterey, CA USA; 11 School of Veterinary Medicine, University of California, Davis, CA USA; 12 Jonah Ventures, Boulder, CO USA; 13 UWA School of Agriculture and Environment, The University of Western Australia, Perth, Western Australia Australia; 14 SpecPro Sustainment and Environment, Office of Research and Development, US Environmental Protection Agency, Duluth, MN USA; 15 Aquatic Ecosystem Research, Faculty of Biology, University of Duisburg-Essen, Essen, Germany; 16 Aquatic Ecology, Faculty of Biology, University of Duisburg-Essen, Essen, Germany; 17 Biodiversity and Conservation Science, Department of Biodiversity, Conservation and Attractions, Government of Western Australia, Kensington, Western Australia Australia; 18 Biologic Environmental Survey, East Perth, Western Australia Australia; 19 Department of Integrated Biosciences, Graduate School of Frontier Sciences, The University of Tokyo, Kashiwa, Japan; 20 Engineering Research and Development Center, Champaign, IL USA; 21 Department of Entomology, University of Illinois at Urbana-Champaign, Urbana, IL USA; 22 Faculty of Environmental Management and Ecology, Tver State Technical University, Tver, Russia; 23 Tver Center for Hydrometeorology and Environmental Monitoring, Russian Federal Service for Hydrometeorology and Environmental Monitoring, Moscow, Russia; 24 Office of Research and Development, US Environmental Protection Agency, Duluth, MN USA; 25 SINTEF Ocean, Aquaculture Department, Trondheim, Norway; 26 Smithsonian Tropical Research Institute, Panama City, Panama; 27 School of Biological Sciences, Swire Institute of Marine Science, The University of Hong Kong, Hong Kong, China; 28 Guangdong Basic Research Center of Excellence for Ecological Security and Green Development, Guangdong Provincial Key Laboratory of Water Quality Improvement and Ecological Restoration for Watersheds, School of Ecology, Environment and Resources, Guangdong University of Technology, Guangzhou, China; 29 University of Trier, Biogeography, Trier, Germany; 30 Natural History Museum, University of Oslo, Oslo, Norway; 31 Trace and Environmental DNA (TrEnD) Lab, School of Molecular and Life Sciences, Curtin University, Perth, Western Australia Australia; 32 Minderoo OceanOmics Centre at UWA, Oceans Institute, University of Western Australia, Crawley, Western Australia Australia; 33 Biology of Aquatic Population Laboratory, Antananarivo University, Antananarivo, Madagascar; 34 Laboratoire de Biologie des Organismes et des Écosystèmes Aquatiques (BOREA), Muséum national d’Histoire naturelle, Sorbonne Université, CNRS, IRD, Paris, France; 35 School of Science, Engineering and Environment, University of Salford, Salford, UK; 36 Department of Biology, University of Graz, Graz, Austria; 37 BOKU University, Department of Ecosystem Management, Climate and Biodiversity, Institute of Hydrobiology and Aquatic Ecosystem Management, Vienna, Austria; 38 Animal Ecology, Federal Institute of Hydrology, Koblenz, Germany; 39 CAS Key Laboratory of Marine Ecology and Environmental Sciences, Institute of Oceanology, Chinese Academy of Sciences, Qingdao, China; 40 State Key Laboratory of Vegetation Structure, Function and Construction (VegLab) & Yunnan Key Laboratory for Plateau Mountain Ecology and Restoration of Degraded Environments, School of Ecology and Environmental Sciences, Yunnan University, Kunming, China

**Keywords:** Biodiversity, Environmental impact

## Abstract

Freshwater biodiversity is declining at a pace that outstrips the capacity of existing monitoring approaches both in temporal and spatial dimensions, highlighting the urgent need for rapid and scalable assessment and attribution of biodiversity states and changes. Here we present a global assessment and unified analysis of riverine fish biodiversity using environmental DNA collected from 1,818 sites across 113 river systems spanning 5 continents. We quantified species richness, functional redundancy, phylogenetic diversity and genetic sequence diversity, and related them to drainage characteristics. Our results showed that environmental DNA effectively captured global patterns of multi-faceted riverine fish biodiversity and disentangled the roles of climate and different aspects of human activities in shaping biodiversity–area relationships. The accumulation of biodiversity with catchment area was consistently enhanced in warmer climates, while human activities weakened this scaling. Further, species richness, functional and genetic sequence diversity exhibited stronger negative responses to human activities in larger catchments. In contrast, phylogenetic diversity was negatively affected by human activities, yet this effect diminished as catchment area increased, highlighting the facet-dependent nature of biodiversity responses to environmental gradients and catchment characteristics. Our findings demonstrate the power of environmental-DNA-based datasets for harmonized, multi-faceted biodiversity assessments, offering a scalable approach for detecting and attributing biodiversity change and informing conservation strategies under accelerating global change.

## Abstract (web summary)

Using environmental DNA from 113 river systems, this study presents a unified analysis of global riverine fish biodiversity to examine how effectively environmental DNA captures multiple facets of biodiversity patterns and how fish biodiversity responds to variation in drainage properties and human activities.

## Main

Global biodiversity is declining at an unprecedented rate, while efforts in biodiversity conservation and ecosystem restoration are lagging behind^1–3^. Next to declines in richness and population sizes, the erosion of within-population genetic diversity is becoming increasingly apparent, with losses occurring worldwide under the influence of human activities^4,5^. Freshwater ecosystems cover less than 1% of Earth’s surface but sustain nearly 10% of known species, underscoring their disproportionate importance to global biodiversity^6^. Yet, freshwater ecosystems are also among the most threatened biomes, with population declines of up to 85% for freshwater species^4,7,8^. These declines are particularly strong in freshwater fishes, which are both highly vulnerable^9,10^, yet also particularly important with respect to ecosystem services. The declines are jointly driven by multiple pressures including climate change, land-use transformation, overexploitation and biological invasions, which have profoundly reshaped species distributions, community structures and ecosystem functions^11^.

For effective conservation and mitigation of these challenges, there is not only a need to understand the drivers themselves, but also to understand their effects in the context of universal physical structures and properties of river networks^12^, which shape the distribution and persistence of riverine biodiversity. River networks exhibit hierarchical branching structures that constrain the movement of organisms, resources and energy^12–14^. This dendritic configuration embeds river systems within the landscape, where upstream flows transport biotic and abiotic signals that shape downstream ecosystems^15,16^. Biodiversity is generally expected to increase from headwaters to mainstems due to stronger connectivity and cumulative species inflow, following a typical biodiversity–area relationship^17^. However, these patterns are not static. Climatic variation and human activities interact with hydrological processes, altering biodiversity patterns by reshaping community structure and diversity patterns along the river continuum^18–20^. Such spatial heterogeneity in responses to human activities may partly explain why variation in biodiversity patterns has been observed across individual catchments worldwide^21,22^. Variable patterns may also partially reflect the complex interplay between river network structure, climatic gradients and human activities. Yet, past work on riverine diversity is often based on very different monitoring and assessment approaches, and the universality of local to global riverine diversity patterns is subject for debate^23,24^.

Traditionally, biodiversity has been looked at from a species richness perspective. However, variation in functional, phylogenetic and genetic diversity is further dimensions of diversity capturing complementary aspects of ecosystem structure, function and stability^10,25^. Functional diversity reflects ecological niche differentiation and functional robustness, with functional redundancy enhancing ecosystem resilience to species loss^26^. Phylogenetic diversity describes the evolutionary distinctiveness of the community^27^. Finally, genetic diversity is often associated with the capacity of populations to respond to environmental change, which can reduce population fluctuations and extinction risk, ultimately enhancing the long-term functional stability of ecosystems^5,28^. Despite the growing emphasis on monitoring biodiversity across multiple facets, as, for example, advocated by the Global Biodiversity Framework^29^, comprehensive assessments of the spatial patterns and drivers of multi-faceted biodiversity in riverine ecosystems are largely lacking.

In recent years, environmental DNA (eDNA) has emerged as a transformative tool for aquatic biodiversity monitoring and has been widely applied across ecosystems^30^. Environmental DNA approaches capture taxonomic diversity^31,32^ and enable the estimation of functional diversity and link to species’ trait data^33,34^, the calculation of phylogenetic diversity and, to some extent, the assessment of genetic diversity from sequence variation in commonly used metabarcoding markers, which can serve as a first proxy for broader standing genetic variation^35,36^. As such, it has become the method of choice for the rapid and quantitative assessment of multiple facets of biodiversity at larger spatial scales^37,38^, possibly enabling scalable and spatially representative biodiversity monitoring and observation networks^39^. While the effectiveness of eDNA has been well validated at regional scales, most existing applications remain focused on specific regions or individual facets of biodiversity^16,40,41^. Recently, the use of aquatic eDNA to address global biodiversity targets has been postulated^42^, yet it requires a demonstration of consistent and robust assessment of multidimensional biodiversity patterns across catchment basins, climate zones and gradients of human activities.

**Fig. 1:**
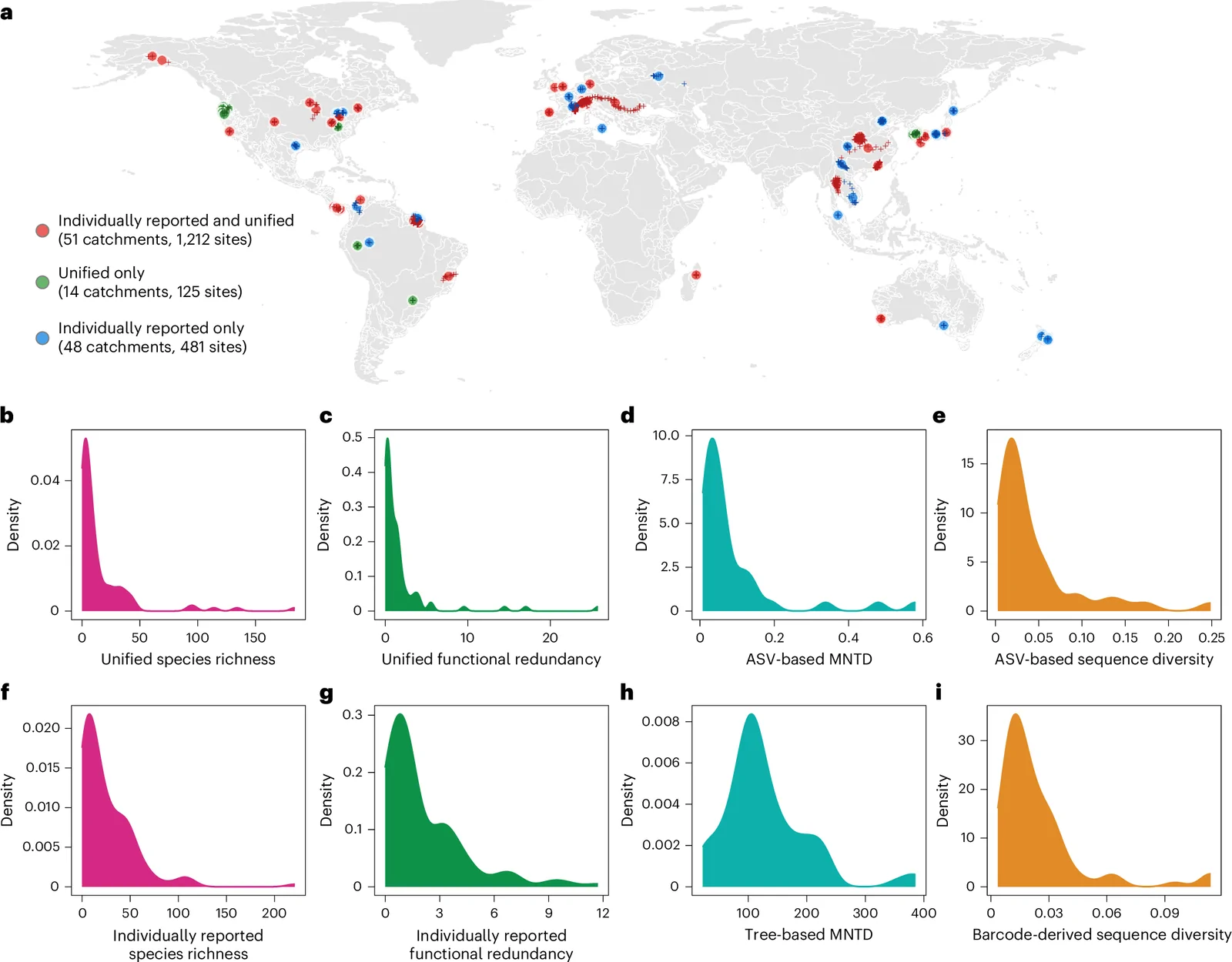
Global synthesis of fish diversity based on eDNA data. **a**, Geographic distribution of 113 river catchments (circles), comprising a total of 1,818 eDNA sampling sites included in this meta-analysis (sites are added as crosses). Fish community data were compiled from studies that either comprised original individually reported fish diversity metrics (blue), provided raw sequencing data for unified and standardized analysis (green) or both (red). **b**–**e**, The unified fish diversity facets derived from a standardized bioinformatic pipeline applied to raw eDNA sequencing data, including species richness (**b**), functional redundancy calculated from unified species-by-site tables using species-level trait information (**c**), MNTD calculated from ASVs (**d**) and genetic sequence diversity estimated from within-species nucleotide variation based on ASV sequences (**e**). **f**,**g**, Original individually reported diversity metrics based on values from individual studies, including species richness (**f**) and, when available, functional redundancy (**g**). **h**, MNTD computed from unified species-by-site tables using a published global fish phylogeny to quantify phylogenetic relatedness among species. **i**, Barcode-derived genetic sequence diversity calculated from unified species lists by retrieving COI sequences for each species from NCBI and computing within-species nucleotide diversity. Source data

Here we analyse multi-faceted riverine fish biodiversity patterns, covering species, functional, phylogenetic and genetic diversity, in a unifying framework and link these diversity patterns to natural and human-related drivers. Using a newly compiled, globally unified and harmonized fish eDNA dataset^43^ (Fig. 1 and Supplementary Table 1), which integrates both original individually reported and unified community data from 113 river catchments across six continents and encompasses 1,818 individual sampling sites, we address three key questions: (1) to what extent does eDNA capture globally relevant, macroecological patterns of riverine fish biodiversity (covering multiple facets including taxonomic, functional, phylogenetic and genetic sequence diversity), and how consistent are these patterns when compared to conventional biodiversity data? (2) How do drainage characteristics, particularly climatic and human activities in combination with catchment area, modulate biodiversity–area relationships across river systems? (3) How sensitive is fish biodiversity to human activities across the river network, and are there generalizable differences between upstream and downstream locations or across drainage sizes? By combining biodiversity metrics derived from eDNA with drainage characteristics, our study provides a global-scale test of eDNA-based riverine biodiversity assessments, demonstrating how fish biodiversity responds to variation in drainage properties and human activities.

## Results

### Validation of eDNA-derived biodiversity

We characterized riverine fish diversity across four complementary biodiversity facets—species richness, functional redundancy, mean nearest taxon distance (MNTD) and genetic sequence diversity—based on a unified and standardized analysis of raw eDNA sequencing data (Fig. 1). For comparison, we also calculated four corresponding metrics: species richness and functional redundancy from original individually reported eDNA datasets, phylogenetic diversity represented as tree-based MNTD from a global fish phylogeny and barcode-derived genetic sequence diversity derived from cytochrome *c* oxidase subunit I (COI) barcode sequences in the National Center for Biotechnology Information (NCBI) database. Because the compiled fish eDNA datasets differed in primer choice and sampling volume, we evaluated the robustness of downstream inferences by including primer or barcode identity and sampling volume as terms in the statistical models, as described in Methods.

Across facets, correlation analyses among diversity facets revealed that species richness and functional redundancy were strongly correlated, whereas phylogenetic and genetic sequence diversity showed weaker associations with other facets (Supplementary Fig. 1). Species richness and functional redundancy both exhibited clear tropical peaks and declined towards higher latitudes (generalized additive model: *R*^2^ = 0.18 and 0.16, respectively; *P* < 0.001; Supplementary Fig. 2). Phylogenetic diversity, in contrast, was higher at higher latitudes, particularly for tree-based MNTD, which showed a stronger positive relationship with latitude (*R*^2^ = 0.14, *P* < 0.001). Genetic sequence diversity was generally highest in the tropics, especially when derived from COI barcodes (*R*^2^ = 0.15, *P* < 0.001) with weaker signals for amplicon sequence variant (ASV)-based estimates (*R*^2^ = 0.08). Together, these results reveal marked spatial heterogeneity across taxonomic, functional, phylogenetic and genetic dimensions of riverine fish diversity at the global scale.

**Fig. 2:**
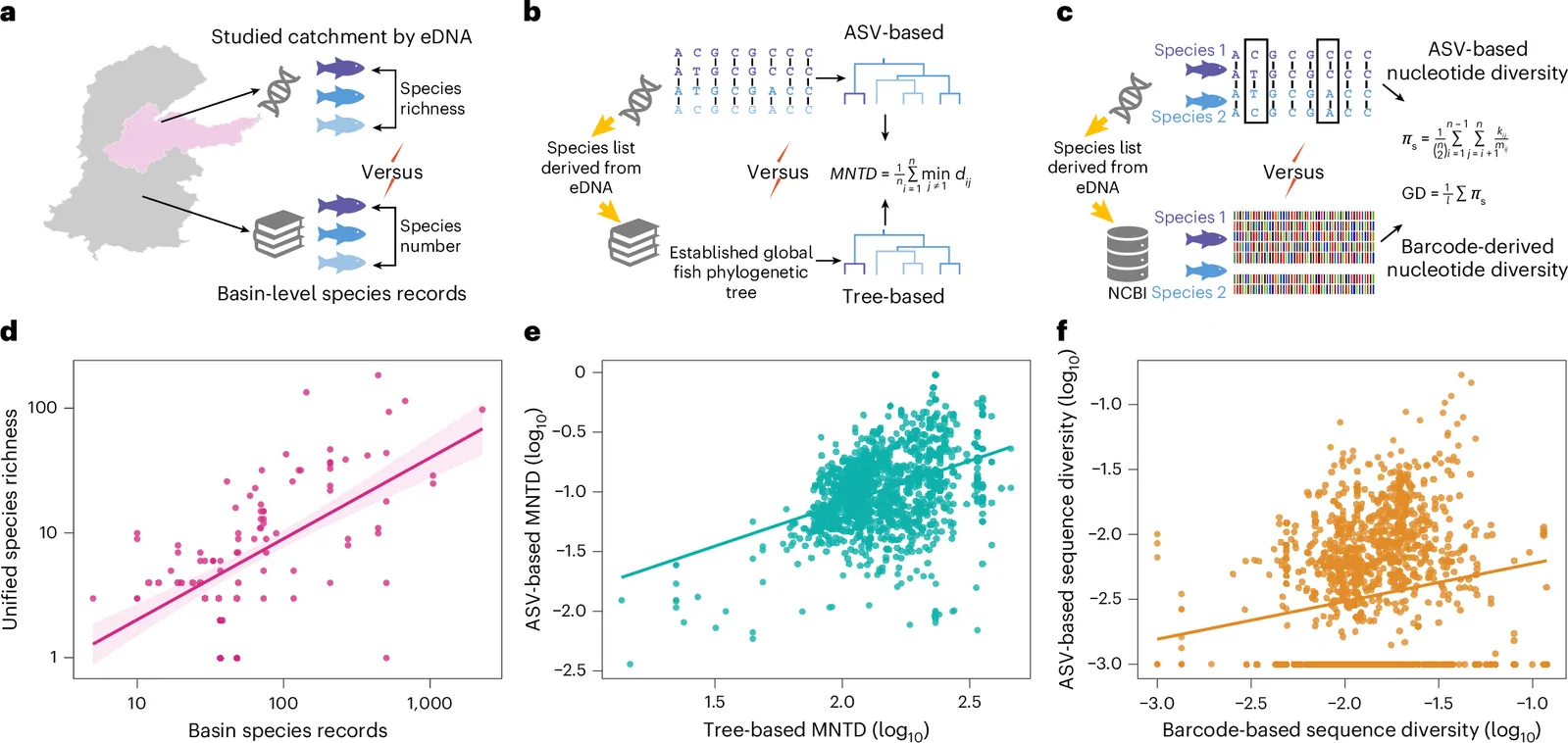
Assessing the ability of eDNA-based data to capture global patterns of multi-faceted fish biodiversity. **a**–**c**, Conceptual diagrams illustrating the validation framework for three biodiversity facets, including taxonomic (**a**), phylogenetic (**b**) and genetic (**c**) diversity. The lightning ‘Versus’ indicates a comparison between eDNA-derived and reference-based biodiversity estimates. **d**, Relationship between eDNA-derived species richness (unified datasets) at the river catchment scale and recorded species numbers from historical data at the corresponding river basin scale. **e**, Comparison of ASV-based MNTD with tree-based MNTD derived from a global freshwater fish phylogeny. **f**, Comparison of genetic sequence diversity estimated from within-species sequence variation with barcode-based genetic sequence diversity derived from COI sequences in NCBI. All relationships were modelled using GLMMs, and significance was assessed using two-sided Wald tests. Shaded areas indicate 95% confidence intervals. The exact *P* values, standard errors and model coefficients are reported in Extended Data Table 1. Source data

**Extended Data Fig. 1:**
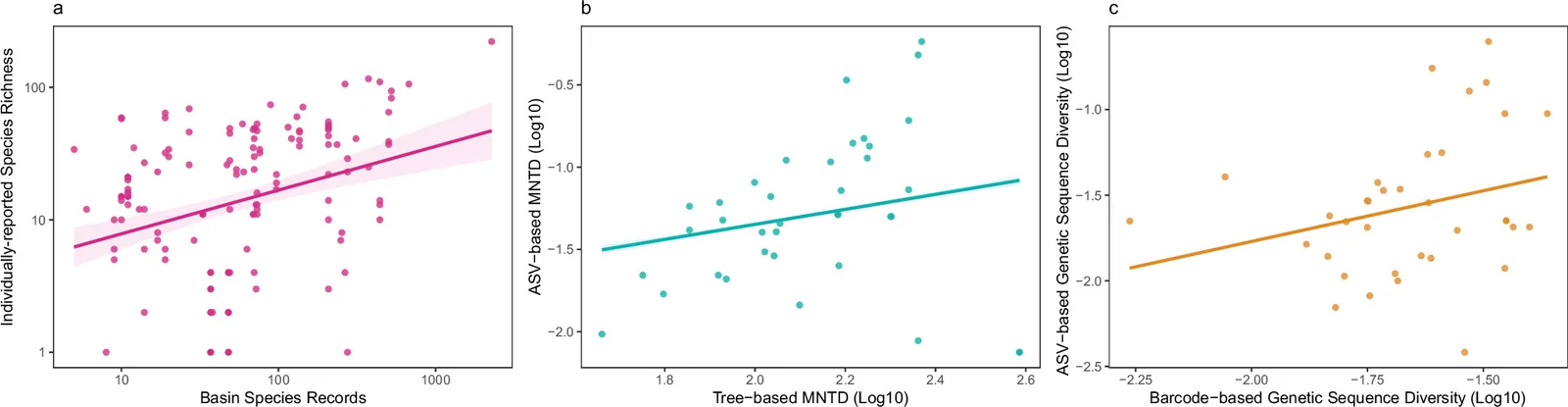
Validation of gamma biodiversity metrics. **a**, Relationship between original individually-reported species richness for each river catchment and original recorded species numbers from historical data at the corresponding river basin scale. **b**, Comparison of ASV-based MNTD with tree-based MNTD derived from a global freshwater fish phylogeny. **c**, Comparison of genetic sequence diversity estimated from within-species sequence variation with barcode-based genetic sequence diversity derived from COI sequences in NCBI. All relationships were modelled using generalized linear mixed models, and significance was assessed using two-sided Wald tests. Shaded areas indicate 95% confidence intervals. The exact *p* values, standard errors and model coefficients are reported in Extended Data Table 1. Source data

**Extended Data Fig. 2:**
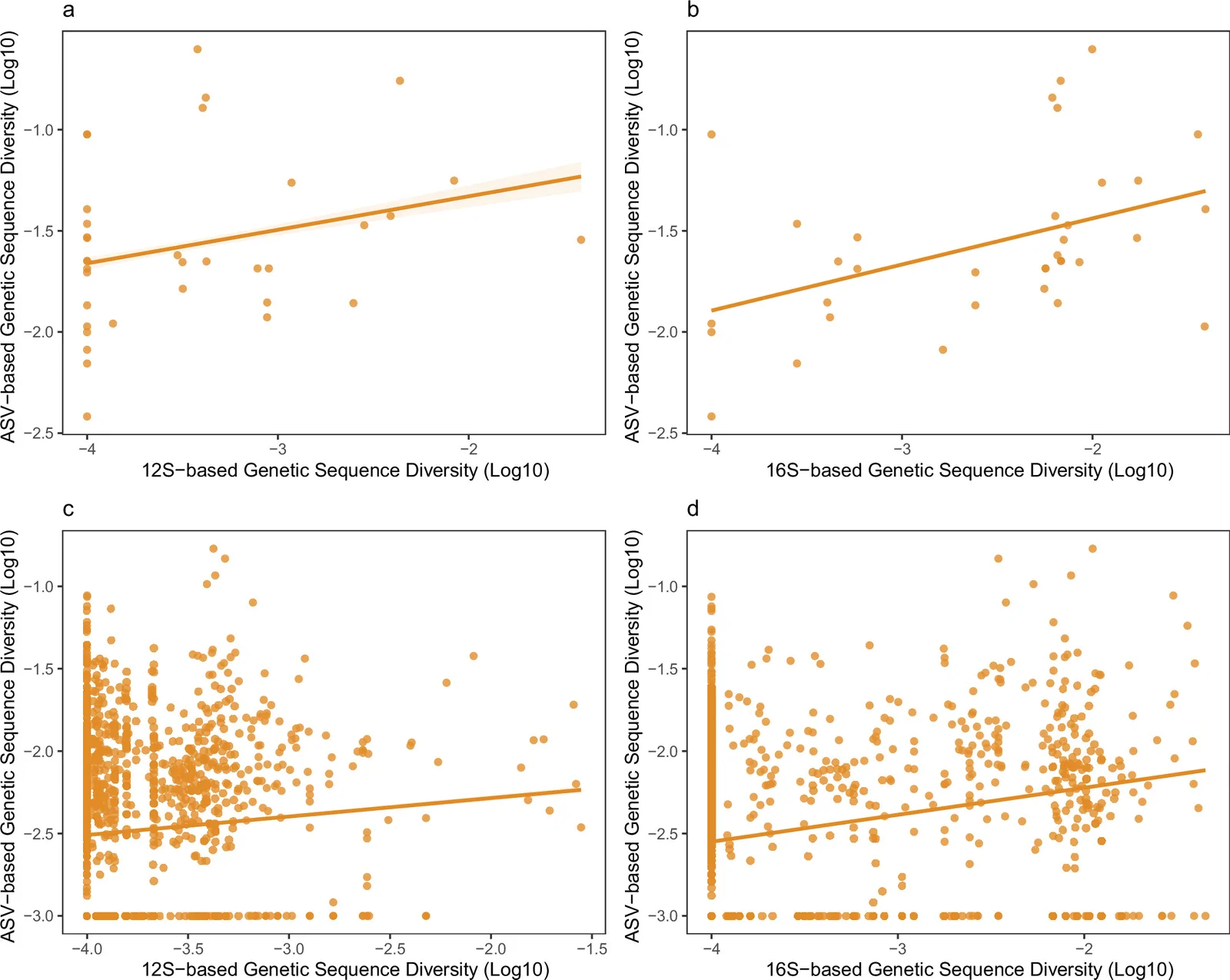
Comparison of genetic sequence diversity estimated from within-species ASV sequence variation and barcode-based genetic sequence diversity. **a,b**, Comparison of gamma-level genetic sequence diversity derived from ASV sequences with barcode-based genetic sequence diversity calculated from 12S (**a**) and 16S (**b**) marker sequences. **c, d**, Comparison of alpha-level genetic sequence diversity based on ASV sequences with corresponding barcode-based estimates from 12S (**c**) and 16S (**d**) markers. All relationships were evaluated using generalized linear mixed models, and significance was assessed using two-sided Wald tests. Shaded areas represent 95% confidence intervals. The exact *p* values, standard errors and model coefficients are reported in Extended Data Table 1. Source data

To evaluate the reliability of eDNA-derived diversity metrics, we systematically compared them with conventional biodiversity records (Fig. 2 and Extended Data Table 1). Unified species richness was highly correlated with historical basin-level species records, showing robust consistency in models that included either primer (generalized linear mixed model (GLMM), *P* < 0.001, marginal *R*^2^ = 0.16, conditional *R*^2^ = 0.70) or barcode (*P* < 0.001, marginal *R*^2^ = 0.27, conditional *R*^2^ = 0.51) as a random effect. Similarly, the correlations were also significant for the original individually reported species richness (GLMM, *P* < 0.001, marginal *R*^2^ = 0.08–0.10, conditional *R*^2^ = 0.39–0.68; Extended Data Fig. 1). For phylogenetic diversity, alpha diversity estimated from ASV-based MNTD showed a strong correlation with that from tree-based MNTD (*P* < 0.001, marginal *R*^2^ = 0.22, conditional *R*^2^ = 0.62; in models that included barcode as a random effect, *P* < 0.001, marginal *R*^2^ = 0.19, conditional *R*^2^ = 0.22), whereas gamma diversity showed notably weaker correlation (*P* > 0.05, marginal *R*^2^ = 0.04–0.05, conditional *R*^2^ = 0.05–0.20; Extended Data Fig. 1). For genetic sequence diversity, gamma diversity of ASV-based within-species nucleotide diversity was marginally significantly correlated with barcode-derived estimates (*P* = 0.094, marginal *R*^2^ = 0.08, conditional *R*^2^ = 0.08; Extended Data Fig. 1), while alpha diversity showed a weaker but highly significant correlation in models that included barcode as a random effect (*P* < 0.001, marginal *R*^2^ = 0.02, conditional *R*^2^ = 0.19). This pattern was consistent across genetic sequence diversity estimates derived from both 12S and 16S barcode sequences (Extended Data Fig. 2), demonstrating the robustness of ASV-based genetic sequence diversity relative to traditional barcode-based approaches.

### Drivers of multi-faceted biodiversity

**Fig. 3:**
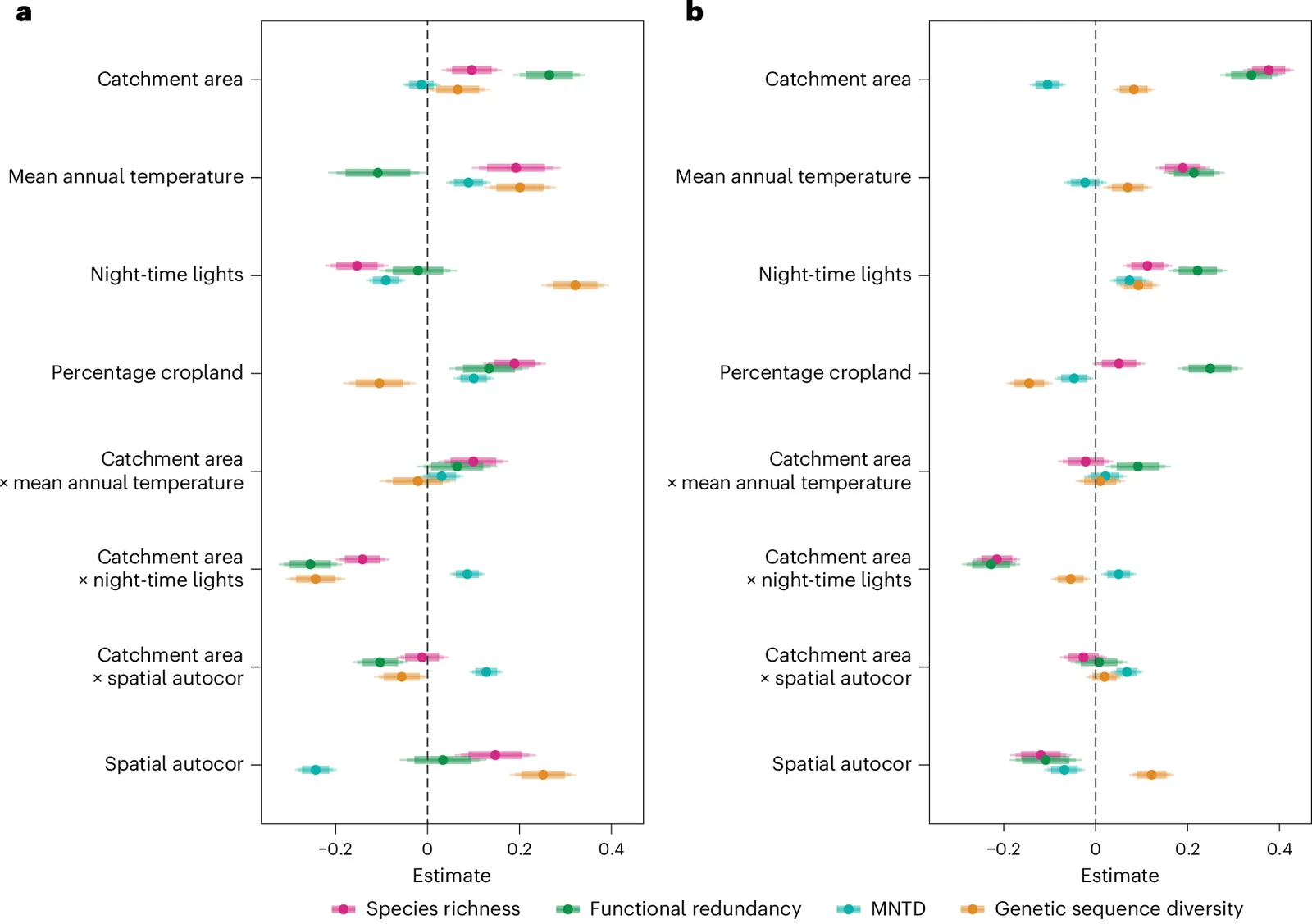
Standardized effect sizes of drainage characteristics on multi-faceted fish biodiversity. Drainage characteristics (including catchment area and its interactions with environmental variables) were modelled across four biodiversity facets (species richness, functional redundancy, MNTD and genetic sequence diversity). Estimates were obtained using GLMMs. **a**, Models based on all unified eDNA datasets (species richness: 1,244 sites; functional redundancy: 1,111 sites; MNTD: 1,067 sites; genetic sequence diversity: 1,199 sites). **b**, Models based on the original individually reported or conventional diversity metrics (species richness: 1,601 sites; functional redundancy: 1,434 sites; MNTD: 1,023 sites; genetic sequence diversity: 1,116 sites). Points represent estimated fixed effects, with bar lengths indicating 80% (long), 90% (medium) and 95% (short) confidence intervals. The vertical dashed line indicates zero effect. Source data

Drainage characteristics, encompassing catchment area, climate and human activity intensity, shape biodiversity facets, yielding consistent yet facet-specific patterns (Fig. 3 and Supplementary Table 2). Both unified and individually reported species richness were consistently and positively associated with catchment area (estimate = 0.10, *P* = 0.004 for unified; estimate = 0.38, *P* < 0.001 for individually reported) and temperature (estimate = 0.19, *P* < 0.001 for unified; estimate = 0.19, *P* < 0.001 for individually reported). Night-time light intensity had different effects between data types, showing a negative relationship with unified species richness (estimate = −0.15, *P* < 0.001) but a positive relationship with individually reported species richness (estimate = 0.11, *P* < 0.001). Cropland cover was strongly and positively related to unified species richness (estimate = 0.19, *P* < 0.001), whereas its effect on individually reported species richness was weaker and not significant (estimate = 0.05, *P* = 0.083). Both models revealed a strong negative interaction between catchment area and night-time light density (estimate = −0.14 and −0.22, *P* < 0.001). Additionally, a positive interaction between catchment area and temperature was significant for unified species richness (estimate = 0.10, *P* = 0.010), but not for individually reported richness (estimate = −0.01, *P* = 0.477).

Functional redundancy exhibited a broadly similar pattern. It was strongly and positively associated with catchment area (estimate = 0.26 for unified and 0.34 for individually reported, *P* < 0.001; Fig. 3 and Supplementary Table 2), while the negative interaction with human activity was captured by the catchment area interaction with night-time light intensity (estimate = −0.26 and −0.23, both *P* < 0.001). Night-time light intensity showed no significant main effect for unified functional redundancy (*P* = 0.629) but a significant positive main effect for individually reported functional redundancy (estimate = 0.22, *P* < 0.001). Cropland cover showed a positive main effect on functional redundancy for both data types (estimate = 0.13, *P* = 0.002 for unified; estimate = 0.25, *P* < 0.001 for individually reported). Temperature effects were again divergent between datasets, being negative for unified data (estimate = −0.11, *P* = 0.049) but positive for the individually reported data (estimate = 0.21, *P* < 0.001). A positive interaction between catchment area and temperature was significant for both data types based on likelihood ratio tests (*χ*^2^ = 8.42, *P* = 0.004 for unified; *χ*^2^ = 18.52, *P* < 0.001 for individually reported), suggesting that the functional benefits of larger catchments were amplified under warmer conditions, despite the interaction term being less evident in the unified coefficient table (*P* = 0.147).

Phylogenetic diversity, measured by MNTD, was predominantly shaped by catchment-scale and environmental gradients (Fig. 3 and Supplementary Table 2). ASV-based MNTD increased with temperature (estimate = 0.09, *P* < 0.001) and cropland cover (estimate = 0.10, *P* < 0.001), but decreased with night-time light intensity (estimate = −0.09, *P* < 0.001). Catchment area showed no significant main effect on ASV-based MNTD (estimate = −0.01, *P* = 0.518), and the interaction between catchment area and temperature was not significant (estimate = 0.03, *P* = 0.218), whereas the interaction between catchment area and night-time light intensity was significantly positive (estimate = 0.09, *P* < 0.001), indicating that the relationship between catchment area and ASV-based MNTD became more positive under higher night-time light intensity. Tree-based MNTD exhibited a significant negative main effect for catchment area (estimate = −0.10, *P* < 0.001), a positive association with night-time light intensity (estimate = 0.07, *P* < 0.001) and a negative association with cropland cover (estimate = −0.05, *P* = 0.031). The interaction between catchment area and night-time light intensity remained significantly positive (estimate = 0.05, *P* = 0.010), suggesting that higher night-time light intensity attenuated the negative catchment area effect on tree-based MNTD. For tree-based MNTD, temperature was not significantly associated either as a main effect (estimate = −0.02, *P* = 0.343) or through its interaction with catchment area (estimate = 0.02, *P* = 0.367).

Genetic sequence diversity revealed broadly consistent directional responses but different magnitudes between ASV-based and barcode-derived metrics. ASV-based genetic sequence diversity increased with temperature (estimate = 0.20, *P* < 0.001; Fig. 3 and Supplementary Table 2) and night-time light density (estimate = 0.32, *P* < 0.001), while catchment area showed a marginal main effect (estimate = 0.07, *P* = 0.071). Cropland cover had a significant negative association with ASV-based sequence diversity (estimate = −0.10, *P* = 0.009). The negative interaction between catchment area and night-time light density was strongly significant (estimate = −0.24, *P* < 0.001), indicating that spatial scaling benefits were reduced under higher urbanization. For barcode-derived genetic sequence diversity, catchment area (estimate = 0.08, *P* < 0.001) and night-time light intensity (estimate = 0.09, *P* < 0.001) all had significant positive effects, while cropland cover was negatively associated (estimate = −0.14, *P* < 0.001). Temperature also showed a positive association (estimate = 0.07, *P* = 0.010). The interaction between catchment area and temperature was not significant for barcode-based genetic sequence diversity (estimate = 0.01, *P* = 0.719) or ASV-based diversity (estimate = −0.02, *P* = 0.617). The negative interaction between catchment area and night-time light intensity was consistently significant across both metrics (estimate = −0.24 for ASV-based, *P* < 0.001; estimate = −0.05 for barcode-based, *P* = 0.014).

### Climate and human activity modulation of biodiversity–area relationships

Across all biodiversity facets, the interaction terms between catchment area and both climate- and human-related drivers consistently emerged as dominant predictors of biodiversity–area relationships (Fig. 3 and Supplementary Table 3). First, the negative interaction between catchment area and night-time light density was pervasive except for MNTD, indicating reduced biodiversity–area scaling under high urbanization (*χ*^2^ = 5.69–71.56, *P* < 0.05). By contrast, the corresponding interaction with cropland cover was generally non-significant (*P* > 0.05), except for ASV-based sequence diversity (*P* = 0.025) and unified functional redundancy (*P* < 0.001). Second, and in contrast, the positive interaction between catchment area and temperature was consistently detected for species richness, functional redundancy and barcode-derived genetic sequence diversity, reflecting stronger biodiversity–area scaling in warmer regions (*χ*^2^ = 5.31–18.52, *P* < 0.05). These results indicate that biodiversity–area relationships might be jointly modulated by climatic and human activity gradients. Therefore, we presented how biodiversity–area relationships were modulated by temperature and night-time light density across facets.

**Fig. 4:**
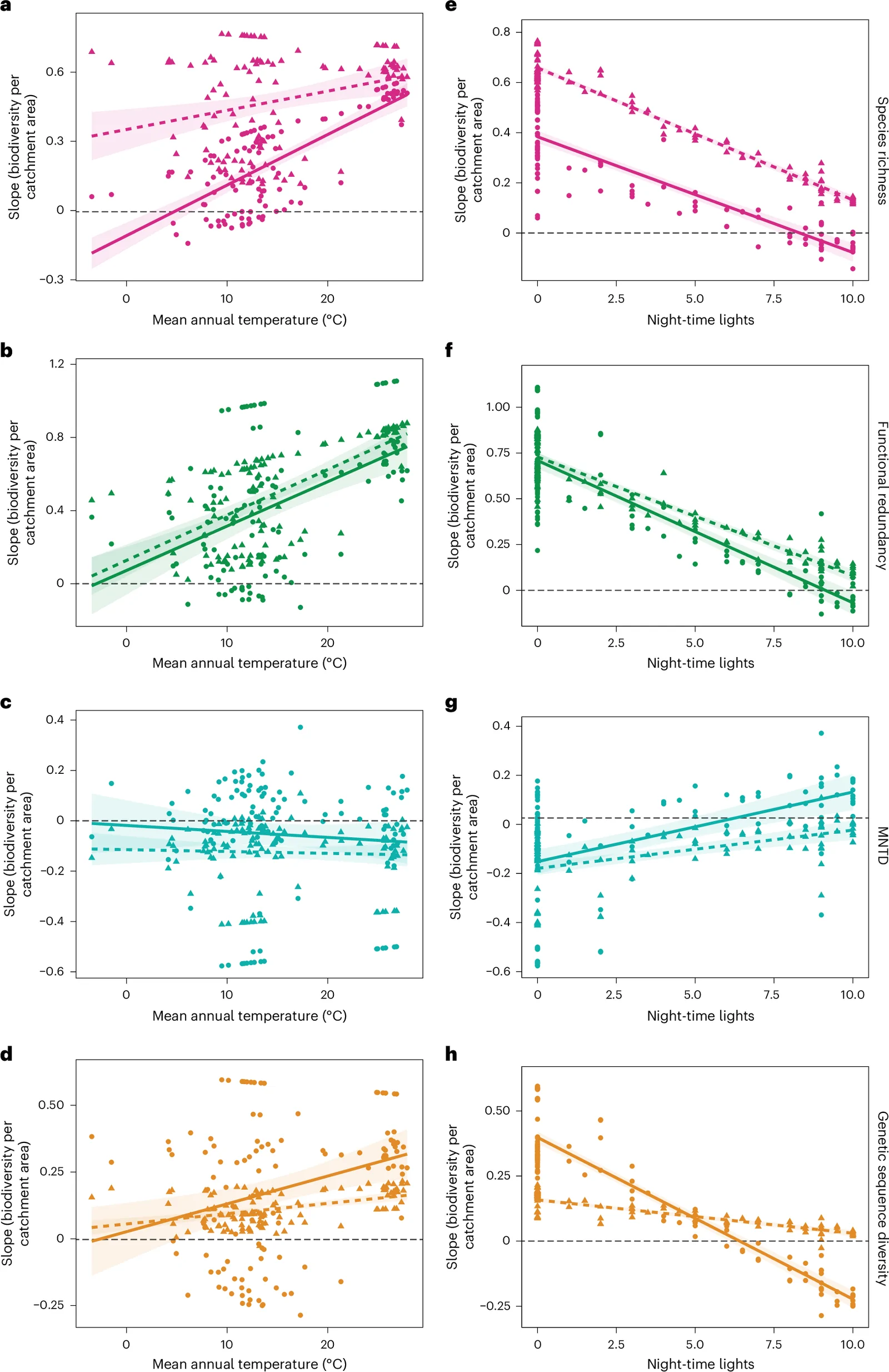
Effects of temperature and human activities on biodiversity–area relationships across river catchments. **a**–**d**, Relationships between diversity–area slopes for species richness (**a**), functional redundancy (**b**), MNTD (**c**) and genetic sequence diversity (**d**) estimated from GLMMs using the median of mean annual temperature for each river catchment. **e**–**h**, Relationships between diversity–area slopes for species richness (**e**), functional redundancy (**f**), MNTD (**g**) and genetic sequence diversity (**h**) estimated from GLMMs using night-time light density for each river catchment. Each point represents a river catchment. Coloured lines are generalized linear model fits for each biodiversity facet. Solid lines and circles represent unified biodiversity metrics, while dashed lines and triangles represent original individually reported or conventional metrics. Shaded areas indicate 95% confidence intervals. The exact *P* values, standard errors and model coefficients are reported in Extended Data Table 2. Source data

**Extended Data Fig. 3:**
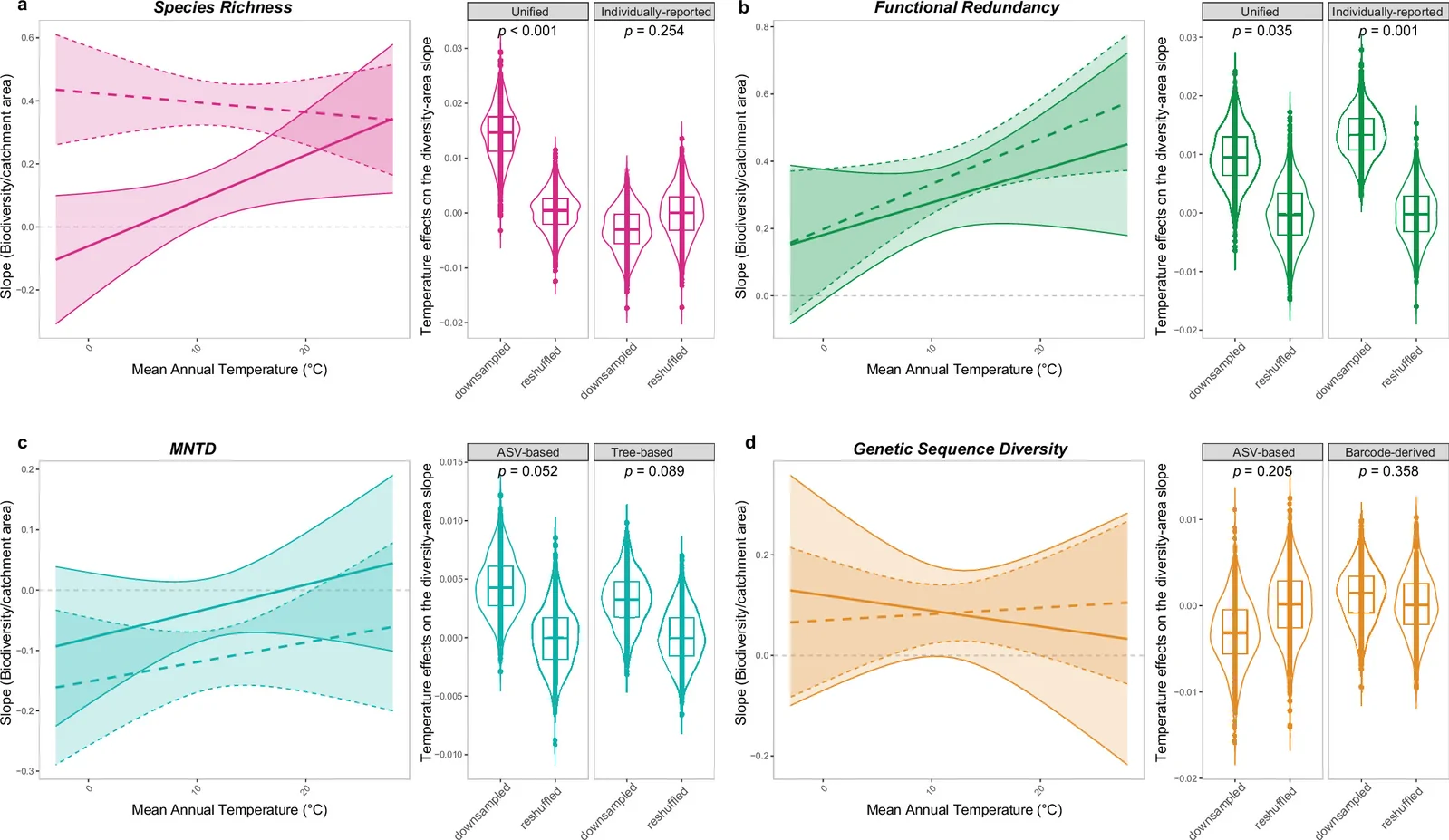
Relationships between temperature and the slope of biodiversity responses to catchment area across four biodiversity facets. Panels show species richness (**a**), functional redundancy (**b**), MNTD (**c**) and genetic sequence diversity (**d**). Slopes were estimated from 1,000 generalized linear mixed model fits using 70% downsampling. Solid lines represent Unified biodiversity metrics; dashed lines represent Original individually-reported or conventional metrics. Shaded areas show 95% confidence intervals, and *p* values reflect the proportion of reshuffled effects exceeding the median estimate. For *p* < 0.001, this indicates that none of the 1000 reshuffling iterations resulted in an estimated slope exceeding the median of the downsampling distribution. For box plots, the centre line indicates the median, the box bounds indicate the 25^th^ and 75^th^ percentiles, and the whiskers extend to the most extreme data points within 1.5 × the interquartile range. Source data

The relationship between biodiversity–area slopes and temperature exhibited consistent but facet-specific patterns (Fig. 4a–d and Extended Data Table 2). For species richness, functional redundancy and genetic sequence diversity, slopes increased significantly with temperature (unified species richness: *R*^2^ = 0.60, *P* < 0.001; individually reported species richness: *R*^2^ = 0.07, *P* = 0.002; unified functional redundancy: *R*^2^ = 0.25, *P* < 0.001; individually reported functional redundancy: *R*^2^ = 0.44, *P* < 0.001; ASV-based sequence diversity: *R*^2^ = 0.08, *P* = 0.002; barcode-derived sequence diversity *R*^2^ = 0.22, *P* < 0.001), indicating that warmer climates promote a higher rate of biodiversity accumulation with spatial expansion. MNTD displayed a distinct pattern, with neither tree-based nor ASV-based MNTD showing a significant relationship with temperature (ASV-based *R*^2^ < 0.01, *P* = 0.431; tree-based *R*^2^ < 0.001, *P* = 653). Resampling provided partial support for these temperature-driven modulations (Extended Data Fig. 3). In particular, the positive temperature-dependence of biodiversity–area slopes was consistently recovered for unified species richness (*P* < 0.001) and for functional redundancy in both datasets (unified *P* = 0.035; individually reported *P* = 0.001), whereas the resampling results were weaker for the other biodiversity metrics.

**Extended Data Fig. 4:**
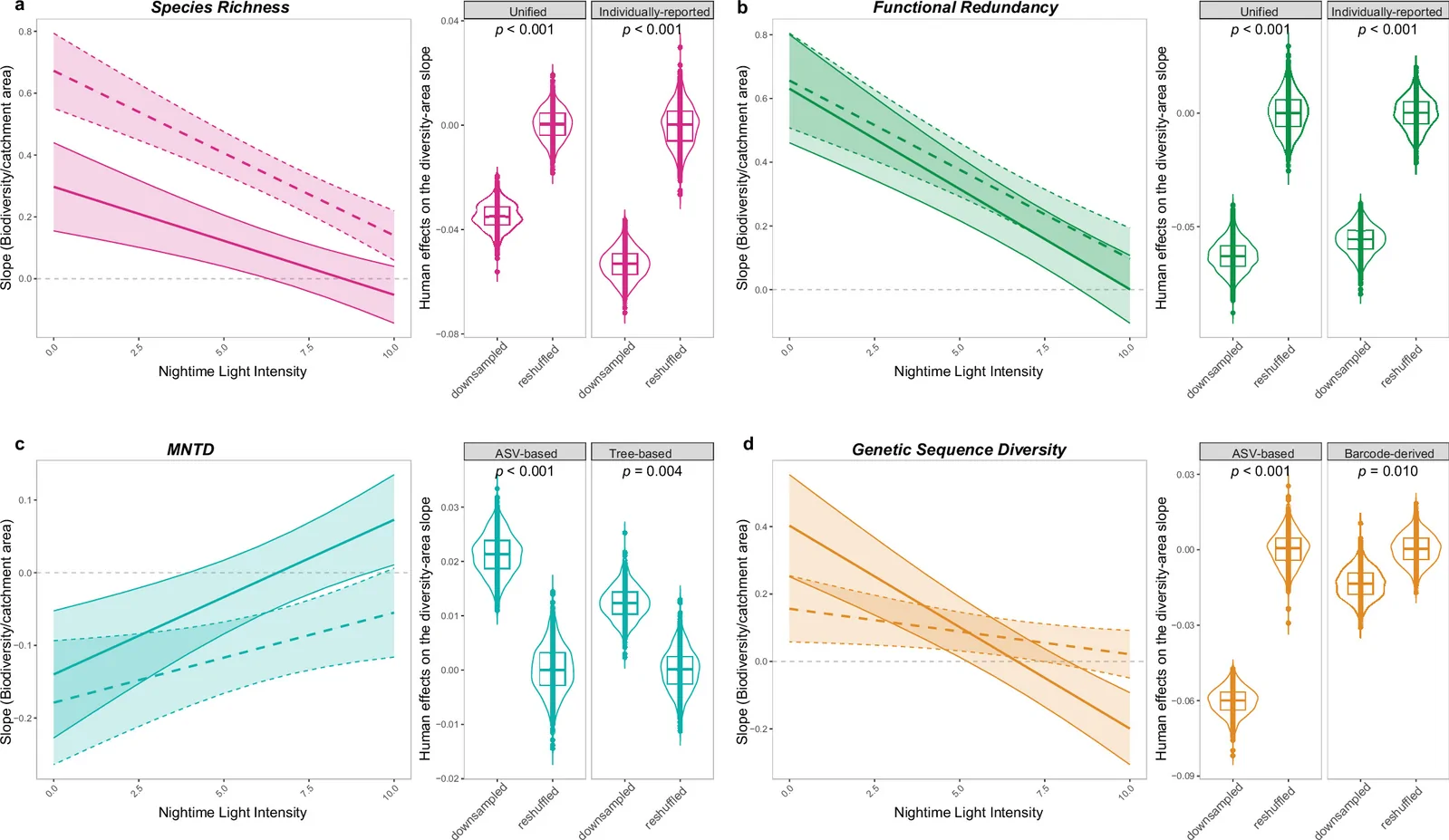
Relationships between nighttime lights and the slope of biodiversity responses to catchment area across four biodiversity facets. Panels show species richness (**a**), functional redundancy (**b**), MNTD (**c**) and genetic sequence diversity (**d**). Slopes were estimated from 1,000 generalized linear mixed model fits using 70% downsampling. Solid lines represent Unified biodiversity metrics; dashed lines represent Original individually-reported or conventional metrics. Shaded areas show 95% confidence intervals, and *p* values reflect the proportion of reshuffled effects exceeding the median estimate. For *p* < 0.001, this indicates that none of the 1000 reshuffling iterations resulted in an estimated slope exceeding the median of the downsampling distribution. For box plots, the centre line indicates the median, the box bounds indicate the 25^th^ and 75^th^ percentiles, and the whiskers extend to the most extreme data points within 1.5 × the interquartile range. Source data

Overall, urbanization (represented by night-time light intensity) exerted a broadly negative effect on biodiversity–area slopes across all facets except MNTD (Fig. 4e–h and Extended Data Table 2). Slopes for species richness and functional redundancy (both unified and individually reported) declined significantly with increasing night-time light intensity (*P* < 0.001), indicating that urbanization reduced the rate at which biodiversity accumulates with increasing area. Genetic sequence diversity followed the same pattern, with significant negative relationships for both ASV-based (*R*^2^ = 0.88, *P* < 0.001) and barcode-derived metrics (*R*^2^ = 071, *P* < 0.001). MNTD again exhibited an opposite response, with slopes for both ASV-based and tree-based MNTD increasing significantly with larger night-time light intensity (*R*^2^ = 0.23, *P* < 0.001 for both metrics), suggesting that phylogenetic community structure became increasingly homogenized across space in more heavily disturbed catchments. These negative effects of night-time light intensity on biodiversity–area slopes were also robust under the downsampling and reshuffling procedures (Extended Data Fig. 4 and Extended Data Table 2).

### Sensitivity to human activities along river network position

The significant interaction between catchment area and night-time light intensity not only reflected the modulation of biodiversity–area relationships by urbanization but also revealed substantial variation in the responses of fish biodiversity to human activities within river networks.

**Fig. 5:**
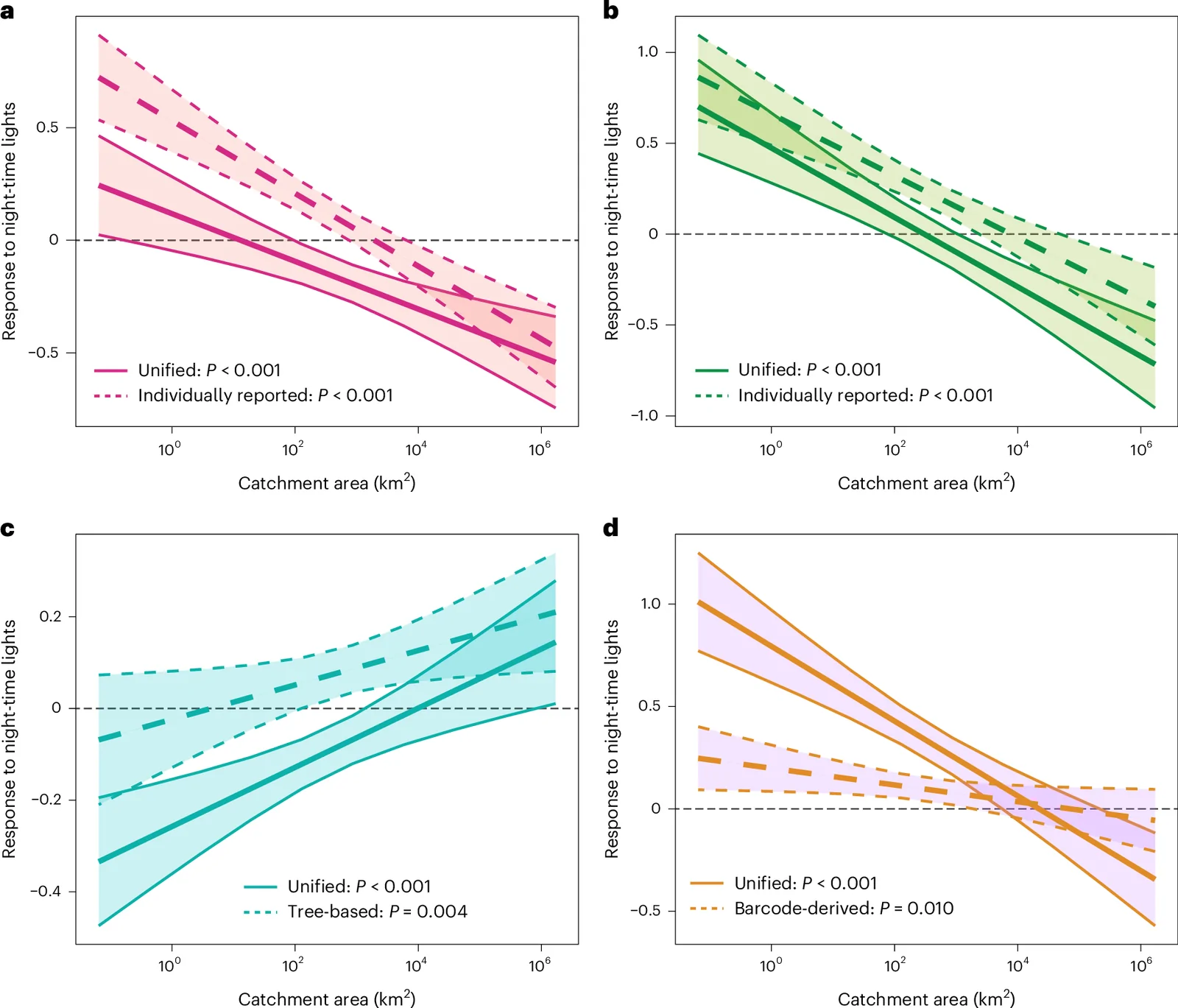
Responses of fish diversity to human activities across river network positions. Relationships between catchment area and diversity–human activities slopes for species richness (**a**), functional redundancy (**b**), MNTD (**c**) and genetic diversity (**d**) estimated from GLMMs based on 1,000 replicates of random 70% downsampling. Human activities were represented by night-time light data, and catchment area was used as a proxy for the position in the river network. Solid lines represent the mean marginal effect of catchment area derived from unified diversity metrics, while dashed lines correspond to the original individually reported or conventional metrics. Shaded areas indicate the 95% confidence intervals of the marginal effects. Slopes reflect the strength and direction of biodiversity responses to night-time light intensity. Negative slopes indicate stronger biodiversity loss with increasing human activities. All *P* values reflect the proportion of reshuffled models (1,000 times), with estimated effects exceeding the median of the downsampling distribution. *P* < 0.001 indicates that none of the 1,000 reshuffling iterations resulted in an estimated slope exceeding the median of the downsampling distribution. Source data

**Extended Data Fig. 5:**
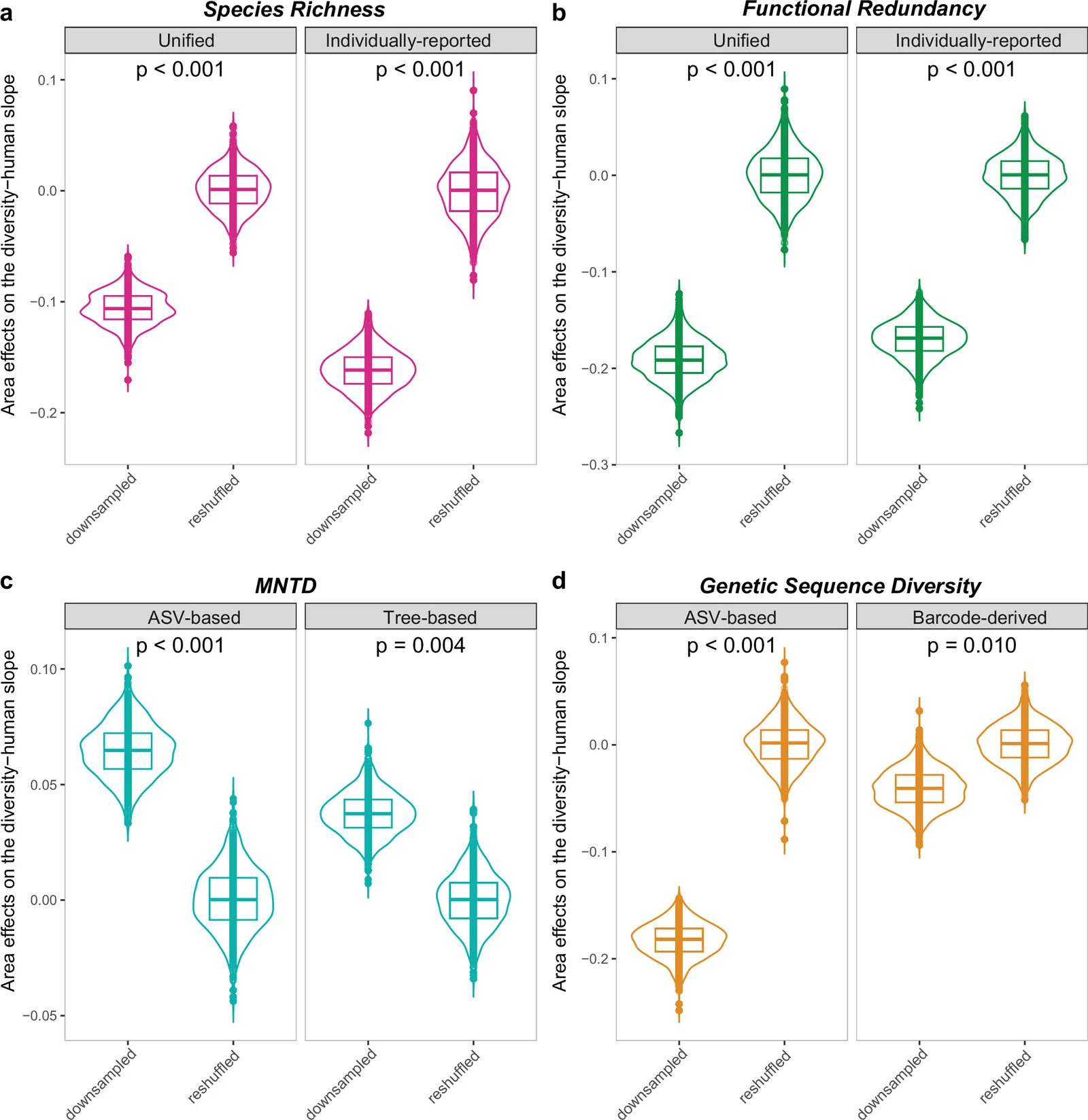
Area effects on diversity–nighttime lights slopes estimated from downsampling and reshuffling procedures. **a–d**, Distributions of area effects on the diversity–nighttime lights slope across four biodiversity facets: (**a**) species richness, (**b**) functional redundancy, (**c**) phylogenetic diversity (MNTD), and (**d**) genetic sequence diversity. For each facet, effects were estimated from 1,000 generalized linear mixed model fits using 70% downsampling (left violin plots) and 1,000 reshuffled replicates (right violin plots). Unified biodiversity metrics are shown on the left of each panel; Original individually-reported or conventional metrics are shown on the right. *p* values indicate the proportion of reshuffled estimates that exceed the median effect size derived from the downsampling procedure. Boxplots within violins represent interquartile ranges and medians of the estimated effects. For *p* < 0.001, this indicates that none of the 1000 reshuffling iterations resulted in an estimated slope exceeding the median of the downsampling distribution. For box plots, the centre line indicates the median, the box bounds indicate the 25^th^ and 75^th^ percentiles, and the whiskers extend to the most extreme data points within 1.5 × the interquartile range. Source data

Biodiversity responses to night-time light intensity exhibited clear and facet-specific dependence on river network position (Fig. 5). For species richness, functional redundancy and genetic sequence diversity, the slopes of biodiversity–night-time light intensity relationships were significantly more negative in smaller rather than larger catchments (*P* = 0.010 for barcode-derived genetic sequence diversity, and *P* < 0.001 for others). This indicated that the negative impacts of urbanization were stronger in larger catchments, typically representing downstream sections of river networks, whereas headwater streams with smaller catchment areas experienced comparatively weaker negative impacts. In contrast to the other biodiversity facets, the slope of the MNTD–night-time light intensity relationship increased significantly with catchment area (*P* < 0.001; tree-based *P* = 0.004), suggesting that the negative effects of night-time light intensity on phylogenetic diversity were strongest in smaller catchments, leading to pronounced phylogenetic homogenization, but these effects weakened as catchment area increased, even shifting toward neutral or positive responses in larger catchments. These patterns were consistent and statistically robust, as revealed by the downsampling-based modelling combined with reshuffling-based *P* value estimation (Extended Data Fig. 5).

## Discussion

As a global-scale assessment of riverine fish biodiversity using unified eDNA data collected from 1,818 sampling sites across 113 river systems, we derived diversity patterns across taxonomic, functional, phylogenetic and genetic facets of fish across global river systems. We find that eDNA-based biodiversity assessments, based on a meta-analysis of individual studies, capture regional to global riverine fish diversity patterns that can be used for attributing state and change of biodiversity and, thus, provide guidance for conservation measures. Further, we demonstrate that the common method of eDNA analysis captured associations of biodiversity patterns with environmental drivers (temperature and catchment area) as well as human activity impacts (both urbanization and agriculture). Biodiversity–area relationships were strongly shaped by drainage characteristics, with an amplification of this scaling along a temperature gradient, while human activities—driven primarily by urbanization—were disrupting it. In parallel, biodiversity responses to human activities exhibited clear spatial heterogeneity. Species richness, functional redundancy and genetic sequence diversity experienced stronger negative impacts in larger catchments, whereas phylogenetic diversity showed the strongest negative effects in smaller catchments. These findings show that river network spatial structure governs biodiversity scaling, while it also predicts where human activity impacts are strongest. Practically, it helps to derive how conservation actions and eDNA-based monitoring should be tailored to catchment size and network position, with priorities differing between large catchments and smaller tributaries and headwaters.

Environmental DNA provides a powerful approach to simultaneously assess multi-faceted fish biodiversity across global rivers. A growing body of research has explored global patterns of freshwater fish biodiversity, most often focusing on taxonomic diversity and its environmental drivers^10,44–47^. Some studies have extended to functional and phylogenetic diversity by incorporating trait data or applying well-established global fish phylogenies^10,45^, advancing our understanding of freshwater biodiversity distribution and threats^48,49^. However, most existing efforts rely on datasets with coarse spatial resolution or biased geographic representativeness, with limited information on genetic diversity^5,50–52^. We demonstrate that eDNA data, when processed through a standardized analytical framework, can overcome many of these constraints by enabling simultaneous assessments of taxonomic, functional, phylogenetic and genetic sequence diversity, thereby providing a valuable supplement to existing global data on fish biodiversity distribution^30,53^. Despite the short length and locus-specific nature of eDNA barcodes, our analysis shows that they can recover global patterns in species richness, as well as approximate phylogenetic and sequence diversity structures consistent with existing knowledge for fishes. Notably, our genetic diversity analyses specifically focus on within-species sequence variation, rather than between-species divergence. In eDNA analyses, it may not be feasible to use different unlinked markers for separate steps of species assignment versus within-species diversity estimation, because the physical linkage required to connect taxonomic assignment to a different locus is not available in environmental samples^54^. Therefore, the locus-specific nature and varying amplicon lengths of these markers should be considered when interpreting these within-species diversity proxies. In our dataset, the genetic sequence diversity estimates from the COI, 12S and 16S barcodes are highly correlated with ASV-based genetic diversity, despite their different evolutionary rates and substitution processes^55^. These markers, chosen for their effectiveness in species barcoding^30,56^, offer a first approximation of genetic diversity, though locus-specific selection pressures and varying amplicon lengths should be considered and caution generalization. While we do not aim for complete spatial or taxonomic coverage, we provide a proof-of-principle that such harmonized, multi-faceted assessments are feasible and can yield generalizable patterns. As the availability of eDNA data continues to grow, this framework can be readily scaled up, offering a path toward increasingly comprehensive and comparable global biodiversity assessments^42^.

Moreover, our unified analysis of eDNA datasets allows us to attribute fish biodiversity distribution to both natural and human-related drivers, highlighting variations in their effects across different biodiversity facets. The biodiversity–area relationship, a classic pattern in riverine ecology, exhibits contrasting trends across regions and studies, with positive^24^, negative^21^ or non-significant^22^ relationships reported. Our results demonstrate that species–area relationship slopes are strongly influenced by climate change and human activities. In line with the species–energy hypothesis, we find that species–area relationship slopes (except MNTD) are generally steeper in tropical regions than in temperate ones, confirming previous expectations^57^. Further, human activities generally weaken the species–area relationship, which is consistent with previous studies suggesting that human impacts reduce the likelihood of detecting rare, vulnerable or habitat-specialist species, thereby flattening species–area relationship slopes^24,58^. In our case, this weakening was primarily associated with urbanization, whereas agriculture showed no significant effect. This generally aligns with recent reports of the opposite effects of agricultural and urban land use on riverine fish assemblages, highlighting that the direction and strength of land-use impacts may be context-dependent^59^. Notably, this weakening effect is facet-dependent, with MNTD exhibiting a weaker or even negative relationship with catchment area, reflecting the loss of unique lineages in larger catchments (for example, in the western Amazon^60^). Such facet-dependent responses highlight the need for multi-faceted biodiversity assessments, as different dimensions of biodiversity exhibit fundamentally distinct and scale-dependent responses to environmental change^34^.

Integrating previously isolated eDNA datasets enables standardized, multi-faceted assessments of fish biodiversity and fosters global collaboration for biodiversity monitoring. Given the massive global human impacts on biodiversity^11^, we need rapid assessment and attribution of state and change of biodiversity at both regional and global scales^39^. Such monitoring and general assessments are also central to many of the goals of the Kunming–Montreal Global Biodiversity Framework^37^. Yet, achieving such common assessments has hitherto been challenged by methodological constraints, and many datasets—once collected—remain isolated. In this study, we show that these common assessments can be achieved for eDNA datasets. By integrating previously isolated datasets through eDNA, we carried out a unified analysis that enabled us to capture multi-faceted distribution patterns of fish biodiversity that are consistent with conventional biodiversity data, despite methodological differences. Specifically, the use of eDNA not only enabled standardized sampling^42^, but also allowed us to integrate the raw data—millions of sequences generated from environmental samples—into a coherent biodiversity synthesis. Beyond technical standardization, our study unites eDNA researchers globally and demonstrates the potential of unified analyses of isolated eDNA datasets. We therefore encourage the development of more adaptable and sustainable data-sharing and collaboration networks, providing a pathway to contribute meaningfully to global biodiversity monitoring and conservation efforts.

In summary, using an extensive integration and unified analysis of 58 individual datasets using eDNA globally, we demonstrate that this method provides an effective and scalable approach for capturing riverine fish biodiversity across taxonomic, functional, phylogenetic and genetic facets, and in particular allows standardized analyses under common frameworks, including identification, taxonomy and sampling efforts. This integrated and unified analysis of eDNA patterns reliably revealed how biodiversity patterns were shaped by major climate factors and human activities, and, thus, can be directly used to understand and manage freshwater biodiversity. These insights underscored the necessity for conservation frameworks to explicitly account for the influence of climate and human activities on biodiversity patterns and the contrasting vulnerabilities of biodiversity facets.

## Methods

### Fish eDNA data and biodiversity metrics

We compiled a global eDNA fish dataset (for detailed methods and analytical approaches, see also Supplementary Methods and ref. ^43^), which integrated both original individually reported and unified fish community data from 113 river catchments worldwide, encompassing 1,818 sampling sites (Fig. 1a). The dataset included both published and unpublished individual eDNA studies that assess riverine fish diversity across six continents, covering the period 2012–2023. Given between-study variation in marker choice and laboratory design, we compiled record-level methodological descriptors, including primer set, barcode region, reported sample volume and technical replication. The 58 datasets collectively employed 23 primer sets spanning four barcode regions (12S, 16S, COI and cytochrome *b*), and 12S assays such as MiFish and teleo or Tele02 were used most frequently. Reported sample volumes ranged from 0.05 l to 400 l, and technical replication ranged from 1 to 36 (Supplementary Table 1). For datasets providing raw sequencing reads, we applied a standardized bioinformatic workflow to generate unified site-by-species matrixes comparable across studies. To reduce between-study annotation bias, we further constrained species assignments using basin-level fish historical occurrence records (Supplementary Methods). In summary, for each site we obtained either (1) an original individually reported species richness or community matrix extracted from previous studies, (2) a unified community matrix generated from standardized processing of raw sequencing data or (3) both original individually reported and unified data.

To quantify biodiversity across river catchments, we assessed four complementary facets, including taxonomic, functional, phylogenetic and genetic (sequence) diversity. Species richness was calculated from both the original individually reported and the unified datasets based on presence records, with 51 out of 113 river catchments having both values available. Functional diversity was derived for both datasets using species-level morphological traits obtained from the FISHMORPH database^61^. Morphological traits were chosen because they provide more comprehensive and accessible information than ecological, physiological and behavioural traits^61^. While they do not fully capture all functional aspects, they serve as indicators of certain functions^62^. Therefore, we clarify that these traits act as informative proxies for functional strategies, rather than direct functional evidence. All trait values are continuous and were, therefore, standardized, and dimensionality reduction was conducted using principal component analysis with Euclidean distance^63^. The number of retained axes was determined by parallel analysis implemented in the ‘paran’ R package^64^. Functional redundancy, which quantifies the degree of trait overlap among species within a community, was computed using the trait probability density framework^26^. Species-level trait distributions were modelled using multivariate kernel density estimation via the ‘ks’ package^65^, and aggregated into community-level distributions using the ‘TPD’ package^66^.

Phylogenetic and genetic sequence diversity was assessed only for unified datasets, as these were the ones for which raw sequencing data were available, which was required to generate ASVs. For phylogenetic diversity, ASV sequences were aligned using MUSCLE with default parameters^67^, and phylogenetic trees were constructed from the aligned sequences using the ‘make_phylogeny.py’ script in QIIME. We then calculated MNTD, defined as the average phylogenetic distance between each taxon and its closest non-conspecific relative:

$$\mathrm{MNTD}=\frac{1}{n}\mathop{\sum }\limits_{i=1}^{n}\mathop{\min }\limits_{j\ne i}{d}_{{ij}}$$

where *d*_*ij*_ denotes the pairwise phylogenetic distance between taxa *i* and *j*, and *n* is the number of taxa in the community. MNTD was calculated using the ‘mntd()’ function from the ‘picante’ package^68^. We also extracted the list of species identified in the unified dataset and retrieved their known phylogenetic relationships from a previously published global phylogeny of freshwater fishes using the ‘FishPhyloMaker’ package^69^. Using this species-level phylogeny, we calculated the phylogenetic diversity following the same method as for the tree-based MNTD.

Genetic sequence diversity was quantified as within-species nucleotide diversity (*π*ₛ). This metric primarily captures sequence-level genetic variation, without explicitly considering haplotype richness or frequency distribution^70^. However, given the nature of eDNA metabarcoding data, where accurate genotype resolution is constrained and allele calling is infeasible, *π*ₛ provides an effective, sensitive and scalable proxy for within-species genetic diversity^36^. In detail, for each species with multiple sequences, we computed the average pairwise sequence divergence as:

$${\pi }_{{\rm{s}}}=\frac{1}{\left(\begin{array}{l}n\\ 2\end{array}\right)}\mathop{\sum }\limits_{i=1}^{n-1}\mathop{\sum }\limits_{j=i+1}^{n}\frac{{k}_{{ij}}}{{m}_{{ij}}}$$

where *k*_*ij*_ is the number of differing base pairs, *m*_*ij*_ is the number of shared base pairs between sequences *i* and *j*, and *n* is the number of sequences per species. Only sequence pairs with more than 50% alignment overlap were retained. The overall genetic sequence diversity (GD) was calculated as the mean nucleotide diversity across all species:

$${\rm{GD}}=\frac{1}{l}\sum {\pi }_{{\rm{s}}}$$

where *l* is the number of species with valid sequence data. We further obtained COI sequences from NCBI for the species identified in the unified data, following the approach in ref. ^47^. COI was selected for its high resolution in species discrimination. Only deduplicated sequences longer than 600 bp were retained, and *π*ₛ was calculated as described above. Across 520 species, the number of qualifying COI sequences per species ranged from 1 to 215 (median = 8, interquartile range = 3–18; Supplementary Table 4). Nucleotide diversity was calculated for the 460 species with at least two sequences (range = 2–215, median = 9, interquartile range = 4–21). For each species, we use all available COI data as a first approximation of within-species genetic diversity, acknowledging that this does not control for different sampling efforts between species or for the geographic distribution of barcode sequences, and, thus, may not completely reflect a species’ local genetic diversity, but is rather an integrated metric. Given that most ASVs targeted 12S and 16S barcode regions, we also calculated nucleotide diversity based on sequences from these markers to enable cross-marker comparison.

In summary, a total of 661 fish species were included in the unified eDNA datasets for quantifying species richness and functional redundancy (Supplementary Fig. 3a). All species were used to calculate tree-based phylogenetic diversity, while genetic sequence diversity was computed for 520 species. The original individually reported diversity data encompassed 1,521 species, although a substantial proportion (517 species) were identified only to the genus level (Supplementary Fig. 3b). As a result, functional diversity calculations based on the original individually reported datasets were limited to 782 species with validated species-level trait information.

### Validation of biodiversity metrics derived from eDNA

We used GLMMs to assess whether biodiversity metrics derived from eDNA reflect global patterns of freshwater fish diversity^71^. For taxonomic, phylogenetic and genetic sequence diversity, we separately modelled the eDNA-inferred values as a function of the corresponding reference values, while accounting for sampling and technical variation (Fig. 2a). For taxonomic diversity, catchment-level species richness estimated from eDNA was modelled as a function of observed richness compiled from historical occurrence records^49^. For phylogenetic and genetic sequence diversity, MNTD and nucleotide diversity derived from ASVs were compared respectively with MNTD calculated from a species-level phylogeny and with COI-based genetic sequence diversity obtained from public databases. Filtered water volume was included as a fixed covariate in all models to account for differences in sampling effort. Amplification-related variations were modelled by including either primer identity or barcode region as a random effect. All response and predictor variables were log-transformed (base 10) before model fitting. Models were fitted using the ‘glmmTMB’ package in R^72^.

### Delineation of river catchments and extraction of drainage characteristics

To better capture the river catchment characteristics, we employed the widely used D8 algorithm and the Hydrography90m flow direction map (3 arc-second resolution, ~90 m at the equator) to calculate the upstream catchment for eDNA sampling sites^73^. In brief, the value of a pixel in the flow direction map indicates the water flow direction to eight adjacent pixels. To begin with, river channels were directly extracted from the flow accumulation map of Hydrography90m, with which we manually checked and calibrated the coordinates of all the eDNA sampling sites. By tracking along the water flow direction map, we can determine whether a terrestrial pixel ultimately flows through a sampling site and then calculate its water flow distance. These terrestrial pixels that flowed through the sampling site were then connected to produce a site catchment map (Supplementary Fig. 4). Because of worldwide spatial coverage of sampled rivers, we used the haversine formula to estimate the length and width of map pixels for better accuracy in the flow distance computation. In addition, we calculated the catchment area for all the sampling sites based on these catchment maps.

We used the WorldClim version 2.1 climatic data (30-second resolution) for the period 1970–2000 to assess the climatic effects on global fish distribution patterns (https://www.worldclim.org/data/worldclim21.html)^74^. We evaluated multiple BIOCLIM temperature variables representing temperature extremes and seasonality. Because these predictors were moderately to strongly correlated with one another and with mean annual temperature (|*r*| > 0.6; Supplementary Fig. 5), we used mean annual temperature as a parsimonious and widely used summary temperature metric to reduce collinearity and maintain interpretability.

We considered multiple available human-related variables, including human population density, percentage cropland cover (% cropland) and the Global Human Modification index, capturing various components of human activity. Night-time light data were used as a typical proxy for urbanization, and we directly used the night-time light layer from refs. ^75,76^, which was developed based on the Defense Meteorological Satellite Program Operational Linescan System night-time light data. Human population density was mapped using the Gridded Population of the World dataset developed by the Centre for International Earth Science Information Network^76,77^. Cropland cover was extracted from the 300-m resolution land cover map developed by the European Space Agency Climate Change Initiative^78^. The Global Human Modification index was derived from ref. ^79^, which quantified the degree of land modification by modelling the physical extents of 13 different anthropogenic stressors. To better represent the human activities from the surrounding upstream area, we extracted the above variables within a 4 km upstream flow distance buffer for each sampling site and then calculated the median as the estimates. Human population density and Global Human Modification were found to be highly correlated with night-time lights (*r* = 0.61 and *r* = 0.73, respectively; Supplementary Fig. 5), while cropland cover showed weaker associations (*r* = 0.13). Based on these correlations, we ultimately selected two variables as human activity proxies: night-time light as an urbanization proxy and cropland cover as an agricultural proxy.

### Statistical modelling of biodiversity responses

To evaluate how drainage characteristics influence fish biodiversity across river networks, we used GLMMs with a beta distribution and logit link function, implemented via the ‘glmmTMB’ package in R^72^. The response variables included species richness, functional redundancy, MNTD and genetic sequence diversity. These biodiversity metrics exhibited right-skewed distributions and varied in scale (Fig. 1 and Supplementary Table 5). Although their measurement units and ranges vary, all metrics share a common statistical property in that they are continuous and constrained within a fixed interval. This supports the use of beta distribution as a consistent and appropriate modelling approach across biodiversity metrics^80^. Accordingly, before modelling, all response variables were log-transformed to stabilize variance and subsequently rescaled to the interval between zero and one. The beta distribution was selected for its capacity to accommodate continuous, bounded and asymmetric data structures, offering improved flexibility and robustness compared to Gaussian alternatives^81^. Logit link allowed covariate effects to be estimated on an unbounded latent scale while preserving the ecological interpretability of predictions within a bounded outcome space^82^.

The null model accounted for amplification and sampling variation by including sampling volume as a covariate, spatial autocorrelation as a fixed effect and a random intercept for either primer or barcode region. Spatial autocorrelation was defined as the average minimum distance between sampling sites within each river catchment, reflecting local clustering while accounting for heterogeneity in catchment extent. Notably, few (19 out of 113) river catchments reported multiple observations at identical or near-identical coordinates due to multi-marker assays and/or repeated sampling across seasons or years. Because this can create non-independence and overweight a site, we did not merge these a priori. Instead, we accounted for them within the null model. The basic model added catchment area as a proxy for network position, assuming biodiversity scales with drainage area due to cumulative dispersal, environmental filtering and habitat complexity. An interaction between catchment area and spatial autocorrelation was included to account for differences in the spatial structure of sampling within catchments. The full model further incorporated climate- and human-activity-related variables, including their interactions with catchment area. Specifically, we included mean annual temperature as a proxy for climate, while night-time light intensity and cropland cover served as proxies for human activity, and tested whether spatial scaling relationships vary along climate or human activity gradients using interactions with catchment area. Nested models were compared using likelihood ratio tests to evaluate whether the inclusion of additional terms significantly improved model fit^83^ (Supplementary Table 6). Based on the results, we excluded the interaction between catchment area and cropland cover from the final model because likelihood ratio tests showed it was generally non-significant for the biodiversity metrics and its inclusion could lead to overparameterization (Supplementary Table 3). Therefore, the final model retained mean annual temperature, night-time light intensity and cropland cover, as well as the interactions between catchment area and mean annual temperature and between catchment area and night-time light intensity.

Models were fitted independently for each biodiversity metric. All predictor variables were standardized by mean-centering and scaling to unit variance, with catchment area log_10_-transformed before standardization (Supplementary Fig. 6). Model fit was evaluated by plotting fitted versus observed values for each diversity metric (Supplementary Fig. 7). Models captured the overall variation reasonably well (generalized additive model: *R*^2^ = 0.15–0.43), consistent with typical expectations for noisy ecological data. Model performance was further assessed using Akaike information criterion, Bayesian information criterion, log-likelihood and both marginal and conditional *R*^2^ values. Based on these comparisons, primer identity was used as the random intercept for species richness and functional redundancy, while barcode region was used for phylogenetic and genetic sequence diversity (Supplementary Fig. 8). Fixed-effect estimates were extracted to assess the relative importance of predictors across biodiversity metrics.

### Prediction and interpretation of model outputs

To examine the climatic and human activity dependence of biodiversity scaling with catchment area, we performed a series of marginal slope predictions based on the full model structure. First, for each biodiversity metric, we evaluated how climatic variable (mean local annual temperature) and intensity of urbanization (approximated by night-time light intensity) modulate the relationship between biodiversity and catchment area. Median values of annual temperature, night-time light intensity and spatial autocorrelation were calculated for each river catchment by aggregating all sampling sites within the corresponding drainage area. These aggregated values defined the climatic and human activity conditions under which the biodiversity–area relationship was evaluated. Using the ‘emtrends()’ function from the ‘emmeans’ package^84^, marginal trends in biodiversity as a function of catchment area were predicted for each catchment.

To evaluate the uncertainty of slope estimates, we repeatedly performed full model fitting followed by marginal slope estimation, each based on a random subsample comprising 70% of the total sites. This process was repeated 1,000 times. In a parallel analysis, we assessed whether responses of biodiversity to human activities vary along the river network by predicting the marginal slope of biodiversity in response to night-time light intensity across a standardized gradient of catchment area. A grid of values spanning the observed range of catchment size was used to generate these predictions.

To test whether observed biodiversity–area scaling arises from genuine ecological patterns rather than from spatial autocorrelation in covariates, we implemented a covariate reshuffling procedure repeated 1,000 times. In each iteration, temperature and night-time light intensity were randomly reassigned among sampling sites. Slope estimates under reshuffling were compared to those from downsampling-based predictions, and *P* values were calculated as the proportion of reshuffles with slopes exceeding the median from the downsampling procedure^85^ (Supplementary Fig. 9).

### Reporting summary

Further information on research design is available in the Nature Portfolio Reporting Summary linked to this article.

## Supplementary information

Supplementary Information Supplementary Methods, Tables 4–6 and Figs. 1–9.

Reporting Summary

Peer Review File

Supplementary Tables Supplementary Table 1–3.

## Source data

Source Data Fig. 1 Statistical source data.

Source Data Fig. 2 Statistical source data.

Source Data Fig. 3 Statistical source data.

Source Data Fig. 4 Statistical source data.

Source Data Fig. 5 Statistical source data.

Source Data Extended Data Fig. 1 Statistical source data.

Source Data Extended Data Fig. 2 Statistical source data.

Source Data Extended Data Fig. 3 Statistical source data.

Source Data Extended Data Fig. 4 Statistical source data.

Source Data Extended Data Fig. 5 Statistical source data.

## Figures and Tables

**Extended Data Table 1:**
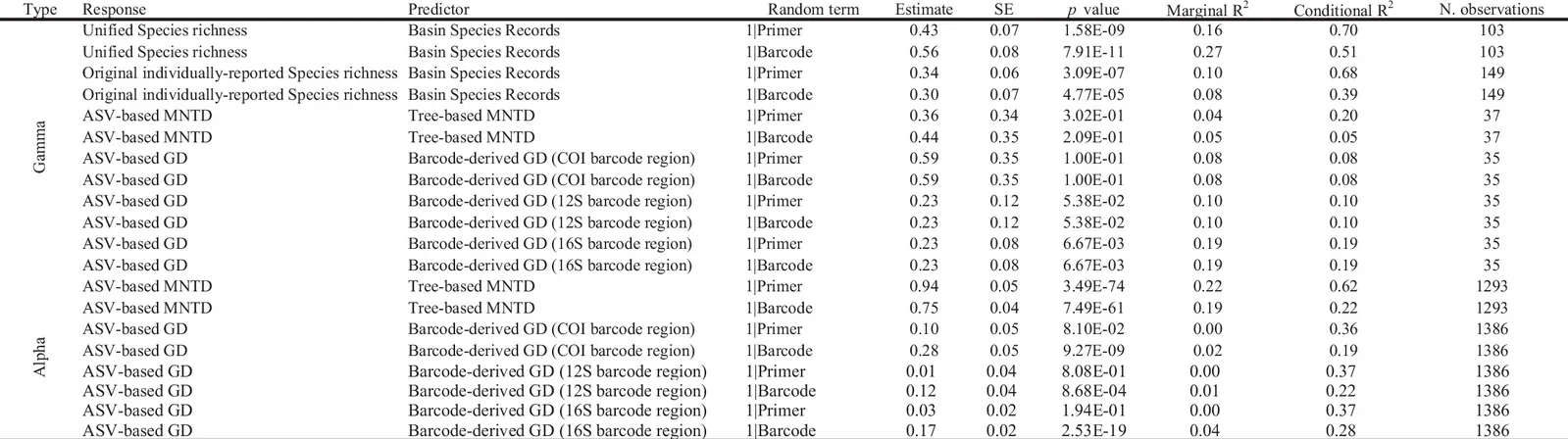
Summary of modelled relationships modelled using generalized linear mixed models, with significance assessed using two-sided Wald tests, between eDNA-based metrics and traditional biodiversity references

**Extended Data Table 2:**
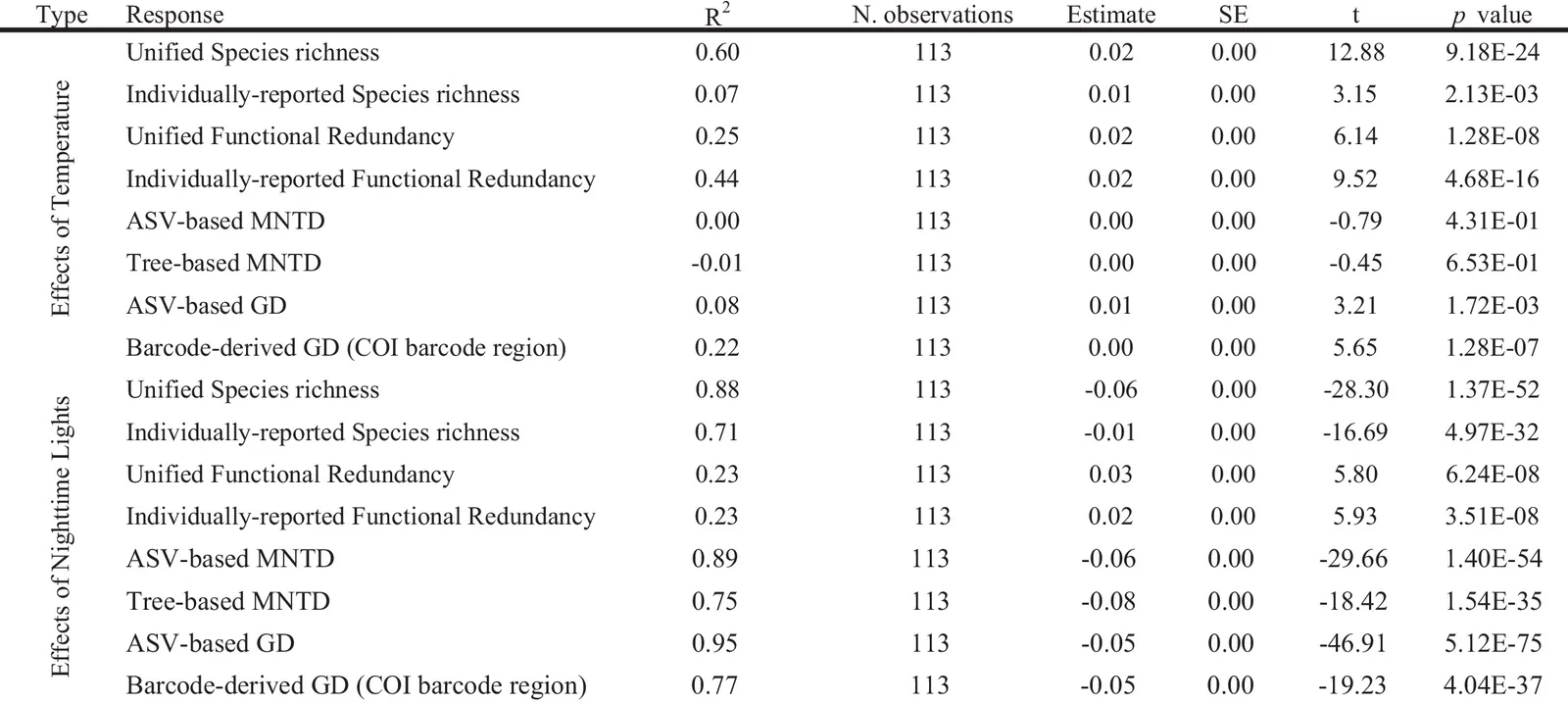
Summary of generalized linear models examining the effects of temperature and nighttime lights density on biodiversity–area relationships across river catchments

The *p* values for model coefficients were calculated using two-sided tests.

## Data Availability

The unified and standardized dataset for global riverine fish eDNA meta-analysis is available via Dryad at https://doi.org/10.5061/dryad.gf1vhhn2h (ref. ^86^). The minimum dataset required to interpret, verify and extend this study is available via GitHub at https://github.com/Laura61616/GRiversFisheDNA_Main. Source data are provided with this paper.

## Code availability

All custom code used for data processing, analysis and figure generation is publicly available via GitHub at https://github.com/Laura61616/GRiversFisheDNA_Main.
