# Supplementary material for: A unified analysis of global riverine eDNA reveals common associations of fish biodiversity with drainage characteristics
Source: Nat Ecol Evol. 2026 Jun 24;10(10):1927–40. doi: 10.1038/s41559-026-03106-1 (PMC13658041; doi:10.1038/s41559-026-03106-1)
Supplement: Supplementary file 1 [file 41559_2026_3106_MOESM1_ESM.pdf]

nature ecology & evolution

Supplementary information https://doi.org/10.1038/s41559-026-03106-1

A unified analysis of global riverine eDNA reveals common associations of fish biodiversity with drainage characteristics

In the format provided by the authors and unedited

Table of contents

Supplementary Methods

Datasets compilation

Bioinformatics Workflow

Supplementary Tables

Supplementary Table 4 Summary of sequences used to calculate barcode-driven genetic diversity.

Supplementary Table 5 Summary of biodiversity metrics. Skewness for each response factor was assessed using a two-sided D'Agostino test of skewness.

Supplementary Table 6 Model comparisons for testing the effect of drainage charateristic using likelihood ratio tests.

Supplementary Figures

Supplementary Fig. 1 Correlation plots of log-transformed multi-faceted fish biodiversity metrics. Correlation analysis was performed using Pearson's correlation tests. The numbers and colors represent the correlation coefficients.

Supplementary Fig. 2 Latitudinal distribution of multi-faceted biodiversity. The regression lines were fitted using a generalized additive models with a smooth term (k=2). The shaded area represents the 95% confidence interval of the fit. *P* values are two-sided and test whether the corresponding regression coefficient differs from zero.

Supplementary Fig. 3 Summary of species included in the Unified and Original individually-reported datasets used to compute multi-faceted fish biodiversity.

Supplementary Fig. 4 Example illustrating the delineation of sub-catchments for individual sampling sites.

Supplementary Fig. 5 Correlation plots of climatic (a) and human-related (b) factors. Correlation analysis was performed using Pearson's correlation tests. The numbers and colors represent the correlation coefficients.

Supplementary Fig. 6 Density plots of drainage charateristics.

Supplementary Fig. 7 Fitted vs observed values of the full models. The regression lines were fitted using generalized linear models with a Gaussian error distribution. Reported p values are two-sided and test the null hypothesis that the slope coefficient equals zero. Adjusted R^2 is the standard coefficient of determination adjusted for the number of predictors. The shaded area represents the 95% confidence interval of the fit.

Supplementary Fig. 8 Marginal and conditional R^2 values for generalized linear mixed models fitted to each biodiversity metric. The model included catchment area, spatial autocorrelation, temperature, nighttime light intensity, and sample volume as fixed effects, with interactions between catchment area and environmental predictors. Barcode (a) or primer (b) was used as the random term.

Supplementary Fig. 9 Examples showing predicted slopes derived from downsampling and reshuffling procedures.

Supplementary Methods

1. Datasets compilation

We conducted a comprehensive literature search on 13 December 2022 using the Web of Science Core Collection to identify studies applying environmental DNA (eDNA) metabarcoding for assessing fish communities in riverine ecosystems. The search query was "TS = (monitor OR assessment OR communit* OR richness) AND TS = (molecular OR 'environmental DNA' OR eDNA OR metabarcoding OR 'high throughput sequencing' OR 'high-throughput sequencing' OR HTS OR 'next generation sequencing' OR 'next-generation sequencing' OR NGS) AND TS = (stream$ OR river$ OR estuar*) AND TS = (fish OR Actinopteri*) AND PY = (2010–2025) NOT TS = (qPCR OR ddPCR) NOT TI = (soil OR diet OR dietary)." In this formulation, "TS" refers to topic fields (title, abstract, and keywords), "TI" to title, and "PY" to publication year. The wildcard asterisk represents zero or more characters, while the dollar sign denotes zero or one character.

The search yielded 673 publications. After removing 13 book chapters, 660 records were manually screened. Only original research papers were retained, with reviews and meta-analyses excluded. To ensure ecological and methodological relevance, studies were required to focus on fish communities in rivers or streams and to employ eDNA metabarcoding as the primary tool for biodiversity assessment. Fifty papers met these criteria. A second round of screening was conducted to ensure that sufficient

information was available for data extraction; only studies providing geographic coordinates for sampling sites and reporting species richness or raw sequencing data were retained. Forty-two papers met all inclusion criteria and were incorporated into the initial dataset.

To expand coverage beyond the published literature, we issued an open call for additional data through the social media platform X (formerly Twitter), inviting researchers and institutions conducting similar eDNA-based studies to share unpublished or otherwise inaccessible datasets. This outreach resulted in 23 new datasets contributed by co-authors, encompassing 588 sampling sites across 19 river catchments distributed globally: one from Africa, five from Asia, two from Australia, seven from Europe, five from North America, and three from South America. Three of these overlapped with previously collected studies, producing a total of 62 datasets. Four studies represented only a single sampling site and were excluded, yielding a final total of 58 datasets (designated as "DataID") used in the meta-analysis.

For each dataset, we compiled raw sequencing data, site-level species richness tables, and species-by-site matrices. Of these, 41 included raw sequencing data, and several used multiple primer sets. Each unique dataset–primer combination was treated as an independent sequencing dataset, producing 56 distinct raw sequencing datasets archived separately (designated "RawSeqID"). Primer names were standardized by

sequence, and sample identifiers were harmonized across sequencing data, richness tables, and species-by-site matrices. Taxonomic names of fish species were standardized using the R package "rfishbase" [1].

2. Bioinformatics Workflow

For datasets lacking prior demultiplexing, paired-end reads were first joined using VSEARCH [2]. Demultiplexing was then performed with the *barcode_splitter* utility based on barcode sequences, allowing up to three mismatches [3]. Reads that failed to match a barcode or matched more than one were removed. Demultiplexed reads were renamed and saved as individual fastq files corresponding to each sample. When double-index adapters were present, read separation and filtering were additionally handled with *NGSFILTER* implemented in the OBITools package [4].

Subsequent processing of demultiplexed reads was conducted using the DADA2 workflow in R [5]. Reads were imported and filtered to remove ambiguous nucleotides (maximum allowed = 0) using the *filterAndTrim()* function. Primer sequences were detected and excised with CUTADAPT [6], ensuring all sequence orientations were checked. Quality filtering parameters included a maximum expected error threshold (maxEE) of 2 and a truncation quality score (truncQ) of 2. Error models were estimated with *learnErrors()*, and denoising was performed with *dada()* to infer amplicon sequence variants (ASVs). Paired-end reads were subsequently merged using

mergePairs(), and chimeric sequences were identified and removed with *removeBimeraDenovo()*. The resulting sequence table retained only non-chimeric ASVs. Read retention was monitored at each stage of processing (raw, filtered, denoised, merged, and chimera-free).

Reference databases corresponding to each primer set were generated using the CRUX pipeline, implemented as the first module of the Anacapa Toolkit [7]. The workflow begins with an in-silico PCR conducted via ecoPCR on the EMBL nucleotide database to obtain a seed library of taxon-specific sequence identifiers [8,9]. Primer regions are subsequently verified and trimmed using CUTADAPT [6]. To address the frequent absence of primer regions in GenBank entries, CRUX performs two iterative BLAST searches against the NCBI nucleotide database [10]: the first restricted to full-length sequences and the second permitting shorter reads down to 70% of full length. All retrieved sequences are de-replicated, retaining only the longest representatives. Taxonomic assignment is performed using entrez-qiime [11], which maps curated reads to their corresponding taxa. Sequences with incomplete or ambiguous annotations, including those labeled as “unassigned” or “uncultured,” are removed to preserve database accuracy. The final curated reference sets are formatted as Bowtie2 index libraries for downstream analyses.

Taxonomic annotation of ASVs was carried out using the Classifier module of the

Anacapa Toolkit, which integrates *Bowtie2* alignment with a Bayesian Least Common Ancestor (BLCA) algorithm [7,12,13]. ASVs were aligned globally to the CRUX reference database with *Bowtie2*, and the top 100 matches per read were processed with the BLCA classifier. Taxonomic confidence was evaluated using bootstrap scores to retain high-confidence assignments.

To ensure accurate species annotation, we used historical species records to correct the species identification for each eDNA raw sequencing dataset. The historical species records were extracted from a global freshwater fish occurrence database encompassing 3,119 drainage basins worldwide [14]. This dataset compiles comprehensive lists of native, introduced, and invasive species. Each eDNA dataset was linked to its corresponding drainage basin and in cases without a direct match, the geographically nearest basin was used. Only species with documented historical records from the relevant drainage basin were retained for annotation. In this process, we avoided ASV rarefaction as sequencing depth varied significantly across studies.

The standardized processing of raw sequencing data was performed on a local workstation running Ubuntu 18.04 with 48 CPU cores and 125 GB RAM, without GPU acceleration.

Supplementary References

1 Froese, R. & Pauly, D. in *World Wide Web electronic publication* (2002).
2 Rognes, T., Flouri, T., Nichols, B., Quince, C. & Mahe, F. VSEARCH: a

versatile open source tool for metagenomics. *PeerJ* **4**, e2584,

Supplementary Table 4 Summary of sequences used to calculate barcode-driven genetic diversity.

	**COI barcode**		**12S rRNA barcode**		**16S**	
	with ⩾ 1 sequence(s)	*with ⩾ 2 sequences*	*with ⩾ 1 sequence(s)*	*with ⩾ 2 sequences*	*with ⩾ 1 sequence(s)*	*with ⩾ 2 sequences*
Number of species	520	460	462	303	479	311
Minimum	1	2	1	2	1	2
Maximum	215	215	80	80	132	132
Median	8	9	2	3	2	3
Mean	15.74	17.66	4.47	6.30	4.64	6.60
Q1 (25th percentile)	3	4	1	2	1	2
Q3 (75th percentile)	18	21	4	6	4	7

Supplementary Table 5 Summary of biodiversity metrics. Skewness for each response factor was assessed using a two-sided D'Agostino test of skewness.

Response factors	Min.	Quantile 25%	Median	Mean	Quantile 75%	Max.	SD	Zero ratio	Original		Log10-transformed	
									skewness	*p* value	skewness	*p* value
Unified species richness	0.00	2.00	6.00	9.95	11.00	149.00	200.88	0.11	4.24	2.23e-222	0.01	9.26e-01
Unified func. Redundancy	0.00	0.19	0.64	1.04	1.08	15.97	2.64	0.14	4.40	6.28e-202	-0.13	3.88e-02
ASV-based MNTD	0.00	0.06	0.11	0.14	0.18	0.95	0.01	0.00	2.32	1.36e-107	-0.74	0.00e+00
ASV-based Sequence Diversity	0.00	0.00	0.00	0.01	0.01	0.17	0.00	0.46	6.01	5.55e-278	0.55	1.36e-16
Original individually-reported species richness	0.00	4.00	10.00	15.06	21.00	126.00	276.48	0.06	2.44	3.26e-185	-0.36	6.12e-12
Original individually-reported func. Redundancy	0.00	0.32	0.96	1.39	2.05	11.71	2.27	0.12	2.09	2.09e-141	-0.50	0.00e+00
Tree-based MNTD	13.60	110.06	140.14	166.38	219.07	458.88	6234.04	0.00	1.00	3.27e-36	-0.37	9.80e-08
Barcode-derived Sequence Diversity	0.00	0.01	0.01	0.02	0.02	0.22	0.00	0.01	4.49	9.31e-208	-0.26	6.99e-05

Supplementary Table 6 Model comparisons for testing the effect of drainage charateristic using likelihood ratio tests.

Test term	Model 1	Model 2
catchment_area	y ~ spatial_autocor + sample_volume + (1|random)	y ~ catchment_area + spatial_autocor + sample_volume + (1|random)
catchment_area:spatial_autocor	y ~ catchment_area + spatial_autocor + sample_volume + (1|random)	y ~ catchment_area * spatial_autocor + sample_volume + (1|random)
temperature	y ~ catchment_area * spatial_autocor + sample_volume + (1|random)	y ~ catchment_area * spatial_autocor + temperature + sample_volume + (1|random)
catchment_area:temperature	y ~ catchment_area * spatial_autocor + temperature + sample_volume + (1|random)	y ~ catchment_area * spatial_autocor + catchment_area * temperature + sample_volume + (1|random)
nighttime_light	y ~ catchment_area * spatial_autocor + catchment_area * temperature + sample_volume + (1|random)	y ~ catchment_area * spatial_autocor + catchment_area * temperature + nighttime_light + sample_volume + (1|random)
catchment_area:nighttime_light	y ~ catchment_area * spatial_autocor + catchment_area * temperature + nighttime_light + sample_volume + (1|random)	y ~ catchment_area * spatial_autocor + catchment_area * temperature + catchment_area * nighttime_light + sample_volume + (1|random)
%cropland	y ~ catchment_area * spatial_autocor + catchment_area * temperature + catchment_area * nighttime_light + sample_volume + (1|random)	y ~ catchment_area * spatial_autocor + catchment_area * temperature + catchment_area * nighttime_light + %cropland + sample_volume + (1|random)
Catchment_area:%cropland	y ~ catchment_area * spatial_autocor + catchment_area * temperature + catchment_area * nighttime_light + %cropland + sample_volume + (1|random)	y ~ catchment_area * spatial_autocor + catchment_area * temperature + catchment_area * nighttime_light + catchment_area * %cropland + sample_volume + (1|random)

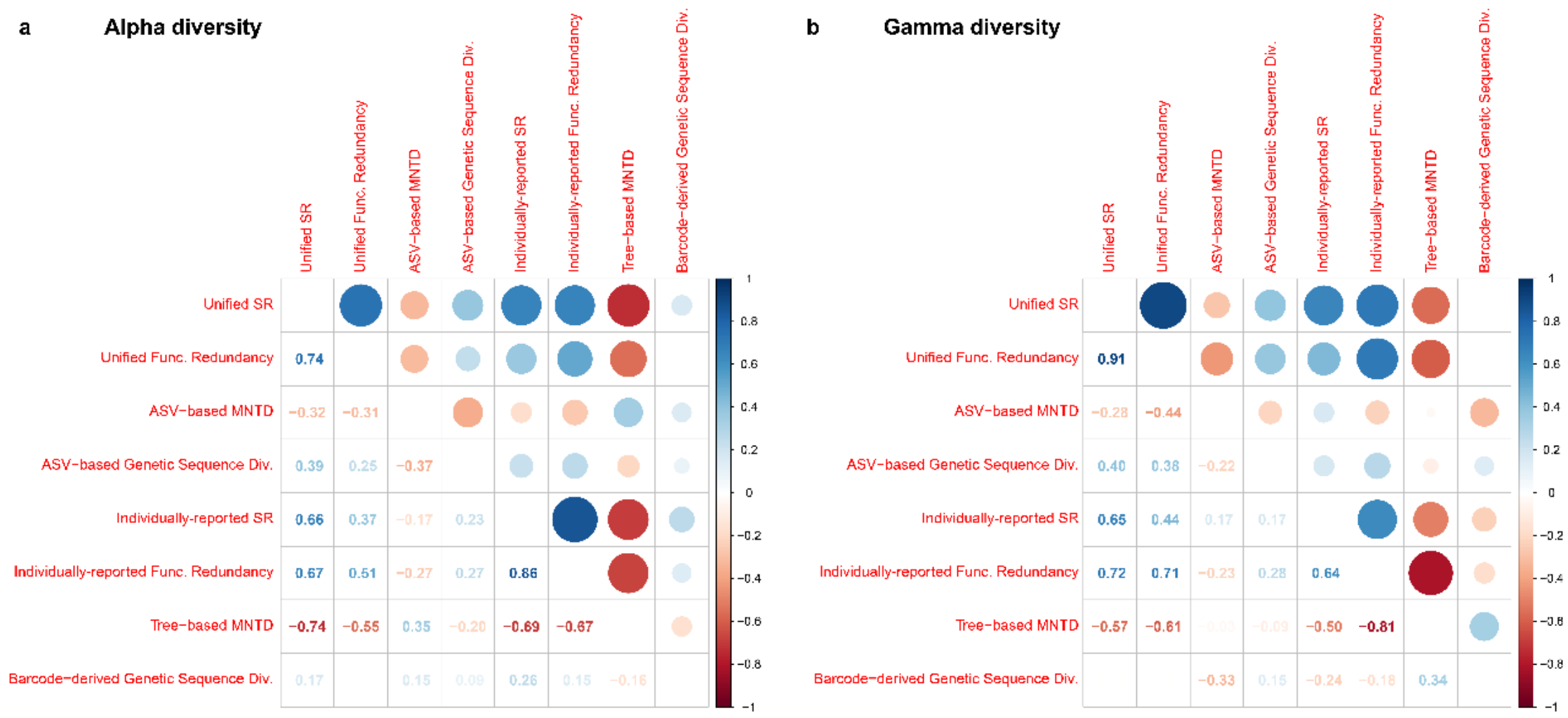

Supplementary Fig. 1 Correlation plots of log-transformed multi-faceted fish biodiversity metrics. Correlation analysis was performed using Pearson's correlation tests. The numbers and colors represent the correlation coefficients.

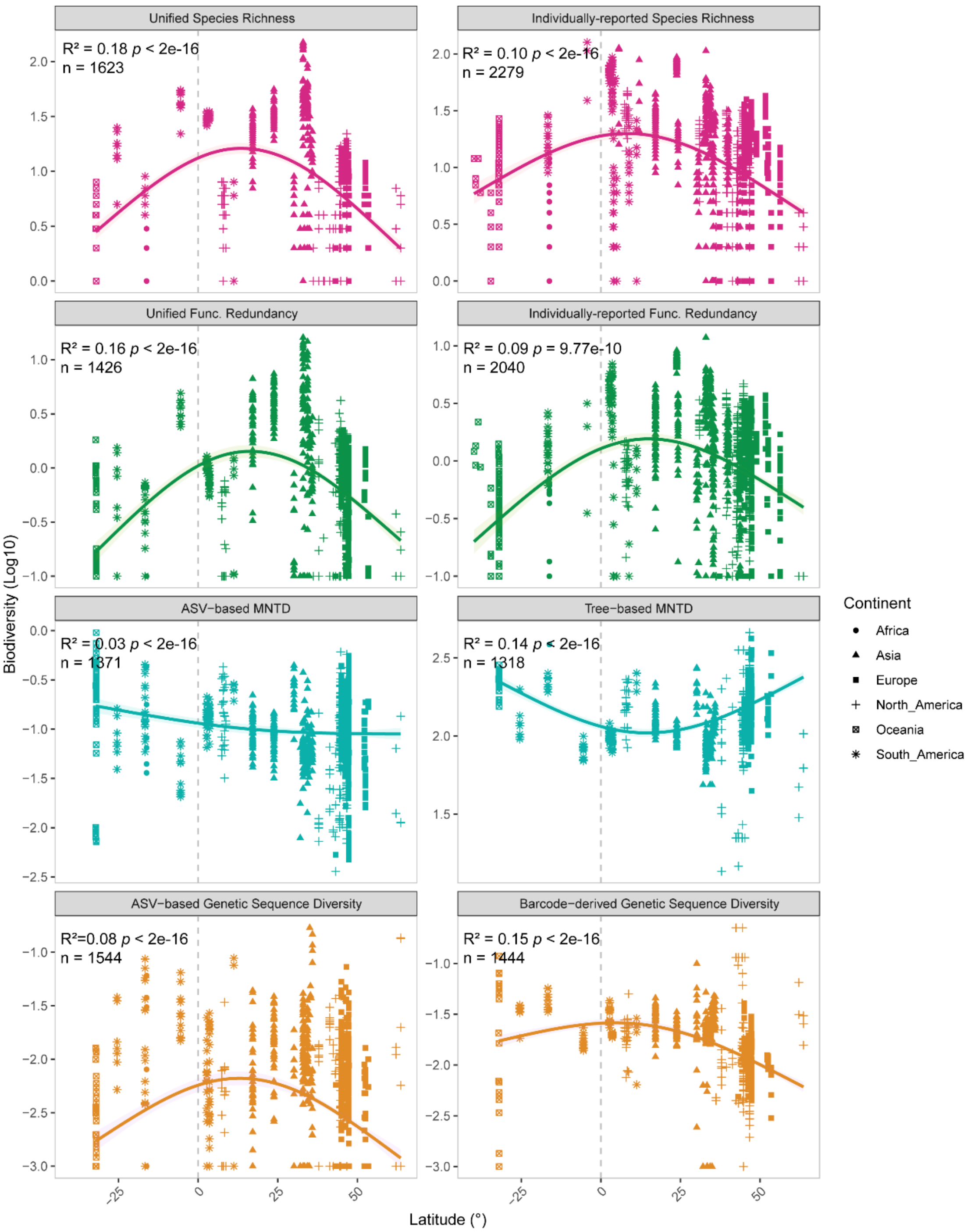

Supplementary Fig. 2 Latitudinal distribution of multi-faceted biodiversity. The regression lines were fitted using a generalized additive models with a smooth term (k=2). The shaded area represents the 95% confidence interval of the fit. *P* values are two-sided and test whether the corresponding regression coefficient differs from zero.

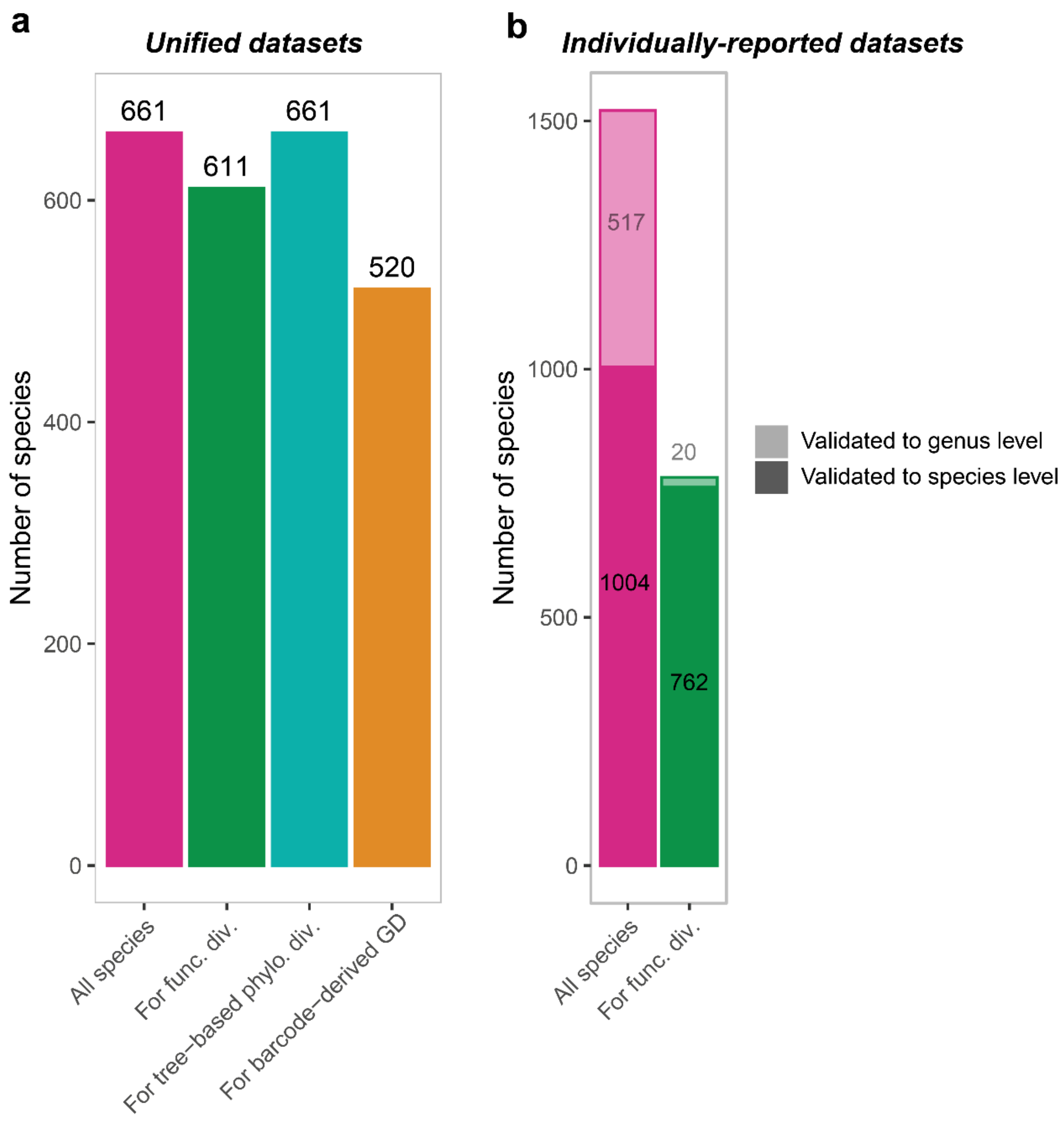

Supplementary Fig. 3 Summary of species included in the Unified and Original individually-reported datasets used to compute multi-faceted fish biodiversity.

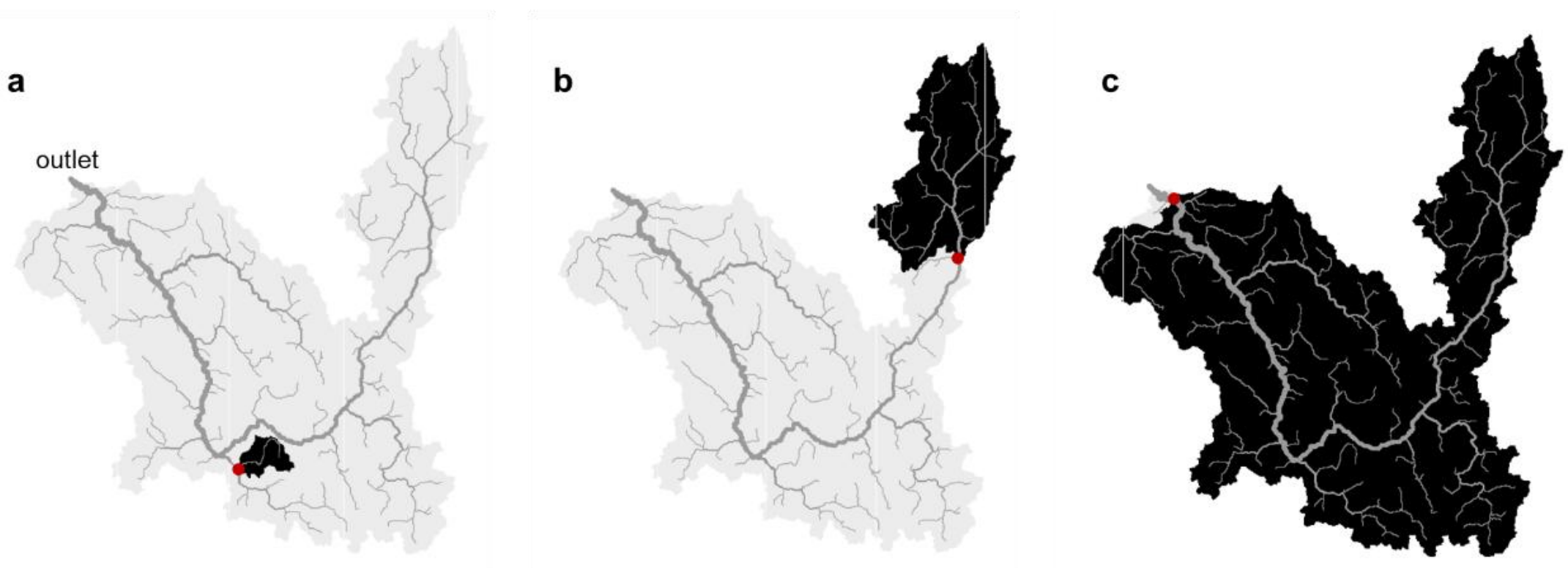

Supplementary Fig. 4 Example illustrating the delineation of sub-catchments for individual sampling sites.

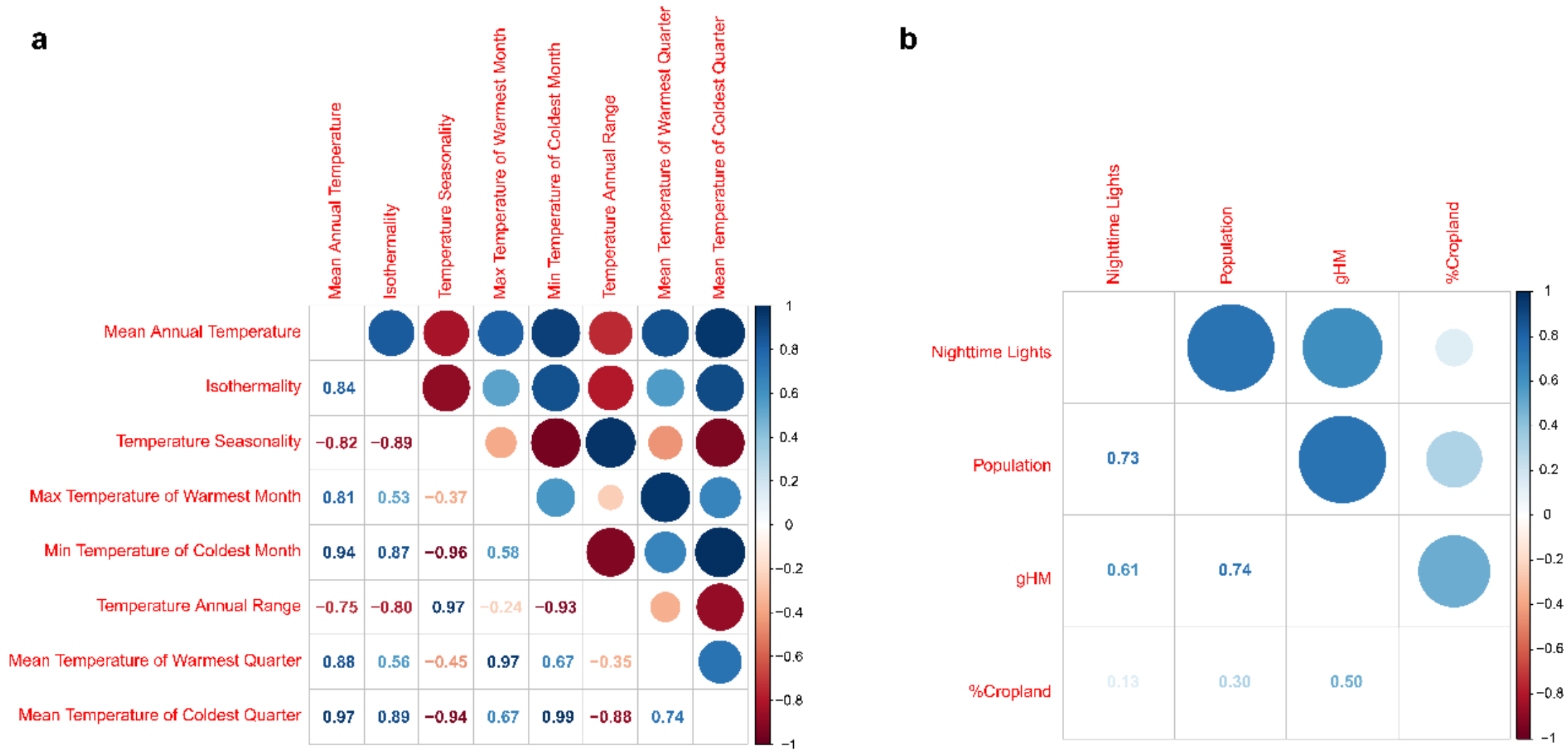

Supplementary Fig. 5 Correlation plots of climatic (a) and human-related (b) factors. Correlation analysis was performed using Pearson's correlation tests. The numbers and colors represent the correlation coefficients.

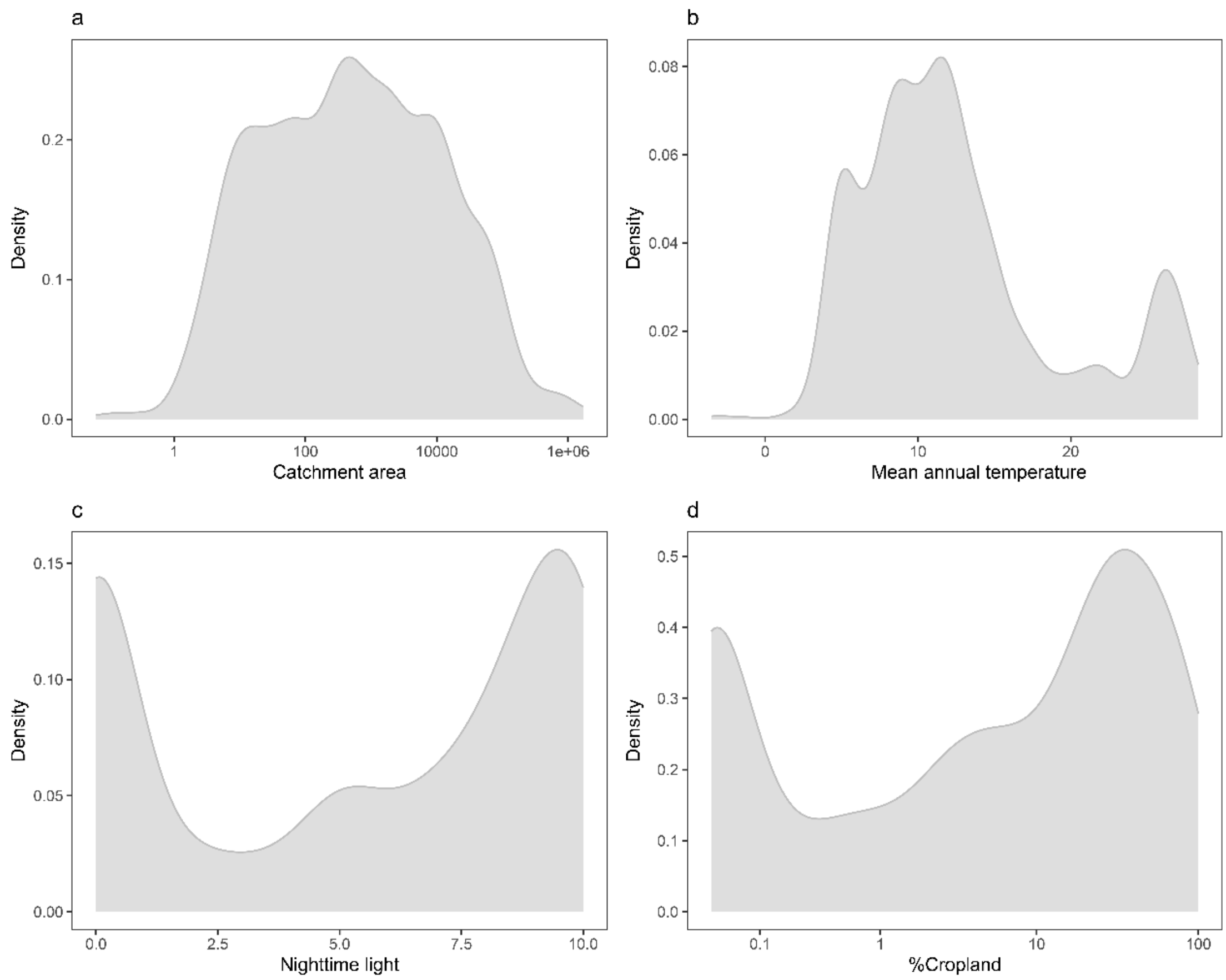

Supplementary Fig. 6 Density plots of drainage charateristics.

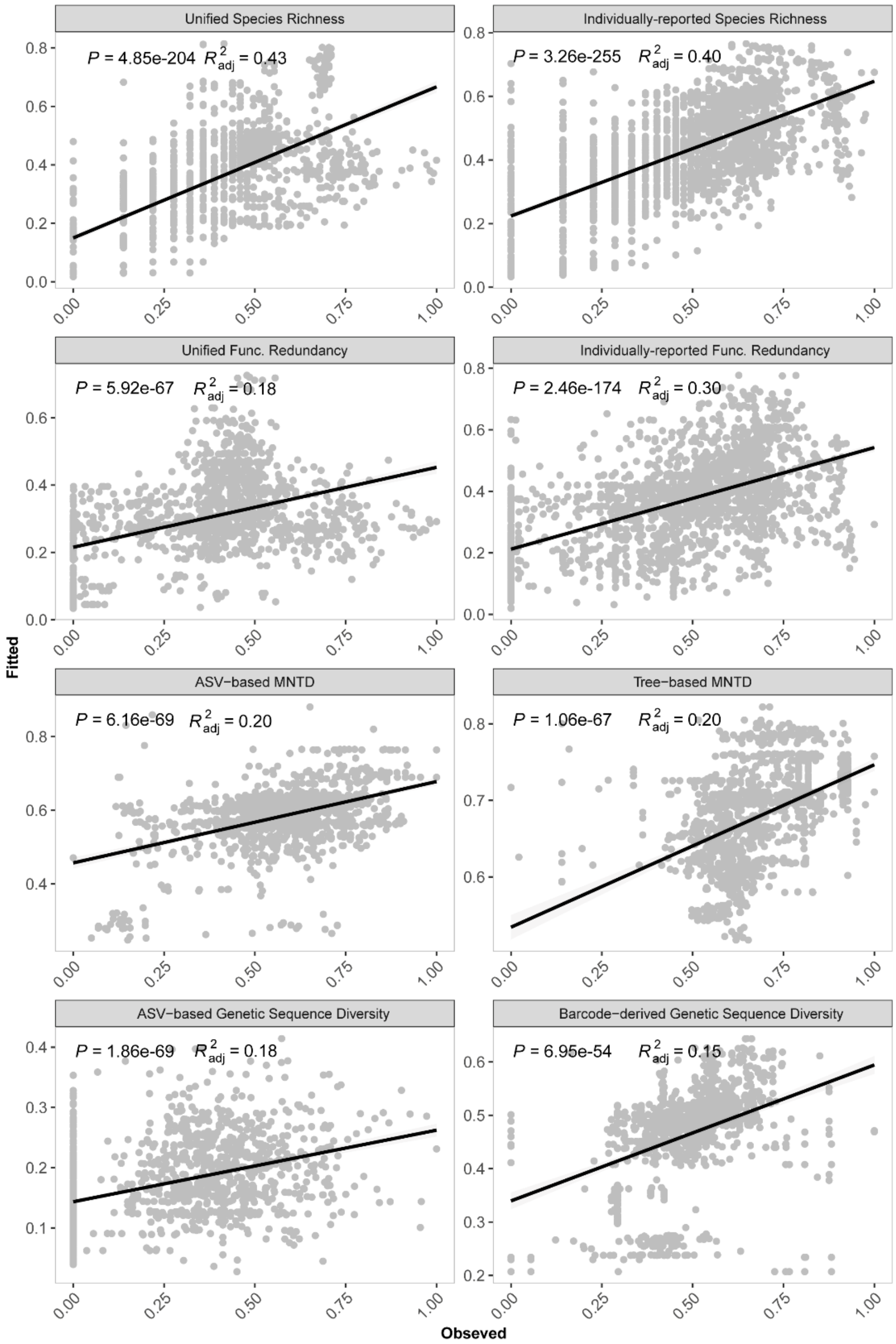

Supplementary Fig. 7 Fitted vs observed values of the full models. The regression lines were fitted using generalized linear models with a Gaussian error distribution. Reported *p* values are two-sided and test the null hypothesis that the slope coefficient equals zero. Adjusted R^2 is the standard coefficient of determination adjusted for the number of predictors. The shaded area represents the 95% confidence interval of the fit.

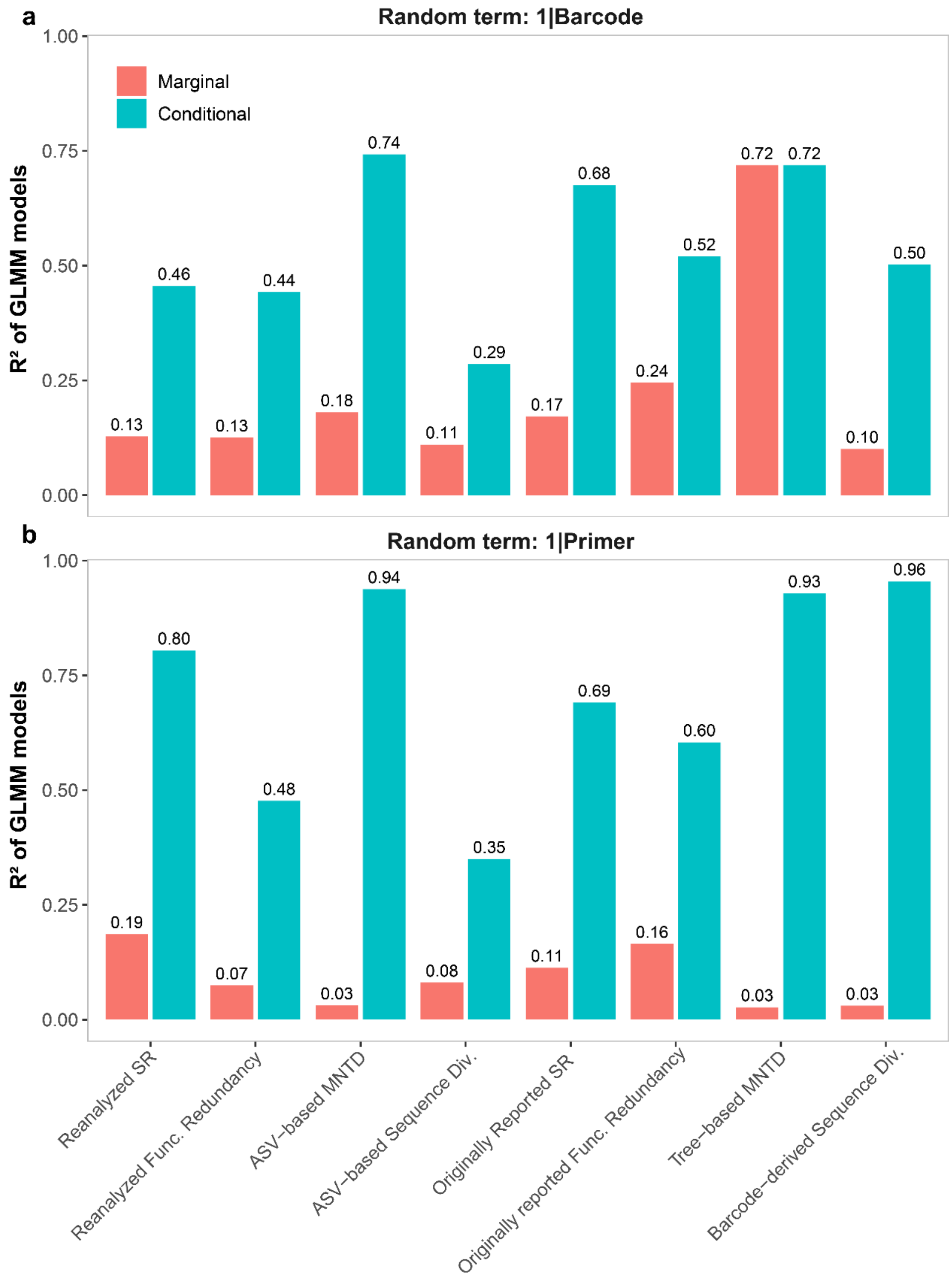

Supplementary Fig. 8 Marginal and conditional R^2 values for generalized linear mixed models fitted to each biodiversity metric. The model included catchment area, spatial autocorrelation, temperature, nighttime light intensity, and sample volume as fixed effects, with interactions between catchment area and environmental predictors. Barcode (a) or primer (b) was used as the random term.

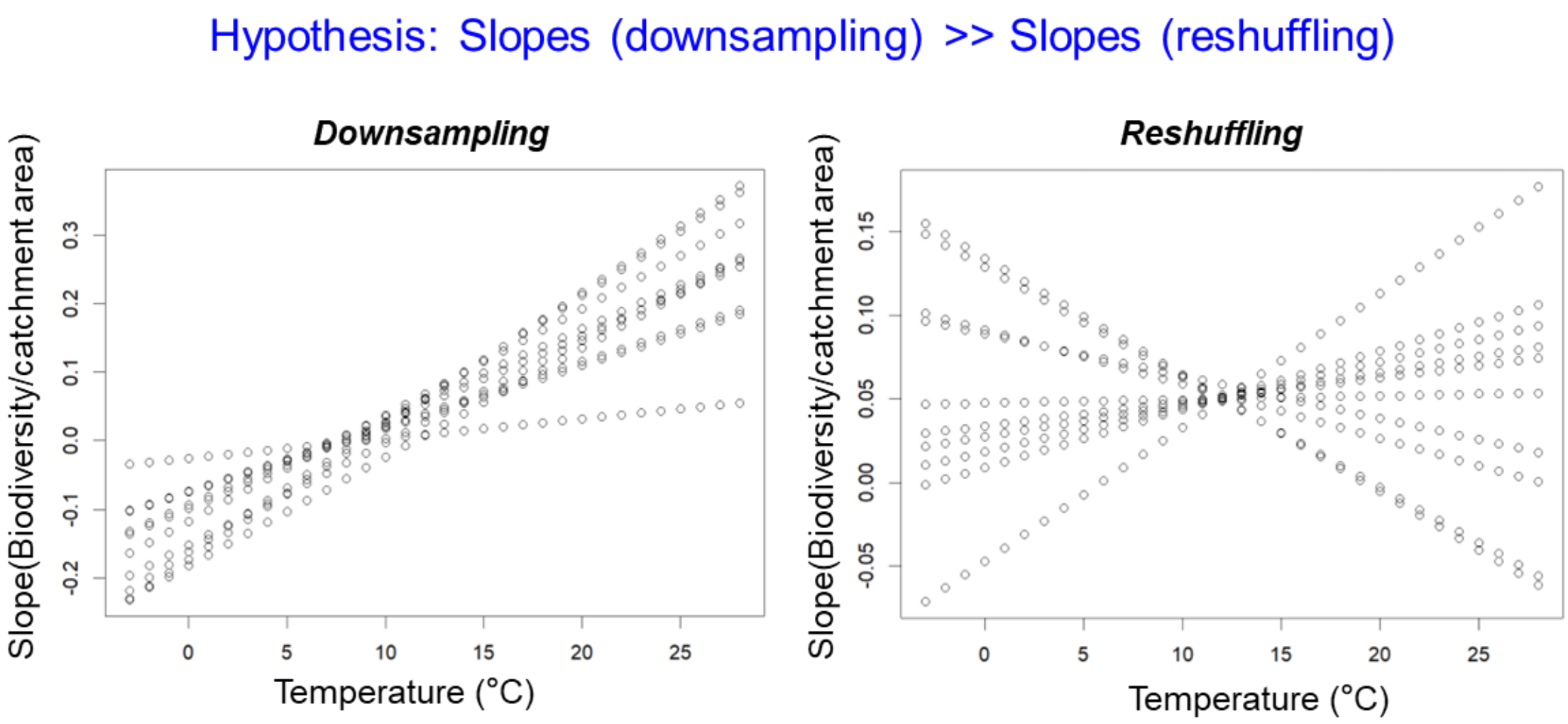

Supplementary Fig. 9 Examples showing predicted slopes derived from downsampling and reshuffling procedures.
