# Supplementary material for: A unified analysis of global riverine eDNA reveals common associations of fish biodiversity with drainage characteristics
Source: Nat Ecol Evol. 2026 Jun 24;10(10):1927–40. doi: 10.1038/s41559-026-03106-1 (PMC13658041; doi:10.1038/s41559-026-03106-1)
Supplement: Supplementary file 2 [file 41559_2026_3106_MOESM2_ESM.pdf]

nature portfolio

Corresponding author(s): Florian Altermatt and Xiaowei Zhang

Last updated by author(s): Feb 10, 2026

Reporting Summary

Nature Portfolio wishes to improve the reproducibility of the work that we publish. This form provides structure for consistency and transparency in reporting. For further information on Nature Portfolio policies, see our Editorial Policies and the Editorial Policy Checklist.

Statistics

For all statistical analyses, confirm that the following items are present in the figure legend, table legend, main text, or Methods section.

n/a	Confirmed	
☐	☒	The exact sample size (*n*) for each experimental group/condition, given as a discrete number and unit of measurement
☐	☒	A statement on whether measurements were taken from distinct samples or whether the same sample was measured repeatedly
☐	☒	The statistical test(s) used AND whether they are one- or two-sided<br>*Only common tests should be described solely by name; describe more complex techniques in the Methods section.*
☐	☒	A description of all covariates tested
☐	☒	A description of any assumptions or corrections, such as tests of normality and adjustment for multiple comparisons
☐	☒	A full description of the statistical parameters including central tendency (e.g. means) or other basic estimates (e.g. regression coefficient) AND variation (e.g. standard deviation) or associated estimates of uncertainty (e.g. confidence intervals)
☐	☒	For null hypothesis testing, the test statistic (e.g. *F*, *t*, *r*) with confidence intervals, effect sizes, degrees of freedom and *P* value noted<br>*Give P values as exact values whenever suitable.*
☒	☐	For Bayesian analysis, information on the choice of priors and Markov chain Monte Carlo settings
☒	☐	For hierarchical and complex designs, identification of the appropriate level for tests and full reporting of outcomes
☐	☒	Estimates of effect sizes (e.g. Cohen's *d*, Pearson's *r*), indicating how they were calculated

Our web collection on statistics for biologists contains articles on many of the points above.

Software and code

Policy information about availability of computer code

Data collection	No software was used to collect data.
Data analysis	Bioinformatics:<br>> VSEARCH, v2.7.1<br>> barcode_splitter<br>> OBITools, v1.01<br>> CUTADAPT, v4.6 with Python 3.10.13<br>> dada2, v1.29.0 in R<br>> Anacapa Toolkit<br><br>Data Analysis:<br>> R, v4.3.1

For manuscripts utilizing custom algorithms or software that are central to the research but not yet described in published literature, software must be made available to editors and reviewers. We strongly encourage code deposition in a community repository (e.g. GitHub). See the Nature Portfolio guidelines for submitting code & software for further information.

Data

Policy information about availability of data

All manuscripts must include a data availability statement. This statement should provide the following information, where applicable:

- Accession codes, unique identifiers, or web links for publicly available datasets
- A description of any restrictions on data availability
- For clinical datasets or third party data, please ensure that the statement adheres to our policy

The unified and standardized dataset of global riverine fish eDNA meta-analysis has been deposited in Dryad at https://doi.org/10.5061/dryad.gf1vhhn2h86. The minimum dataset required to interpret, verify and extend this study are available via the GitHub repository at https://github.com/Laura61616/GRiversFisheDNA_Main. Source data for Figures 1–5 and Extended Data Figures 1–5 are provided.

Research involving human participants, their data, or biological material

Policy information about studies with human participants or human data. See also policy information about sex, gender (identity/presentation), and sexual orientation and race, ethnicity and racism.

Reporting on sex and gender	Not applicable, as this study focuses on riverine fishes and does not involve the collection or analysis of human data.
Reporting on race, ethnicity, or other socially relevant groupings	Not applicable, as this study focuses on riverine fishes and does not involve the collection or analysis of human data.
Population characteristics	Not applicable, as this study focuses on riverine fishes and does not involve the collection or analysis of human data.
Recruitment	Not applicable, as this study focuses on riverine fishes and does not involve the collection or analysis of human data.
Ethics oversight	Not applicable, as this study focuses on riverine fishes and does not involve the collection or analysis of human data.

Note that full information on the approval of the study protocol must also be provided in the manuscript.

Field-specific reporting

Please select the one below that is the best fit for your research. If you are not sure, read the appropriate sections before making your selection.

☐ Life sciences ☐ Behavioural & social sciences ☒ Ecological, evolutionary & environmental sciences

For a reference copy of the document with all sections, see nature.com/documents/nr-reporting-summary-flat.pdf

Ecological, evolutionary & environmental sciences study design

All studies must disclose on these points even when the disclosure is negative.

Study description	Meta-analysis, global-scale study with no experimental treatments. Assessed multi-faceted riverine fish biodiversity using environmental DNA (eDNA) data collected from 1,818 sites across 113 river catchments on six continents. This study aimed to evaluate whether eDNA captures globally fish diversity patterns across taxonomic, functional, phylogenetic, and genetic dimensions. Relationships between biodiversity and natural (climatic, drainage area) and human-related drivers were analyzed to assess biodiversity–area scaling and sensitivity to human activities across upstream–downstream gradients.
Research sample	This is a meta-analysis of fish riverine eDNA and thus no samples were collected in this study.
Sampling strategy	This is a meta-analysis of fish riverine eDNA and thus no samples were collected in this study.
Data collection	A comprehensive literature search was conducted on 13 December 2022 using the Web of Science Core Collection to identify studies employing eDNA metabarcoding for assessing fish communities in riverine ecosystems. The search returned 673 records and only relevant original research was retained, yielding 50 studies. Further screening for data completeness (geographic coordinates and species richness or raw data availability) reduced this to 42 studies. To complement the published data, an open call for additional datasets was issued via X (formerly Twitter), leading to 23 new datasets from co-authors and collaborators. After removing overlapping and single-site studies, 58 datasets were retained for meta-analysis. For each dataset, raw sequencing data, site-level species richness, and species-by-site matrices were compiled.
Timing and spatial scale	We compiled a global fish eDNA dataset integrating published and unpublished studies from 113 river catchments (1,818 sites) across six continents, spanning 2012–2023. The literature search was completed on 13 December 2022; thus, studies published thereafter were not included.
Data exclusions	No data were excluded from the analysis. Both the raw and unified datasets have been deposited in Dryad under the DOI 10.5061/

Data exclusions	dryad.gf1vhhn2h.
Reproducibility	Each eDNA study typically included one to ten biological replicates per site. Replicates were merged within sites to ensure comparability across datasets. To assess model robustness, we applied both downsampling and reshuffling approach. Full models were repeatedly fitted to random subsamples comprising 70% of sites, and slope estimates were recalculated 1,000 times. To determine whether biodiversity–area scaling reflected genuine ecological patterns, temperature and nighttime light intensity were randomly reassigned among sites in 1,000 reshuffling iterations. p values were then calculated as the proportion of reshuffled slopes exceeding the median from the downsampling analysis.
Randomization	The compiled dataset comprised 1,818 sampling sites from 113 river catchments across six continents, originating from 58 independently conducted eDNA studies. Sampling volume and primer/barcode identity were treated as random effects in all statistical models to account for methodological heterogeneity and to isolate genuine ecological patterns from study-specific biases.
Blinding	Blinding was not applicable to this study. Data compilation was based on independently conducted eDNA studies from global river systems, where sampling and analysis were performed prior to this meta-analysis.

Did the study involve field work? ☐ Yes ☒ No

Reporting for specific materials, systems and methods

We require information from authors about some types of materials, experimental systems and methods used in many studies. Here, indicate whether each material, system or method listed is relevant to your study. If you are not sure if a list item applies to your research, read the appropriate section before selecting a response.

Materials & experimental systems

n/a	Involved in the study	
☒	☐	Antibodies
☒	☐	Eukaryotic cell lines
☒	☐	Palaeontology and archaeology
☒	☐	Animals and other organisms
☒	☐	Clinical data
☒	☐	Dual use research of concern
☒	☐	Plants

Methods

n/a	Involved in the study	
☒	☐	ChIP-seq
☒	☐	Flow cytometry
☒	☐	MRI-based neuroimaging

Plants

Seed stocks	Not applicable, as no plant materials used in this study.
Novel plant genotypes	Not applicable, as no plant materials used in this study.
Authentication	Not applicable, as no plant materials used in this study.
