# Supplementary material for: A unified analysis of global riverine eDNA reveals common associations of fish biodiversity with drainage characteristics
Source: Nat Ecol Evol. 2026 Jun 24;10(10):1927–40. doi: 10.1038/s41559-026-03106-1 (PMC13658041; doi:10.1038/s41559-026-03106-1)
Supplement: Supplementary file 3 [file 41559_2026_3106_MOESM3_ESM.pdf]

nature portfolio

Peer Review File

A unified analysis of global riverine eDNA reveals common associations of fish biodiversity with drainage characteristics

Corresponding Author: Professor Florian Altermatt

Version 0:

Decision Letter:

11th December 2025

*Please ensure you delete the link to your author homepage in this e-mail if you wish to forward it to your co-authors.

Dear Professor Altermatt,

Your manuscript entitled "Globally unified analysis of riverine eDNA reveals common associations of fish biodiversity with drainage characteristics" has now been seen by 3 reviewers, whose comments are attached. The reviewers have raised a number of concerns which will need to be addressed before we can offer publication in Nature Ecology & Evolution. We will therefore need to see your responses to the criticisms raised and to some editorial concerns, along with a revised manuscript, before we can reach a final decision regarding publication.

We therefore invite you to revise your manuscript taking into account all reviewer and editor comments. Please highlight all changes in the manuscript text file in Microsoft Word format.

We are committed to providing a fair and constructive peer-review process. Do not hesitate to contact us if there are specific requests from the reviewers that you believe are technically impossible or unlikely to yield a meaningful outcome.

When revising your manuscript:

* Include a “Response to reviewers” document detailing, point-by-point, how you addressed each reviewer comment. If no action was taken to address a point, you must provide a compelling argument. This response will be sent back to the reviewers along with the revised manuscript.

* If you have not done so already please begin to revise your manuscript so that it conforms to our Article format instructions at http://www.nature.com/natecolevol/info/final-submission. Refer also to any guidelines provided in this letter.

* Include a revised version of any required reporting checklist. It will be available to referees (and, potentially, statisticians) to aid in their evaluation if the manuscript goes back for peer review. A revised checklist is essential for re-review of the paper.

* Extended Data Figures - please ensure that any supplementary figures and tables that are crucial to the manuscript’s conclusions are converted into Extended Data figures and tables to increase visibility of these data. Extended Data figures and tables are online-only (present in the online PDF and full-text HTML versions of the paper), peer-reviewed display items that provide essential background to the article but are not included in the main article due to space constraints. A maximum of ten Extended Data display items (figures and tables) is permitted.

Please use the link below to submit your revised manuscript and related files:

Link Redacted

Note: This URL links to your confidential home page and associated information about manuscripts you may have submitted, or that you are reviewing for us. If you wish to forward this email to co-authors, please delete the link to your homepage.

We hope to receive your revised manuscript within four to eight weeks. If you cannot send it within this time, please let us

know. We will be happy to consider your revision so long as nothing similar has been accepted for publication at Nature Ecology & Evolution or published elsewhere.

Nature Ecology & Evolution is committed to improving transparency in authorship. As part of our efforts in this direction, we are now requesting that all authors identified as ‘corresponding author’ on published papers create and link their Open Researcher and Contributor Identifier (ORCID) with their account on the Manuscript Tracking System (MTS), prior to acceptance. ORCID helps the scientific community achieve unambiguous attribution of all scholarly contributions. You can create and link your ORCID from the home page of the MTS by clicking on ‘Modify my Springer Nature account’. For more information please visit please visit www.springernature.com/orcid.

Please do not hesitate to contact me if you have any questions or would like to discuss these revisions further.

We look forward to seeing the revised manuscript and thank you for the opportunity to review your work.

[redacted]

Reviewer expertise:

Reviewer #1: eDNA, molecular ecology

Reviewer #2: eDNA, freshwater ecology

Reviewer #3: eDNA, microbial freshwater ecology

Reviewers' comments:

Reviewer #1 (Remarks to the Author):

Zhang et al. here have delivered an analysis that I have been wanting to see/do for quite some years, coinciding with a respectable number of co-analysed eDNA fish datasets to leverage information about global fish biodiversity and environmental factors from combined eDNA datasets.

It is a valuable contribution to the literature and where more studies should be going. I am only sorry that some newer outputs, post the 2022 haul, were not included, but there will be room for additions in the future. It is a reanalysis of data, but I feel an important proof of principle to demonstrate what is possible to achieve from the growing number of eDNA studies.

My first question was ‘how different is the eDNA study to what is already known?’ but in essence, they needed the companion data to assess how well the combined eDNA analyses worked in relation to legacy existing datasets, which provides a good validation to their new analysis. It is actually a methods paper, but one that brings additional information about the status of biodiversity to the table and I congratulate the authors on the thorough job that they have performed in acquiring diverse information sets, and creative indexes from the data, notwithstanding good visuals and graphical figures full of information. Because this is a fish dataset, I was also wondering if it was just one marker, but not; it is a combined analysis of multiple markers, with statistical considerations for markers/regions, a job that with fish data should be achievable, especially wrt to the maturity of fish reference databases.

Digging deeper?
I will collate the usual finer comments below, but also ask whether or not it could be possible to derive further information from the data? There is discussion about for example the loss of lineages and increasing MNTD distances and the results and discussion, but it would be interesting for the reader interested in the ecology of the systems to know about specific examples whereby particular lines have been lost? What are these species and what sort of functions did they perform in the river ecosystems? Further, I like the use of luminescence as a proxy for the human population and follow the narrative about the negative impacts of human activities, but can this be broken down even further? Is it population number, which would indicate (organic, inorganic) chemical pressure, or could it be human activity in the form of habitat modification, canalisation etc.. The team are clearly creative when it comes to deriving interesting metrics associated with the relationship between biodiversity and the environment and so I am curious to suggest that further information could be derived from the data set, with some additional investigations regarding the precise impacts on the ecosystem as a consequence of human presence.

Further comments:

Line 166 – Genetic diversity mentions adaptive potential but the markers generally used in metabarcoding studies have been chosen for their neutral mode of evolution and not adaptative potential and so this needs to be clear that they are using the mb data as a proxy for broader genetic capacity.

Line 244 – Can the team provide ecological information regarding the increase in MNTD at higher latitudes, which taxa are driving the results?

Line 236 – Markers are mentioned here perhaps for the first time in the methods – we need more information earlier about markers, loci, primers etc..

Line 345 – There is a good example of space and biodiversity in Dobson et al. 2022 - https://www.sciencedirect.com/science/article/pii/S0043135422007175 ; consider citing?
Line 359 – human activity – can they find more human activity metrics, population, habitat modification etc..?

Line 411 – what does this look like on the ground? There is a temptation to suggest that some of the results/discussion are too superficial and not doing the rich data the justice that it deserves?

Line 497 – what sort of compute power was used here please?

Line 549 – what markers were used. Here and elsewhere, what primers, markers were used?

Line 602 – mean annual temperature – is this a good measure? Could high and low temps be more informative?

Line 622 – Reference missing here?

Figure 2 – d, e and f show S, MNTD and sequence diversity, but what about functional redundancy? What is going on with the vs. text with lightning bolts emerging from the text?

Line 944 – Is the very large spread on figures e and f, MNTD and sequence diversity derived from the rough proxy of all available sequences >600 bp for the comparision with existing data, ie not geolocated – could they be comparing unrelated things here and is enough mentioned in the text to acknowledge the potential limitations? How many sequences were included per species?

Reviewer #1 (Remarks on code availability):

NA

Reviewer #2 (Remarks to the Author):

Overall, the manuscript is clearly written and the analytical framework is generally sound. The conclusions are well aligned with the results and are presented with appropriate nuance. While I was not able to examine every aspect of the manuscript in exhaustive detail, I found the study to be well structured and thoughtfully interpreted. I do, however, highlight two methodological points that would benefit from clarification or further justification:

Genetic diversity estimates across markers:
The authors derive estimates of genetic diversity from different genetic regions (COI, 12S, and 16S). Because these loci evolve under distinct evolutionary models and rates, combining or comparing them requires an explicit evolutionary framework that accounts for marker-specific substitution processes. It is not clear from the current manuscript how these differences were handled, and additional explanation or justification would strengthen the robustness of the genetic diversity inferences.

Use of fishmorph traits:
The traits extracted from the FishMorph database are primarily morphometric shape descriptors. While these may carry potential functional implications, referring to overlaps or differences derived from these variables as “functional” may overstate their interpretability. I suggest clarifying the distinction between morphological and functional traits and moderating claims about functional differentiation unless supported by additional evidence.

Overall, this is a valuable and thought-provoking study with considerable potential, and I encourage the authors to address the points above to further enhance clarity and rigor.

Reviewer #3 (Remarks to the Author):

This manuscript by Zhang et al. presents a globally harmonised synthesis of fish eDNA metabarcoding data from >1,800 sites across 113 river catchments worldwide. The authors reprocess publicly available raw sequence data to generate a unified ASV dataset and calculate taxonomic, functional, phylogenetic and genetic biodiversity metrics. They then model how these facets vary along river networks and interact with climate and human pressure. The work is ambitious, timely, and highly relevant to global biodiversity monitoring frameworks.

This is a good paper with significant conceptual and methodological contributions. The global integration of eDNA datasets is novel and valuable, and the multi-facet biodiversity framework is particularly compelling.
However, there are some areas where the manuscript could be improved, particularly regarding conceptual clarity, covariate interpretation, and the robustness of genetic diversity metrics, which require further refinement before publication. The manuscript would benefit from clearer articulation of mechanistic explanations and more detailed sensitivity analyses, especially regarding methodology biases and spatial sampling heterogeneity. The manuscript is innovative and promising,

but could be strengthened by improving methodological robustness and clarifying mechanistic interpretations.

Comments
The genetic diversity derived from short eDNA amplicons is interesting but methodologically limited. The authors acknowledge this, but more detail is needed on how much between- and within-species sequence variation is captured by short amplicon markers, and on the potential confounding effects of multiple species being grouped under imperfect taxonomic assignment. It would also be helpful to understand the geographic biases in COI sampling used for validation. I suggest including a sensitivity analysis illustrating how genetic diversity varies with (a) taxonomic assignment confidence, (b) read depth, and (c) marker type (12S vs 16S).

Although a unified ASV pipeline improves comparability, large differences remain across studies in sample volume, filtration, extraction, PCR cycles, primer pairs and sequencing platforms. Primer-specific random effects are helpful but may not capture systematic lineage-specific bias. It would be useful to provide a summary table quantifying the distribution of methodological variables across studies and, if possible, to add a supplementary model that explicitly tests sensitivity to filtration volume, primer set, lab of origin, and sequencing platform. This will help reassure readers that macroecological results are robust to protocol variation.

Night-time lights correlate with urbanisation but not with other stressors such as agriculture, hydropower, nutrient or chemical pollution, or water flows or abstraction. Many catchments in the dataset are likely influenced by impacts not described by artificial lights. It may be worth adding at least one additional proxy (e.g. % cropland, population density, environmental flow modification index). If this is not possible, provide a stronger justification for relying solely on night-time light.

The key finding is that richness, functional redundancy, and genetic diversity respond differently from phylogenetic diversity. However, the mechanisms are discussed mostly qualitatively. Consider expanding the discussion to clarify why phylogenetic diversity shows opposite area–human activity interactions, which specific lineages drive changes in MNTD, and whether homogenisation signals reflect loss of ancient lineages or proliferation of recent, widespread taxa.

Minor Comments
1. Clarify whether COI barcode sequences from GenBank were filtered for quality and geographic relevance.
2. Consider discussing the potential influence of invasive fish species on patterns of functional and genetic diversity.
3. Provide effect sizes in addition to significance for all main models, preferably in a standardised format.
4. Provide details on how pseudo-replicated sites (multiple samples at the same coordinates) were handled.
5. Expand the explanation for why species richness correlates more strongly with historical data in the unified dataset compared to the original datasets.
6. Clarify whether rarefaction was applied to reads before ASV calling.

********************END********************

Version 1:

Decision Letter:

18th March 2026

Dear Dr. Altermatt,

Thank you for submitting your revised manuscript "Globally unified analysis of riverine eDNA reveals common associations of fish biodiversity with drainage characteristics" (NATECOLEVOL-25093271A). It has now been seen again by the original reviewers and their comments are below. The reviewers find that the paper has improved in revision, and therefore we'll be happy in principle to publish it in Nature Ecology & Evolution, pending minor revisions to satisfy the reviewers' final requests and to comply with our editorial and formatting guidelines.

If the current version of your manuscript is in a PDF format, please email us a copy of the file in an editable format (Microsoft Word or LaTex)-- we can not proceed with PDFs at this stage.

If you have not done so already, please ensure that you also email us a completed copy of the Reporting summary :

Reporting summary: https://www.nature.com/documents/nr-reporting-summary.pdf

We are now performing detailed checks on your paper and will send you a checklist detailing our editorial and formatting requirements in about a week. Please do not upload the final materials and make any revisions until you receive this additional information from us.

Thank you again for your interest in Nature Ecology & Evolution. Please do not hesitate to contact me if you have any questions.

[redacted]

Reviewer #1 (Remarks to the Author):

Zhang et al. here have addressed my original comments with enthusiasm and consequently (also suggested by Reviewer #3) have also discovered more about the anthropogenic stressors that could be related to the fish biodiversity in their study. I hope that the manuscipt has been strengthened and I can see that they have attended to the comments robustly from the remaining reviewers. The work provides an excellent example of what large scale data synthesis can bring to ecological understanding from the eDNA world and I hope to see the manuscript published in the near future.
Best wishes, Si Creer

Reviewer #1 (Remarks on code availability):

NA

Reviewer #2 (Remarks to the Author):

I feel the authors have provided an appropriate and satisfactory response to my concerns and have no further comments.

Reviewer #3 (Remarks to the Author):

I would like to thank the authors for their detailed and thorough responses to my comments (and those of the other reviewers) - I'm happy that these have been addressed to my satisfaction.

Open Access This Peer Review File is licensed under a Creative Commons Attribution 4.0 International License, which permits use, sharing, adaptation, distribution and reproduction in any medium or format, as long as you give appropriate credit to the original author(s) and the source, provide a link to the Creative Commons license, and indicate if changes were made.
In cases where reviewers are anonymous, credit should be given to 'Anonymous Referee' and the source.
The images or other third party material in this Peer Review File are included in the article's Creative Commons license, unless indicated otherwise in a credit line to the material. If material is not included in the article's Creative Commons license and your intended use is not permitted by statutory regulation or exceeds the permitted use, you will need to obtain permission directly from the copyright holder.
To view a copy of this license, visit https://creativecommons.org/licenses/by/4.0/

Reviewers' comments:

Reviewer #1 (Remarks to the Author):

Comment #1: Zhang et al. here have delivered an analysis that I have been wanting to see/do for quite some years, coinciding with a respectable number of co-analysed eDNA fish datasets to leverage information about global fish biodiversity and environmental factors from combined eDNA datasets.

RESPONSE: We sincerely thank the reviewer for this encouraging feedback. We are pleased that our work aligns with a long-standing open research question and contributes to leveraging the growing body of eDNA datasets to understand global fish biodiversity patterns. We have been driven by the same motivation that you point out: the increasing availability of individual eDNA fish biodiversity dataset called for an integration, and we are happy to see it is reflecting your interest.

Comment #2: It is a valuable contribution to the literature and where more studies should be going. I am only sorry that some newer outputs, post the 2022 haul, were not included, but there will be room for additions in the future. It is a reanalysis of data, but I feel an important proof of principle to demonstrate what is possible to achieve from the growing number of eDNA studies.

RESPONSE: We thank the reviewer for this positive and encouraging assessment. For any meta-analysis, the decision on the cutoff (date-wise) of studies to include is indeed critical. We had done an extensive search on both published and unpublished dataset., and a cutoff by early 2023 for practical reasons: we needed sufficient time and carefulness to align and re-analyze the datasets (contrastingly to classical meta-analyses that rely mostly on compound values already, we re-analyzed all raw data). We fully agree that the continued expansion of eDNA studies, including those published from 2023 onwards, will offers substantial further opportunities for future extensions of this work. As the aim of our study was not to provide an exhaustive synthesis of all available fish eDNA datasets, but rather to demonstrate the capacity of existing eDNA data to reveal large-scale patterns of global riverine fish diversity, the number of studies included and the cut-off value are less central. By reanalyzing a broad collection of datasets, our intention was to provide a fundamental demonstration illustrating what is already achievable through the integration of fish eDNA studies across regions.

Comment #3: My first question was ‘how different is the eDNA study to what is already known?’ but in essence, they needed the companion data to assess how well the combined eDNA analyses worked in relation to legacy existing datasets, which provides a good validation to their new analysis. It is actually a methods paper, but one

that brings additional information about the status of biodiversity to the table and I congratulate the authors on the thorough job that they have performed in acquiring diverse information sets, and creative indexes from the data, notwithstanding good visuals and graphical figures full of information. Because this is a fish dataset, I was also wondering if it was just one marker, but not; it is a combined analysis of multiple markers, with statistical considerations for markers/regions, a job that with fish data should be achievable, especially wrt to the maturity of fish reference databases.

RESPONSE: Thank you. We are glad that the aims, methodological focus, and contribution of the study, particularly the validation against legacy datasets and the integration of multiple genetic markers, are clearly conveyed.

We appreciate the reviewer's assessment of the work and their highlighting that it is a combination of methodological advances that is also providing additional insight into large-scale patterns of freshwater fish biodiversity. Specific points raised by the reviewer are addressed in detail below, together with the corresponding revisions made to the manuscript.

Comment #4: Digging deeper?

I will collate the usual finer comments below, but also ask whether or not it could be possible to derive further information from the data? There is discussion about for example the loss of lineages and increasing MNTD distances and the results and discussion, but it would be interesting for the reader interested in the ecology of the systems to know about specific examples whereby particular lines have been lost? What are these species and what sort of functions did they perform in the river ecosystems? Further, I like the use of luminescence as a proxy for the human population and follow the narrative about the negative impacts of human activities, but can this be broken down even further? Is it population number, which would indicate (organic, inorganic) chemical pressure, or could it be human activity in the form of habitat modification, canalisation etc.. The team are clearly creative when it comes to deriving interesting metrics associated with the relationship between biodiversity and the environment and so I am curious to suggest that further information could be derived from the data set, with some additional investigations regarding the precise impacts on the ecosystem as a consequence of human presence.

RESPONSE: Thank you for these thoughtful suggestions and for encouraging a deeper ecological interpretation of the results. We agree that linking the observed phylogenetic patterns to specific taxa, lineages, and human pressures is of broad ecological interest. We now specifically expanded on adding/including further information derived from the data:

(1) Taxa/lineage driving phylogenetic patterns

We have conducted additional analyses to better understand the driving lineages of the observed MNTD patterns. Specifically, we assessed changes in site-level MNTD following the sequential removal of individual family, allowing us to identify those taxa that contribute most strongly to the observed shifts in phylogenetic structure. Across all 115 families, the correlation coefficient (Pearson's r) between baseline MNTD (no family removed) and drop-one-family MNTD ranged from 0.95 to 1.00, and we observed no meaningful changes in MNTD estimates attributable to the removal of any single family. Together, these results suggest that the phylogenetic signal is more likely to reflect lineage turnover distributed across many parts of the tree, rather than being driven by a small set of ecologically distinctive lineages.

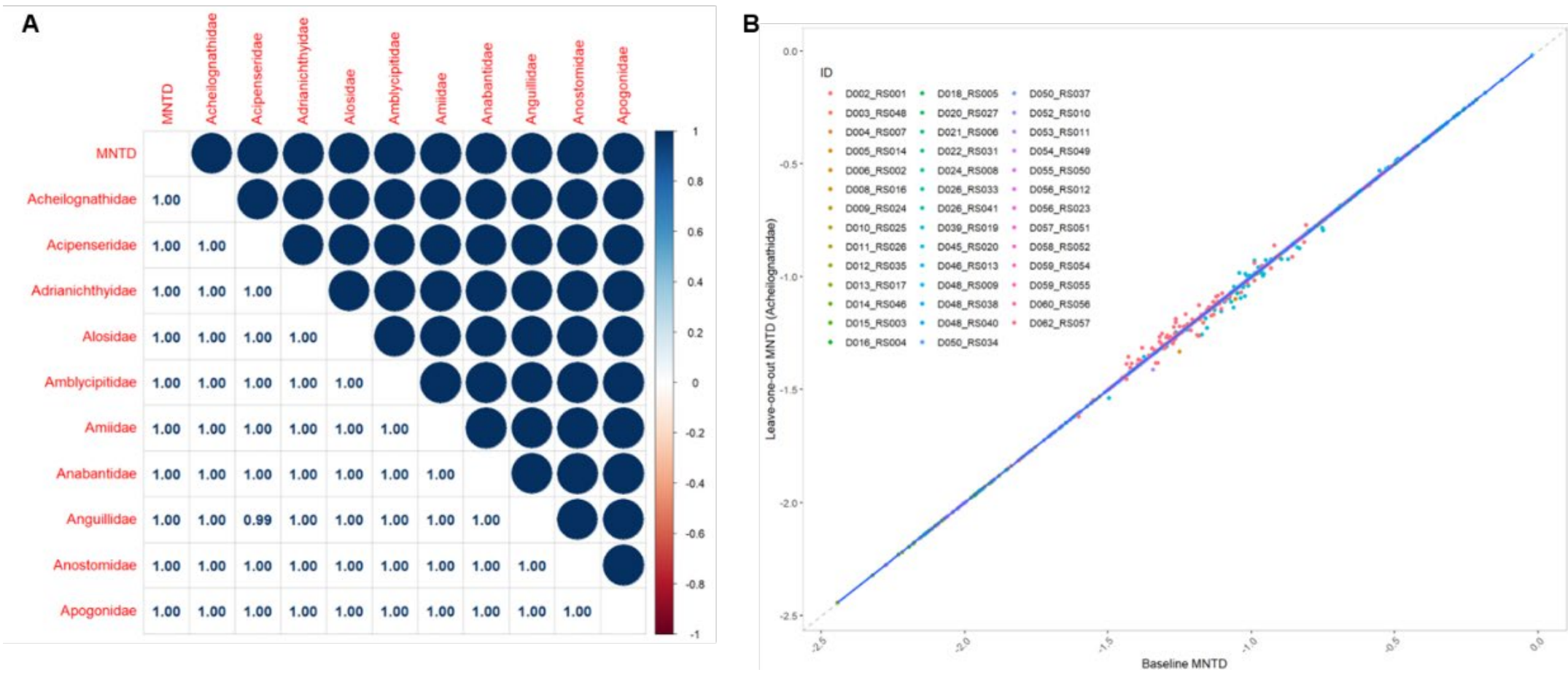

Figure legend: Correlation plot of drop-one-family MNTD, where "MNTD" denotes the baseline value with no family removed; only ten families are shown. Across all 115 families, the correlation coefficient (r) between baseline and drop-one-family MNTD ranges from 0.95 to 1.00.

(2) More human activity metrics

We agree that disentangling different components of human activities is an important challenge and based on your comment we have now tested and integrated further dimensions describing (different) anthropogenic activities. Initially, we had used nighttime lights as a variable strongly associated with urbanization. In our revisions, we have now considered/tested several further globally available human-related variables. Specifically, these are the human population density, the percentage cropland cover (%cropland) in the catchment, and the Global Human Modification (gHM) index. These variables capturing various components of anthropogenic activities, including both urbanization and agricultural land-use changes. In a first step we did correlation-analyses between all four of these anthropogenic variables, finding

that Population density and gHM were highly correlated with nighttime lights (with correlation coefficients of 0.61 and 0.73, respectively), whereas %cropland showed weaker associations (correlation coefficient of 0.13) (see figure below, also given in the supplement). Based on this, and to avoid overfitting of the model, we kept two human-related variables: nighttime lights as an urbanization proxy and %cropland as an agricultural proxy.

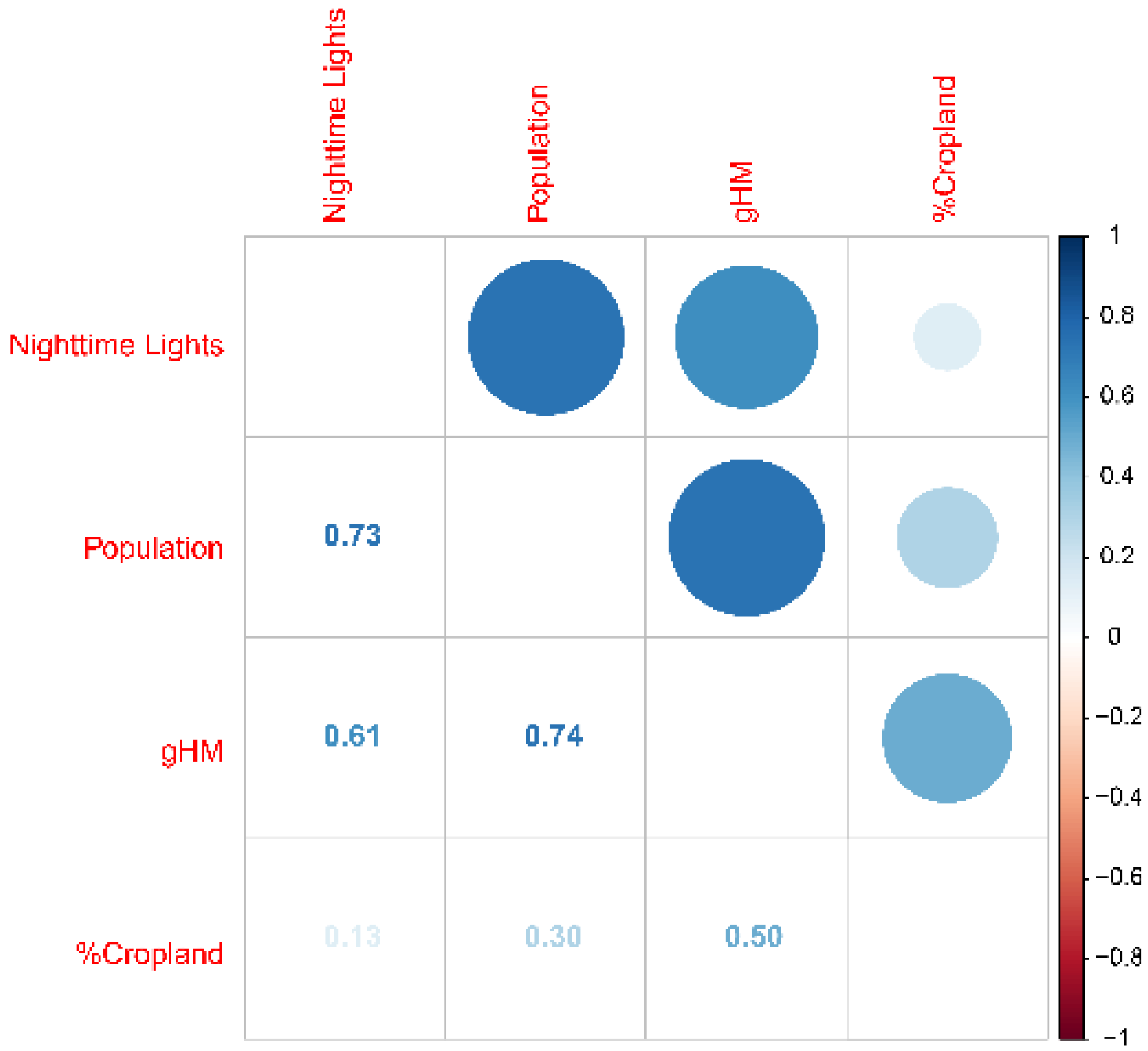

Supplementary Figure 5b Correlation plots of human activity factors.

We then re-fitted the model including both nighttime lights and %cropland as human activity proxies and used likelihood ratio tests to evaluate whether the main effects and interaction effects (with catchment area) of %cropland were significant across the eight biodiversity facets. This additional analysis not only corroborated the effect of night-time light (as a proxy capturing population density/urbanization), but indeed also showed that the main effect of %cropland was significant for all biodiversity metrics except individually reported species richness, whereas the interaction with cropland cover was generally non-significant (except for ASV-based sequence diversity and unified functional redundancy; see Extended Data Table 2). Thus, adding an additional variable capturing the agricultural anthropogenic activities indeed

expanded the explanatory power. We are very thankful for your suggestion and are happy to not only corroborate the previous analyses, yet also expand to an additional important anthropogenic driver. We only included the main effect of %cropland cover, in the final model, as inclusion of an interaction with catchment area led to overparameterization (the directions of effects remained robust, yet attenuated the main patterns and the model not being statistically robust any more).

In the updated model, the results are highly robust relative to previous findings. The inclusion of this agricultural proxy supports the relevance of agriculture, alongside urbanization, as an anthropogenic driver. Importantly, the results were highly congruent both qualitatively and quantitatively with our previous analyses, corroborating the robustness of our approach.

Extended Data Table 2 (partial) Results of likelihood ratio tests assessing the significance of individual model terms.

Biodiversity metrics	Test term	Random ter	df	AIC	BIC	logLik	deviance	Chisq	p value
Unified species richness	%cropland	Primer	12	-2797.03	-2732.33	1410.52	-2821.03	29.71	<0.001
Unified species richness	catchment_area:%cropland	Primer	13	-2798.04	-2727.95	1412.02	-2824.04	3.01	0.083
Unified func. redundancy	%cropland	Primer	12	-2759.81	-2696.66	1391.91	-2783.81	9.14	0.002
Unified func. redundancy	catchment_area:%cropland	Primer	13	-2776.35	-2707.94	1401.17	-2802.35	18.53	<0.001
ASV-based MNTD	%cropland	Barcode	12	-1242.94	-1180.26	633.47	-1266.94	19.79	<0.001
ASV-based MNTD	catchment_area:%cropland	Barcode	13	-1241.15	-1173.25	633.58	-1267.15	0.22	0.642
ASV-based sequence diversity	%cropland	Barcode	12	-10697.60	-10633.49	5360.80	-10721.60	6.75	0.009
ASV-based sequence diversity	catchment_area:%cropland	Barcode	13	-10700.60	-10631.15	5363.30	-10726.60	5.00	0.025
Individually-reported species richness	%cropland	Primer	12	-1565.88	-1497.10	794.94	-1589.88	3.00	0.083
Individually-reported species richness	catchment_area:%cropland	Primer	13	-1566.38	-1491.88	796.19	-1592.38	2.51	0.113
Individually-reported func. redundancy	%cropland	Primer	12	-2814.60	-2747.15	1419.30	-2838.60	46.16	<0.001
Individually-reported func. redundancy	catchment_area:%cropland	Primer	13	-2812.61	-2739.54	1419.30	-2838.61	0.00	0.945
Tree-based MNTD	%cropland	Barcode	12	-1560.20	-1497.99	792.10	-1584.20	4.64	0.031
Tree-based MNTD	catchment_area:%cropland	Barcode	13	-1558.20	-1490.81	792.10	-1584.20	0.00	0.945
Barcode-derived sequence diversity	%cropland	Barcode	12	-879.98	-816.68	451.99	-903.98	32.00	<0.001
Barcode-derived sequence diversity	catchment_area:%cropland	Barcode	13	-879.44	-810.86	452.72	-905.44	1.46	0.227

We now clarified how human activity proxies were chosen and the model selection process and updated the results in the revised manuscript.

1) "Methods" section (Lines 661 to 680): "*We considered multiple available human-related variables, including human population density, percentage cropland cover (% cropland), and the Global Human Modification (gHM) index, capturing various components of human activity. Nighttime light data was used as a typical proxy for urbanization, and we directly used the nighttime light layer from Venter et al., 2016[75,76] developed based on the Defense Meteorological Satellite Program Operational Linescan System (DMSP-OLS) nighttime light data. Human population density was mapped using the Gridded Population of the World dataset developed by the Centre for International Earth Science Information Network[76,77]. Cropland cover was extracted*

from the 300-meter resolution land cover map developed by the European Space Agency Climate Change Initiative (ESACCI)[78]. The gHM index was derived from Kennedy et al. 201879, which quantified the degree of land modification by modeling the physical extents of 13 different anthropogenic stressors. To better represent the human activities from the surrounding upstream area, we extracted the above variables within a 4 km upstream flow distance buffer for each sampling site and the calculated the median as the estimates. Human population density and gHM were found to be highly correlated with nighttime lights (Supplementary Fig. 5, correlation coefficients of 0.61 and 0.73, respectively), while cropland cover showed weaker associations (correlation coefficient of 0.13). Based on these correlations, we ultimately selected two variables as human activity proxies: nighttime light as an urbanization proxy and cropland cover as an agricultural proxy." Lines 713 to 726: "*The full model further incorporated climate- and human activity–related variables, including their interactions with catchment area. Specifically, we included mean annual temperature as a proxy for climate, while nighttime light intensity and cropland cover as proxies for human activity, and tested whether spatial scaling relationships vary along climate or human activity gradients using interactions with catchment area. Nested models were compared using likelihood ratio tests to evaluate whether the inclusion of additional terms significantly improved model fit (Supplementary Table 5)83. Based on the results, we excluded the interaction between catchment area and cropland cover from the final model because likelihood ratio tests showed it was generally non-significant for the biodiversity metrics and its inclusion could lead to overparameterization (Extended Data Table 2). Therefore, the final model retained mean annual temperature, nighttime light intensity, and cropland cover, as well as the interactions between catchment area and mean annual temperature and between catchment area and nighttime light intensity.*"

2) "Results" section in Lines 259 to 406: we updated the modeling results accordingly.

3) "Discussion" section (Lines 473 to 481): "*Further, human activities generally weaken the species–area relationship, which is consistent with previous studies suggesting that human impacts reduce the likelihood of detecting rare, vulnerable, or habitat-specialist species, thereby flattening species – area relationship slopes24,58. In our case, this weakening was primarily associated with urbanization, whereas agriculture showed no significant effect. This generally aligns with recent reports of opposite effects of agricultural and urban land use on riverine fish assemblages, highlighting that the direction and strength of land-use impacts may be context dependent[59].*"

Comment #5: Line 166 – Genetic diversity mentions adaptive potential but the markers generally used in metabarcoding studies have been chosen for their neutral mode of evolution and not adaptative potential and so this needs to be clear that they are using the mb data as a proxy for broader genetic capacity.
RESPONSE: Thank you for pointing this out. We here refer largely to protein-coding markers (e.g., the COI gene), which are known to be under selection. Yet, we acknowledge that there is also some more neutral variability, particularly with respect to the third nucleic basis per protein codon. For our analysis, as they are indeed not strictly speaking population genetics analyses that depend on neutral markers, we now reformulate more cautiously. We have revised the text to avoid overstating "adaptive potential" and to clarify that we use marker-based genetic diversity as a proxy for broader standing genetic variation that can be related to population resilience.

The revised text now reads (Lines 166–169): "*Finally, genetic diversity is often associated with the capacity of populations to respond to environmental change, which can reduce population fluctuations and extinction risk, ultimately enhancing the long-term functional stability of ecosystems*[5,28]." We also revised the description of what eDNA can capture (Lines 178–180) to read: "*... calculation of phylogenetic diversity and, to some extent, assessment of genetic diversity from sequence variation at commonly used metabarcoding markers, which can serve as a proxy for broader standing genetic variation*[35,36]."

Comment #6: Line 244 – Can the team provide ecological information regarding the increase in MNTD at higher latitudes, which taxa are driving the results?
RESPONSE: We thank you for this suggestion. Please also see our response to *your **Comment #4*** (above) and also our response to ***Reviewer #3 Comment #6***. We have conducted additional analyses to better understand the driving lineages of the observed MNTD patterns. Specifically, we assessed changes in site-level MNTD following the sequential removal of individual family, allowing us to identify those taxa that contribute most strongly to the observed shifts in phylogenetic structure. Across all 115 families, the correlation coefficient (Pearson's r) between baseline MNTD (no family removed) and drop-one-family MNTD ranged from 0.95 to 1.00, and we observed no meaningful changes in MNTD estimates attributable to the removal of any single family. Together, these results suggest that the phylogenetic signal is more likely to reflect lineage turnover distributed across many parts of the tree, rather than being driven by a small set of ecologically distinctive lineages.

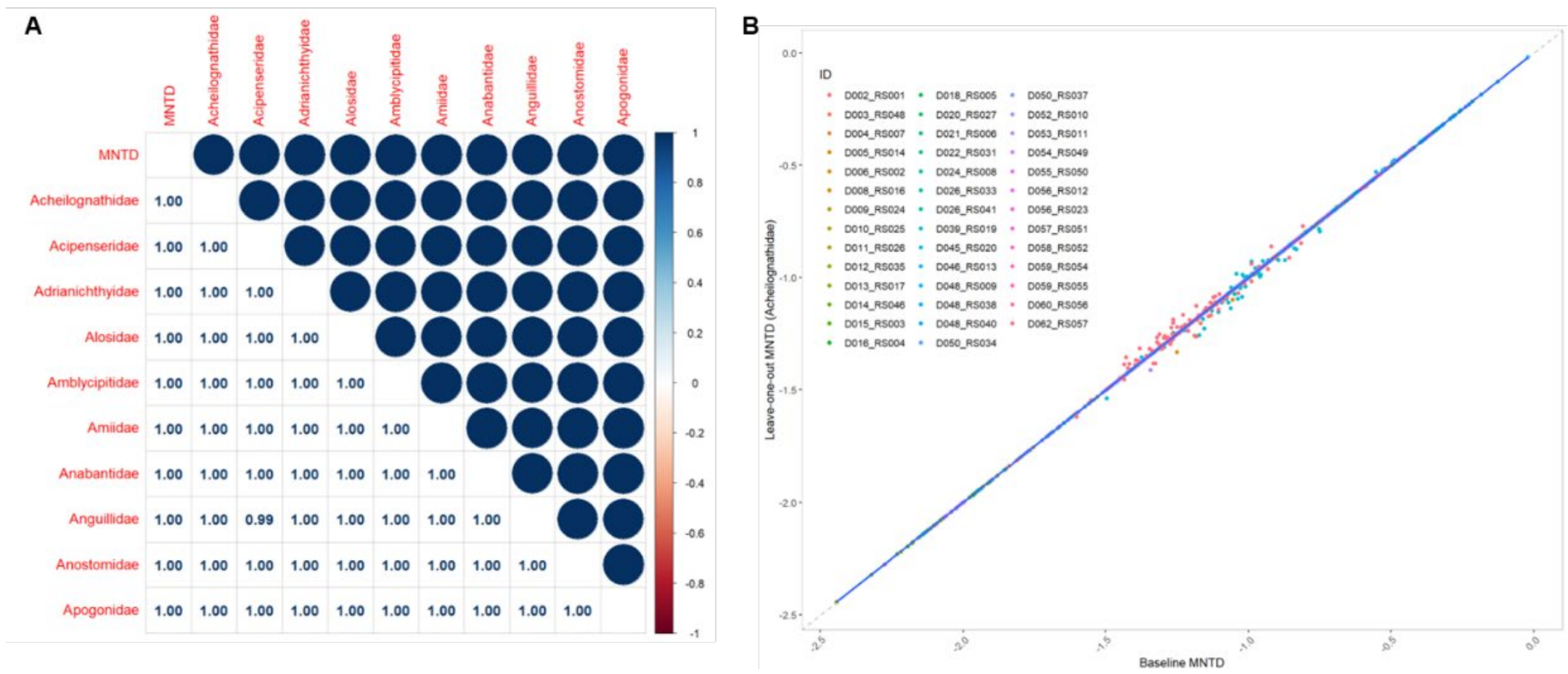

Correlation plot of drop-one-family MNTD, where "MNTD" denotes the baseline value with no family removed; only ten families are shown. Across all 115 families, the correlation coefficient (r) between baseline and drop-one-family MNTD ranges from 0.95 to 1.00.

Comment #7: Line 236 – Markers are mentioned here perhaps for the first time in the methods – we need more information earlier about markers, loci, primers etc..

RESPONSE: In the revised version, we now added a concise dataset/marker overview at the beginning of the Results that explicitly states the markers and primer sets represented in the compiled fish eDNA datasets, and we expanded the corresponding description in the Methods.

Results section, Lines 218 to 221: "*Because the compiled fish eDNA datasets differed in primer choice and sampling volume, we evaluated the robustness of downstream inferences by including primer/barcode and sampling volume as terms in the statistical models, as described in the Methods.*"

Method section, Lines 527 to 534: "*Given between-study variation in marker choice and laboratory design, we compiled record-level methodological descriptors, including primer set, barcode region, reported sample volume, and technical replication. The 58 datasets collectively employed 23 primer sets spanning four barcode regions (12S, 16S, COI, and CytB), and 12S assays such as MiFish and Teleo/Tele02 were used most frequently. Reported sample volumes ranged from 0.05 to 400 liters, and technical replication ranged from 1 to 36 (Supplementary Table 2).*"

Comment #8: Line 345 – There is a good example of space and biodiversity in Dobson et al. 2022 - https://www.sciencedirect.com/science/article/pii/S0043135422007175 ; consider citing?

RESPONSE: Thank you for the suggestion. We have added this citation in Line 152 to support the idea of how “space” (habitat availability at catchment level) is linked to biodiversity outcomes, and we have included the full reference in the bibliography.

Comment #9: Line 359 – human activity – can they find more human activity metrics, population, habitat modification etc.?

RESPONSE: In addition to nighttime lights, we have integrated several additional human activity metrics, including human population density, percentage cropland cover (%cropland), and the Global Human Modification (gHM) index. Human population density and gHM were highly correlated with nighttime light intensity (correlation coefficients of 0.61 and 0.73, respectively), whereas %cropland showed a weaker association (correlation coefficient of 0.13). We therefore retained nighttime light intensity as an urbanization proxy and %cropland as an agricultural proxy in the final model. Please refer to ***Comment #4*** for a detailed description of the variables, data sources, and model refitting and selection steps.

Comment #10: Line 411 – what does this look like on the ground? There is a temptation to suggest that some of the results/discussion are too superficial and not doing the rich data the justice that it deserves?

RESPONSE: We are not totally sure if we understand this comment correctly, but interpreted it as adding additional data on the spatial layout (we unfortunately cannot do additional field work on the ground itself). We have revised to clarify what the network-driven spatial structure means in practice (i.e., how expected biodiversity scaling and vulnerability differ between large/mainstem catchments and smaller/headwater catchments). The revised text now explicitly links our findings to stratified conservation actions and the use of the unified eDNA framework for priority-setting and intervention tracking. If we mis-interpret your comment, we are happy to get specific suggestions on how to revise the text.

Changes made in Line 425 to 429: “*These findings show that river-network spatial structure governs biodiversity scaling yet also predicts where human activity impacts are strongest. Practically, it helps to derive how conservation actions and eDNA-based monitoring should be tailored to catchment size and network position, with priorities differing between large catchments and smaller tributaries/headwaters.*”

Comment #11: Line 497 – what sort of compute power was used here please?

RESPONSE: The standardized processing of raw sequencing data was performed on a local workstation running Ubuntu 18.04 with 48 CPU cores and 125 GB RAM, without

GPU acceleration. We now added this information in the Supplementary Methods.

Comment #12: Line 549 – what markers were used. Here and elsewhere, what primers, markers were used?

RESPONSE: We now moved the information on markers used (previously in supplementary table 2) to the main manuscript at the beginning of the Results and expanded the corresponding description in the Methods.

Results section, Lines 218 to 221: "*Because the compiled fish eDNA datasets differed in primer choice and sampling volume, we evaluated the robustness of downstream inferences by including primer/barcode and sampling volume as terms in the statistical models, as described in the Methods.*"

Method section, Lines 527 to 534: "*Given between-study variation in marker choice and laboratory design, we compiled record-level methodological descriptors, including primer set, barcode region, reported sample volume, and technical replication. The 58 datasets collectively employed 23 primer sets spanning four barcode regions (12S, 16S, COI, and CytB), and 12S assays such as MiFish and Teleo/Tele02 were used most frequently. Reported sample volumes ranged from 0.05 to 400 liters, and technical replication ranged from 1 to 36 (Supplementary Table 2).*"

Comment #13: Line 602 – mean annual temperature – is this a good measure? Could high and low temps be more informative?

RESPONSE: Thank you for this comment. We initially explored a broader set of temperature predictors, including isothermality, temperature seasonality, max and min temperatures. In our dataset these temperature metrics were moderately to strongly correlated with each other and with mean annual temperature ($|r| > 0.6$). We therefore used mean annual temperature as a parsimonious and widely used summary of the thermal energy environment that is consistently available across all catchments. We now clarify this rationale in the Methods (Lines 654–659): "*We evaluated multiple BIOCLIM temperature variables representing temperature extremes and seasonality. Because these predictors were moderately to strongly correlated with one another and with mean annual temperature (Supplementary Fig. 5, $|r| > 0.6$), we used mean annual temperature as a parsimonious and widely used summary temperature metric to reduce collinearity and maintain interpretability.*"

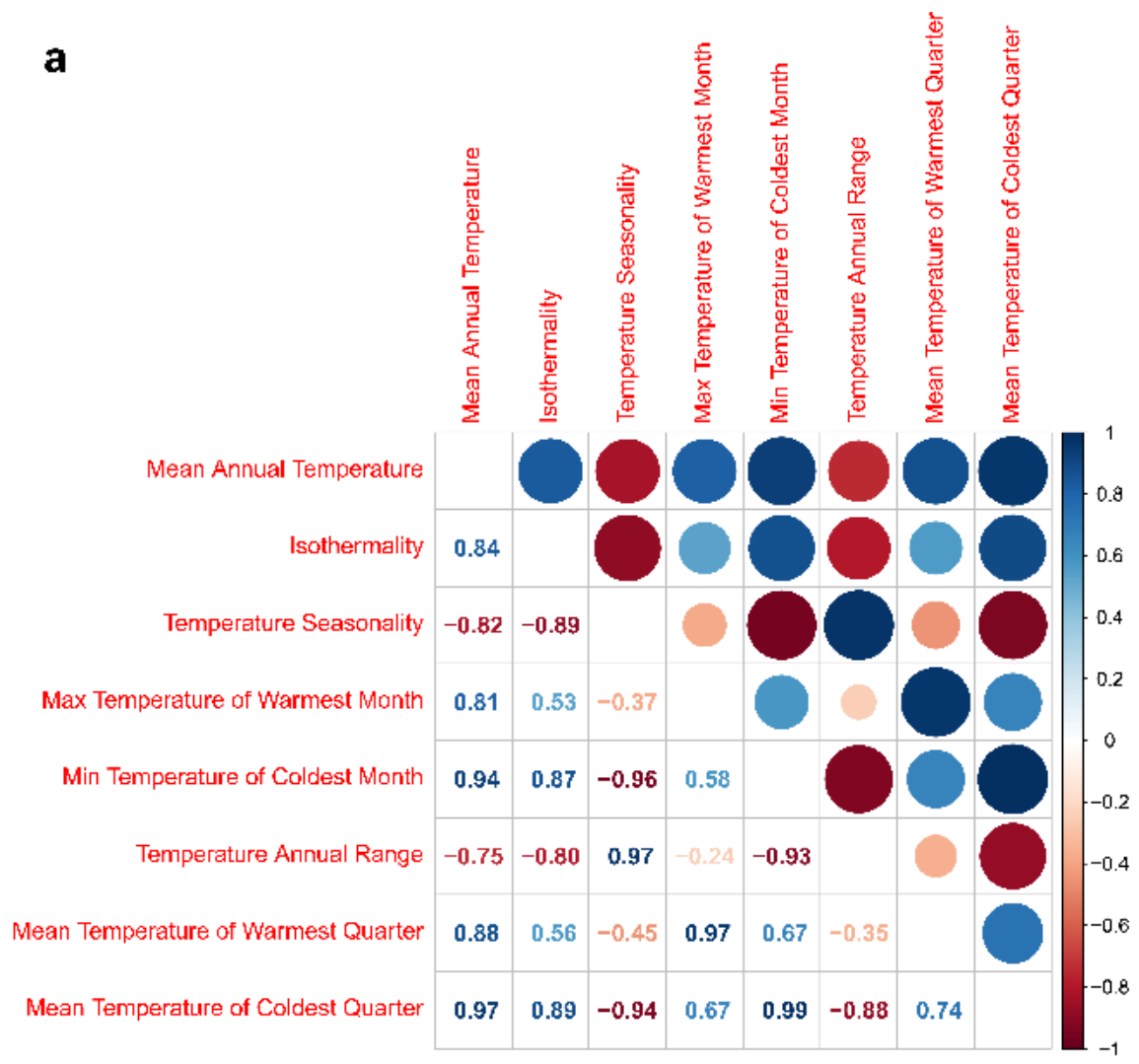

Supplementary Figure 5a Correlation plots of climatic factors.

Comment #14: Line 622 – Reference missing here?

RESPONSE: Thank you for noting this. We have added the missing reference at Line 692 in the revised manuscript and updated the reference list accordingly.

Comment #15: Figure 2 – d, e and f show S, MNTD and sequence diversity, but what about functional redundancy? What is going on with the vs. text with lightning bolts emerging from the text?

RESPONSE: In our study, functional redundancy is derived by combining species occurrence with a global trait database that largely provides species level summary traits. Because comparable data on within species trait variation across regions are not consistently available at the global scale, functional redundancy estimates cannot be independently benchmarked against a legacy reference in the same way as species richness, phylogenetic MNTD, or barcode-based sequence diversity. In addition, given the use of species level traits, functional redundancy is expected to co-vary with species composition and richness, which limits the value of presenting it as a separate validation target in Figure 2.

Regarding the symbols in Fig. 2, we revised the figure and caption to remove ambiguity. “VS” denotes the comparison between eDNA-derived and reference-based biodiversity estimates (Lines 1063 to 1065).

Comment #16: Line 944 – Is the very large spread on figures e and f, MNTD and sequence diversity derived from the rough proxy of all available sequences >600 bp for the comparision with existing data, ie not geolocated – could they be comparing unrelated things here and is enough mentioned in the text to acknowledge the potential limitations? How many sequences were included per species?

RESPONSE: We clarify that panel e (MNTD) is not based on COI barcode records, rather, MNTD was calculated from phylogenetic relationships derived from the unified eDNA data, and the reference comparison uses a previously published global freshwater fish phylogeny for the corresponding species list (via *FishPhyloMaker*). Therefore, the large scatter in panel e does not arise from using non-geolocated COI sequences but rather reflects differences between an amplicon-based phylogeny inferred from metabarcoding markers (predominantly 12S/16S ASVs) and a species-level global phylogeny, as well as differences in taxonomic resolution across datasets. We have revised the text and figure caption to make this distinction explicit.

For panel f (genetic sequence diversity), barcode-derived nucleotide diversity was calculated from all available deduplicated COI sequences (>600 bp) retrieved from NCBI for each species detected in the unified dataset. Indeed, these public COI records are not necessarily geographically matched to the sampled catchments (which they were in an ideal world), and that sampling effort varies among species. As stated in the Methods in Lines 601 to 605: "*We use for each species all available COI data as a first approximation of within-species genetic diversity, acknowledging this does not control for different sampling efforts between species or for the geographic distribution of barcode sequences, thus may not completely reflect a species' local genetic diversity, but is rather an integrated metric.*".

Further, we now report the number of COI sequences (downloaded from NCBI) included per species in Lines 598 to 601: "*Across 520 species, the number of qualifying COI sequences per species ranged from 1 to 215 (Supplementary Table 3, median = 8, IQR = 3–18). Nucleotide diversity was calculated for the 460 species with at least two sequences (range = 2–215, median = 9, IQR = 4–21).*"

Reviewer #2 (Remarks to the Author):

Comment #1: Overall, the manuscript is clearly written and the analytical framework is generally sound. The conclusions are well aligned with the results and are presented with appropriate nuance. While I was not able to examine every aspect of the manuscript in exhaustive detail, I found the study to be well structured and thoughtfully interpreted. I do, however, highlight two methodological points that would benefit from clarification or further justification:

RESPONSE: We sincerely appreciate your careful review of our manuscript and your constructive comments. We have revised the manuscript accordingly and clarified the aspects you pointed out. Please find the detailed point-by-point response as follows.

Comment #2: Genetic diversity estimates across markers:

The authors derive estimates of genetic diversity from different genetic regions (COI, 12S, and 16S). Because these loci evolve under distinct evolutionary models and rates, combining or comparing them requires an explicit evolutionary framework that accounts for marker-specific substitution processes. It is not clear from the current manuscript how these differences were handled, and additional explanation or justification would strengthen the robustness of the genetic diversity inferences.

RESPONSE: Thank you. Indeed, COI, 12S, and 16S are evolving under slightly different rates and substitution processes. Importantly, these markers had initially been used for their suitability for barcoding species (i.e., sufficient differentiation between species, relatively conserved within), and are this not directly comparable to time-calibrated multi-marker phylogenies. Also, the different primer pairs, even for the same markers, are amplifying minimally different amplicon lengths. We now added some text, contextualizing this better, and also adding sufficient caution that genetic diversity values are not time-calibrated (actually, this is very difficult to reach with such short amplicon length), and are thus to be understood as a first approximation of genetic diversity, yet keeping marker-specific effects in mind. We clarified our approach in the revised manuscript in Lines 446 to 458: "*Notably, our genetic diversity analyses specifically focus on within-species sequence variation, rather than between-species divergence. In eDNA analyses, it may not be feasible to use different unlinked markers for separate steps of species assignment versus within-species diversity estimation, because the physical linkage required to connect taxonomic assignment to a different locus is not available in environmental samples[54]. Therefore, the locus-specific nature and varying amplicon lengths of these markers should be considered when interpreting these within-species diversity proxies. In our dataset, the genetic sequence diversity estimates from the COI, 12S, and 16S barcodes are highly correlated with ASV-based genetic diversity, despite their different evolutionary rates and*

substitution processes[55]. These markers, chosen for their effectiveness in species barcoding[30,56], offer a first approximation of genetic diversity, though locus-specific selection pressures and varying amplicon lengths should be considered and caution generalization."

For the benchmark comparison, barcode-derived sequence diversity was calculated separately for COI, 12S, and 16S using NCBI sequences, and each locus-specific benchmark was compared to the corresponding ASV-based sequence diversity estimate (Fig. 2 and Extended Data Fig. 2). Importantly, these barcode-derived diversity estimates are strongly correlated across loci, indicating that the broad species-level patterns captured by the reference barcodes are consistent among markers and also reflecting the relatively conserved evolutionary models behind these makers (as sections of protein-coding genes, they are all expected to be under selection, thus cannot be seen as purely neutral genetic markers).

To account for marker/primer-specific differences in evolutionary rate and scale, we included Primer/Barcode as a random effect in our mixed-effects models. This explicitly controls for systematic differences among markers/primers while allowing inference on the shared ecological relationships. Importantly, we do not pool sequences across loci into a single estimate, and our conclusions rely on within-marker variation and on consistency across markers rather than on direct comparisons of absolute diversity values between loci.

Comment #3: Use of fishmorph traits:

The traits extracted from the FishMorph database are primarily morphometric shape descriptors. While these may carry potential functional implications, referring to overlaps or differences derived from these variables as "functional" may overstate their interpretability. I suggest clarifying the distinction between morphological and functional traits and moderating claims about functional differentiation unless supported by additional evidence.

RESPONSE: Thank you. Indeed, while morphology is often linked to ecological function and can provide informative proxies of functional strategies, we acknowledge that interpreting differences derived from these traits as strictly "functional" can be seen as a bit overstating their interpretability without additional functional evidence. Morphological traits were chosen because they provide more comprehensive and accessible information than other ecological, physiological, and behavioral traits. We have therefore revised the manuscript to make this distinction explicit and to moderate our wording in Lines 548 to 553: "*Morphological traits were chosen because*

they provide more comprehensive and accessible information than other ecological, physiological, and behavioral traits[61]. While they do not fully capture all functional aspects, they serve as indicators of certain functions62. Therefore, we clarify that these traits act as informative proxies for functional strategies, rather than direct functional evidence."

Comment #4: Overall, this is a valuable and thought-provoking study with considerable potential, and I encourage the authors to address the points above to further enhance clarity and rigor.

RESPONSE: Thank you for your very thorough and supportive evaluation! We truly appreciate your recognition and the feedback provided.

Reviewer #3 (Remarks to the Author):

Comment #1: This manuscript by Zhang et al. presents a globally harmonised synthesis of fish eDNA metabarcoding data from >1,800 sites across 113 river catchments worldwide. The authors reprocess publicly available raw sequence data to generate a unified ASV dataset and calculate taxonomic, functional, phylogenetic and genetic biodiversity metrics. They then model how these facets vary along river networks and interact with climate and human pressure. The work is ambitious, timely, and highly relevant to global biodiversity monitoring frameworks.

RESPONSE: We sincerely thank the Reviewer for the careful review and the very positive remarks. We greatly appreciate the recognition of the novelty and relevance of our work.

Comment #2: This is a good paper with significant conceptual and methodological contributions. The global integration of eDNA datasets is novel and valuable, and the multi-facet biodiversity framework is particularly compelling.

However, there are some areas where the manuscript could be improved, particularly regarding conceptual clarity, covariate interpretation, and the robustness of genetic diversity metrics, which require further refinement before publication. The manuscript would benefit from clearer articulation of mechanistic explanations and more detailed sensitivity analyses, especially regarding methodology biases and spatial sampling heterogeneity. The manuscript is innovative and promising, but could be strengthened by improving methodological robustness and clarifying mechanistic interpretations.

RESPONSE: We sincerely thank you for your careful review of our manuscript and for the constructive comments. We have revised the manuscript accordingly and clarified the aspects you highlighted. Please find our detailed point-by-point responses below.

Comment #3: The genetic diversity derived from short eDNA amplicons is interesting but methodologically limited. The authors acknowledge this, but more detail is needed on how much between- and within-species sequence variation is captured by short amplicon markers, and on the potential confounding effects of multiple species being grouped under imperfect taxonomic assignment. It would also be helpful to understand the geographic biases in COI sampling used for validation. I suggest including a sensitivity analysis illustrating how genetic diversity varies with (a) taxonomic assignment confidence, (b) read depth, and (c) marker type (12S vs 16S).

RESPONSE: We are happy to add more details on the use of the short eDNA amplicons as a proxy of genetic diversity. We agree that one would ideally have much longer DNA reads, yet this is simply not doable/available with the currently available eDNA data that are largely based on metabarcoding and (short) amplicon sequencing, mostly

using Illumina or analogue technologies. While other approaches (e.g., Oxford Nanopore long-range sequencing) are available, these have not yet been broadly applied. Thus, we simply have most of the data available falling into the category of these relatively short, barcode-based eDNA studies. For contextualization, we now firstly added a notion of caution, that the use of these short eDNA amplicons is the best we have, and how they still provide some insights on genetic diversity. Secondly, we also added additional sensitivity analyses as suggested.

Regarding the comment about how much between- and within-species sequence variation is captured by short amplicon markers, we want to clarify that our analysis specifically focused on within-species genetic variation, rather than between-species differences. Currently, it is difficult to find established benchmarks for comparing within-species genetic variation across different fish species. However, as shown in Figure 2f, the x-y axis demonstrates similar orders of magnitude between barcode-derived genetic diversity (10^{-3} to 10^{-1}) and ASV-based genetic diversity (10^{-3} to 10^{-1}), indicating comparable variation between these methods. We agree that such an analysis is based on a first split of amplicon variation between species (i.e., use reference databases to match amplicons to species, and then use these amplicons for within species diversity assessment). For eDNA analyses is physically not possible to use markers on different chromosome regions for these two different steps (see *Couton et al. (2023) Opportunities and inherent limits of using environmental DNA for population genetics. Environmental DNA 5: 1048–1064*), as the physical linkage required for first using one marker for species assignment and then another marker for within species evaluation is not given in eDNA. We now added an explanation on this in Lines 446 to 458: "*Notably, our genetic diversity analyses specifically focus on within-species sequence variation, rather than between-species divergence. In eDNA analyses, it may not be feasible to use different unlinked markers for separate steps of species assignment versus within-species diversity estimation, because the physical linkage required to connect taxonomic assignment to a different locus is not available in environmental samples[54]. Therefore, the locus-specific nature and varying amplicon lengths of these markers should be considered when interpreting these within-species diversity proxies. In our dataset, the genetic sequence diversity estimates from the COI, 12S, and 16S barcodes are highly correlated with ASV-based genetic diversity, despite their different evolutionary rates and substitution processes[55]. These markers, chosen for their effectiveness in species barcoding[30,56], offer a first approximation of genetic diversity, though locus-specific selection pressures and varying amplicon lengths should be considered and caution generalization.*"

Regarding taxonomic assignment, we employed a consistent bioinformatics pipeline for annotating ASVs. We acknowledge that taxonomic assignment may not be perfect (actually, it is very likely to not be perfect, for incomplete reference databases, errors in databases, and also taxa that do not have clearly delineated sequence variability at these markers used; the latter is for example well known for phylogenetically very young species such as known from rapid radiations, where COI is often not diversified). However, all of these points are true for any use of genetic reference databases. We ensured to be as conservative as possible and have minimized these effects by using local barcode reference databases which are correcting species assignments using local species lists for the specific river basin. That means, we only retained species with historical records within the basin, ensuring consistency across species in a community compared. We ensured this is clear in the main text (Lines 534 to 538): "*For datasets providing raw sequencing reads, we applied a standardized bioinformatic workflow to generate unified site-by-species matrixes comparable across studies. To reduce between-study annotation bias, we further constrained species assignments using basin-level fish historical occurrence records (see Supplementary Methods).*" And in the Supplementary Methods: "*To ensure accurate species annotation, we used historical species records to correct the species identification for each eDNA raw sequencing dataset. The historical species records were extracted from a global freshwater fish occurrence database encompassing 3,119 drainage basins worldwide [14]. This dataset compiles comprehensive lists of native, introduced, and invasive species. Each eDNA dataset was linked to its corresponding drainage basin and in cases without a direct match, the geographically nearest basin was used. Only species with documented historical records from the relevant drainage basin were retained for annotation. In this process, we avoided ASV rarefaction as sequencing depth varied significantly across studies.*"

As for the COI sampling and geographic biases, we note that the COI data in the reference database used for validation are not georeferenced and are not based on local references for each study site. Ideally, we of course would have had local georeferenced COI reference databases for every single study include, yet such data are largely lacking: only very few eDNA sets to date have complete, locally curated reference databases (see for example Blackman et al. 2023: General principles for assignment of communities from eDNA: open versus closed taxonomic databases. Environmental DNA 5: 326–342 which did such a study for Switzerland), and almost all studies are relying on regional databases. While we agree that such data would be perfect, the field of genetic barcoding and eDNA research is currently striving to have evenly distributed and (especially for taxa from understudied regions such as the

tropics) complete datasets with even few references per species and larger region (e.g., per continent). It is currently simply not possible to have locally curated reference data in such a meta-analysis, as this is not even available for most individual local studies (given that local reference databases are much better for eDNA amplicon alignment, individual studies would very much use such data if available, as they would be much easier to handle and interpret as more heterogeneous global reference databases). However, we now highlight this point stronger: having local and diversified, complete reference datasets would be the gold standard (see also Keck et al. 2023: Navigating the seven challenges of taxonomic reference databases in metabarcoding analyses. Molecular Ecology Resources 23: 742-755). We used all available COI data (from NCBI) for each species as a first approximation of within-species genetic diversity. While this approach does not control for sampling efforts across species, it provides an integrated metric for comparison. We have clarified that in the Method sections (Lines 601 to 605): "*We use for each species all available COI data as a first approximation of within-species genetic diversity, acknowledging this does not control for different sampling efforts between species or for the geographic distribution of barcode sequences, thus may not completely reflect a species' local genetic diversity, but is rather an integrated metric.*"

Regarding the suggested sensitivity analyses of genetic diversity with respect to (a) taxonomic assignment confidence, (b) read depth, and (c) marker type, we note that—as explained above and in our response to your ***Comment #12***—our taxonomic annotations were conservatively restricted to fish species with historical records in the respective basin, and thus did not consider explicit confidence scores for taxonomic assignments nor read depth. As a result, the only sensitivity analysis that can be implemented is with respect to marker type. To address this, we split the dataset into samples generated using 12S primers for metabarcoding (Panel A) and samples generated using non-12S markers (16S, COI, and CytB; Panel B). We then repeated the comparison between barcode-derived genetic diversity and ASV-based genetic diversity within each subset. The results show that, in the subset of samples using 12S primers, barcode-derived and ASV-based genetic diversity are significantly and consistently correlated (GLM model: $R^2 = 0.04$, $p < 0.001$), supporting the robustness of our main conclusion for the marker most commonly used in eDNA metabarcoding. In contrast, the subset using non-12S primers shows a non-significant relationship (GLMM model with Barcode as random factor: marginal $R^2 < 0.01$, conditional $R^2 = 0.45$, $p = 0.155$), which is likely driven by limited statistical power due to the smaller sample size (n = 204) and greater heterogeneity among marker types (16S/COI/CytB).

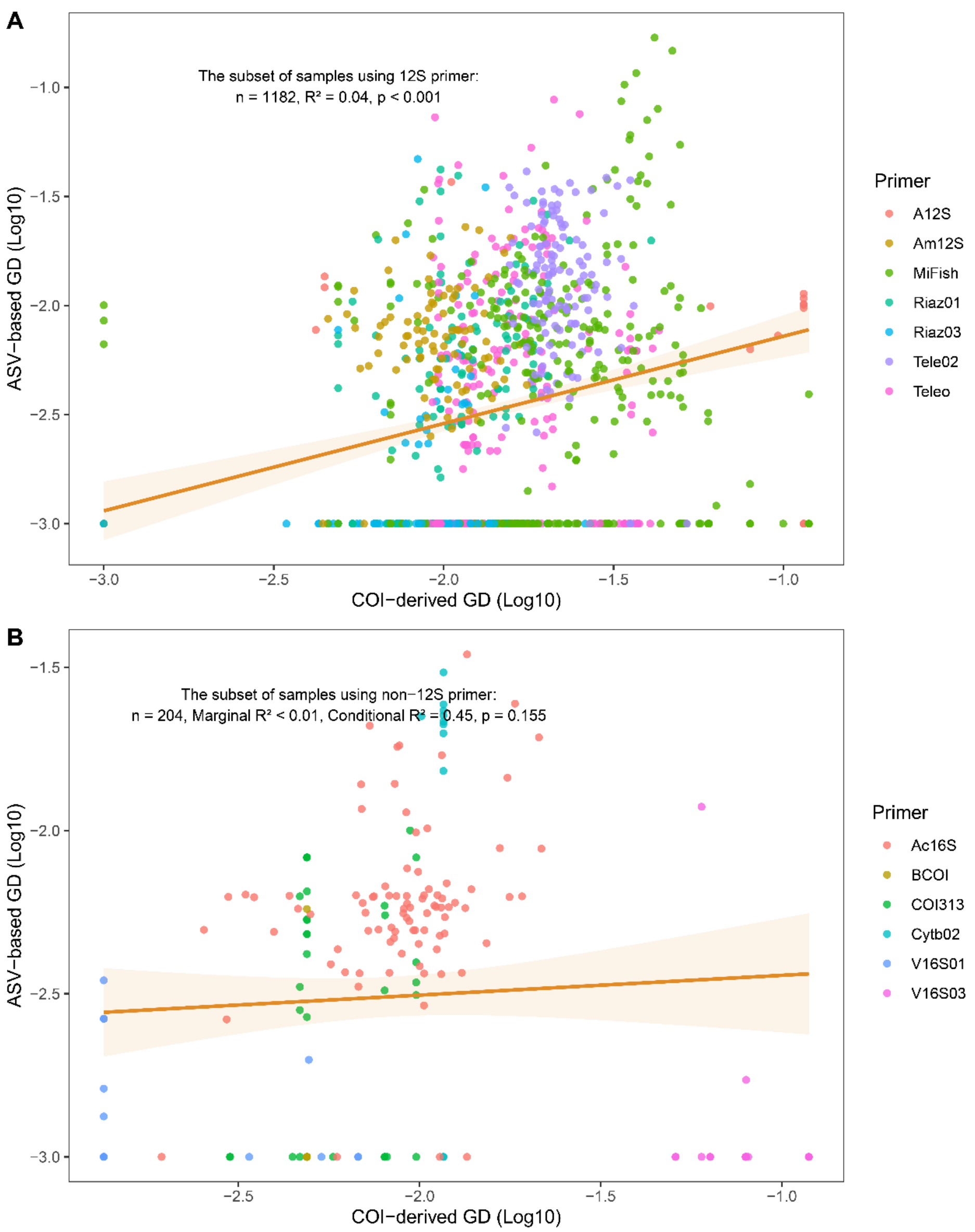

Comment #4: Although a unified ASV pipeline improves comparability, large differences remain across studies in sample volume, filtration, extraction, PCR cycles, primer pairs and sequencing platforms. Primer-specific random effects are helpful but may not capture systematic lineage-specific bias. It would be useful to provide a summary table quantifying the distribution of methodological variables across studies and, if possible, to add a supplementary model that explicitly tests sensitivity to filtration volume, primer set, lab of origin, and sequencing platform. This will help reassure readers that macroecological results are robust to protocol variation.

RESPONSE: Thank you, we completely agree! This large heterogeneity across eDNA metabarcoding studies is well-known, and efforts to be more homogeneous and standardized in approaches are underway (and much supported by us!). Yet, despite this heterogeneity, we still find strong and plausible patterns and interpretations with

respect to biodiversity, which underlines the robustness of our study. To accommodate for the heterogeneity, and plea for more standardized approaches in the future individual studies, we have made the summary table more visible and congruent. In the previous manuscript version, this information was already there yet mostly in the supplement. We now expanded and link it stronger to the main manuscript. Specifically, in the revised manuscript, we have strengthened our description of between-dataset protocol variation and clarified how our modeling framework accounts for such variation.

First, we now add an explicit description of the compiled record-level methodological metadata to the main manuscript in Lines 527 to 534 (previously, it was only given in the supplement): "*Given between-study variation in marker choice and laboratory design, we compiled record-level methodological descriptors, including primer set, barcode region, reported sample volume, and technical replication. The 58 datasets collectively employed 23 primer sets spanning four barcode regions (12S, 16S, COI, and CytB), and 12S assays such as MiFish and Teleo/Tele02 were used most frequently. Reported sample volumes ranged from 0.05 to 400 liters, and technical replication ranged from 1 to 36 (Supplementary Table 2).*"

Second, we revisited all original studies and ensured that all information highlighted by you is provided: we now add this important information on filtration volume, primer sets and sequencing platforms. Importantly, we note that quite many original studies rather sparsely reported on this information (thus, we sometimes cannot even extract it as it is lacking) and some of it is strongly confounded at the dataset level (e.g., primer choice often co-varies with platform). Including a large number of correlated protocol covariates simultaneously would substantially increase model complexity, consume degrees of freedom, and risk overfitting and unstable estimates given the effective sample size at the dataset level. For this reason, we prioritized a more parsimonious model structure (with primer/barcode region effects and sampling volume) and evaluated robustness through targeted alternative specifications and sensitivity analyses rather than fitting a single model with many explanatory variables.

We have clarified in the Methods (Lines 700 to 704) that protocol variation is already explicitly represented in our null-model specification. The null model accounts for amplification and sampling variation by including sampling volume as a covariate, a spatial term capturing broad-scale spatial autocorrelation as a fixed effect, and a random intercept for either primer or barcode region to absorb systematic amplification heterogeneity across datasets: "*The null model accounted for*

amplification and sampling variation by including sampling volume as a covariate, spatial autocorrelation as a fixed effect, and a random intercept for either primer or barcode region. Spatial autocorrelation was defined as the average minimum distance between sampling sites within each river catchment, reflecting local clustering while accounting for heterogeneity in catchment extent.".

Comment #5: Night-time lights correlate with urbanisation but not with other stressors such as agriculture, hydropower, nutrient or chemical pollution, or water flows or abstraction. Many catchments in the dataset are likely influenced by impacts not described by artificial lights. It may be worth adding at least one additional proxy (e.g. % cropland, population density, environmental flow modification index). If this is not possible, provide a stronger justification for relying solely on night-time light.

RESPONSE: Thank you, and this is a good suggestion (also in line with a suggestion by reviewer 1). We agree that night-time is primarily reflecting human settlement intensity/urbanization. To also reflect other dimensions of anthropogenic activities, we have now considered several further available human-related variables, including human population density, percentage cropland cover (%cropland), and the Global Human Modification (gHM) index, capturing different components of anthropogenic activities, including both urbanization and agricultural land-use changes. We find that population density and gHM are highly correlated with nighttime lights (with correlation coefficients of 0.61 and 0.73, respectively), whereas %cropland showed weaker associations (correlation coefficient of 0.13). Thus, also to avoid overparametrization, we decided to keep two human-related variables: the originally used nighttime lights as an urbanization proxy and the newly added %cropland as an agricultural proxy.

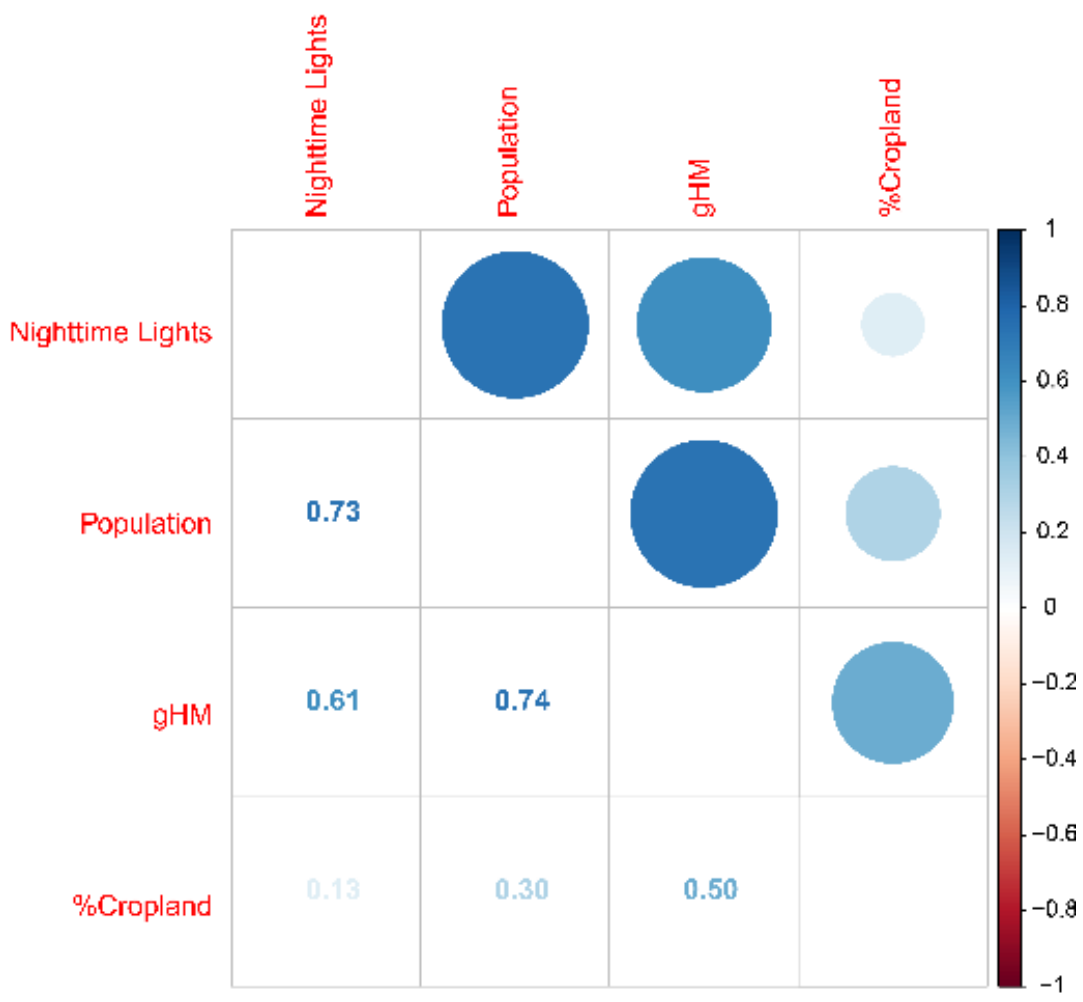

Supplementary Figure 5b Correlation plots of human activity factors.

We then re-fitted the model including both nighttime lights and %cropland as human activity proxies and used likelihood ratio tests to evaluate whether the main effects and interaction effects (with catchment area) of %cropland were significant across the eight biodiversity facets. The results showed that the main effect of %cropland was significant for all biodiversity metrics except individually reported species richness, whereas the interaction with cropland cover was generally non-significant (except for ASV-based sequence diversity and unified functional redundancy; see Extended Data Table 2). In the updated model, the results are highly robust relative to previous findings. The inclusion of this agricultural proxy supports the relevance of agriculture, alongside urbanization, as an anthropogenic driver. Importantly, the results were highly congruent both qualitatively and quantitatively with our previous analyses, corroborating the robustness of our approach. See also further explanations to response #4 to reviewer 1.

Extended Data Table 2 (partial) Results of likelihood ratio tests assessing the significance of individual model terms.

Biodiversity metrics	Test term	Random ter	df	AIC	BIC	logLik	deviance	Chisq	p value
Unified species richness	%cropland	Primer	12	-2797.03	-2732.33	1410.52	-2821.03	29.71	<0.001
Unified species richness	catchment_area:%cropland	Primer	13	-2798.04	-2727.95	1412.02	-2824.04	3.01	0.083
Unified func. redundancy	%cropland	Primer	12	-2759.81	-2696.66	1391.91	-2783.81	9.14	0.002
Unified func. redundancy	catchment_area:%cropland	Primer	13	-2776.35	-2707.94	1401.17	-2802.35	18.53	<0.001
ASV-based MNTD	%cropland	Barcode	12	-1242.94	-1180.26	633.47	-1266.94	19.79	<0.001
ASV-based MNTD	catchment_area:%cropland	Barcode	13	-1241.15	-1173.25	633.58	-1267.15	0.22	0.642
ASV-based sequence diversity	%cropland	Barcode	12	-10697.60	-10633.49	5360.80	-10721.60	6.75	0.009
ASV-based sequence diversity	catchment_area:%cropland	Barcode	13	-10700.60	-10631.15	5363.30	-10726.60	5.00	0.025
Individually-reported species richness	%cropland	Primer	12	-1565.88	-1497.10	794.94	-1589.88	3.00	0.083
Individually-reported species richness	catchment_area:%cropland	Primer	13	-1566.38	-1491.88	796.19	-1592.38	2.51	0.113
Individually-reported func. redundancy	%cropland	Primer	12	-2814.60	-2747.15	1419.30	-2838.60	46.16	<0.001
Individually-reported func. redundancy	catchment_area:%cropland	Primer	13	-2812.61	-2739.54	1419.30	-2838.61	0.00	0.945
Tree-based MNTD	%cropland	Barcode	12	-1560.20	-1497.99	792.10	-1584.20	4.64	0.031
Tree-based MNTD	catchment_area:%cropland	Barcode	13	-1558.20	-1490.81	792.10	-1584.20	0.00	0.945
Barcode-derived sequence diversity	%cropland	Barcode	12	-879.98	-816.68	451.99	-903.98	32.00	<0.001
Barcode-derived sequence diversity	catchment_area:%cropland	Barcode	13	-879.44	-810.86	452.72	-905.44	1.46	0.227

We now clarified how human activity proxies were chosen and the model selection process and updated the results in the revised manuscript.

1) "Methods" section (Lines 661 to 680): "*We considered multiple available human-related variables, including human population density, percentage cropland cover (% cropland), and the Global Human Modification (gHM) index, capturing various components of human activity. Nighttime light data was used as a typical proxy for urbanization, and we directly used the nighttime light layer from Venter et al., 2016[75,76] developed based on the Defense Meteorological Satellite Program Operational Linescan System (DMSP-OLS) nighttime light data. Human population density was mapped using the Gridded Population of the World dataset developed by the Centre for International Earth Science Information Network[76,77]. Cropland cover was extracted*

from the 300-meter resolution land cover map developed by the European Space Agency Climate Change Initiative (ESACCI)[78]. The gHM index was derived from Kennedy et al. 201879, which quantified the degree of land modification by modeling the physical extents of 13 different anthropogenic stressors. To better represent the human activities from the surrounding upstream area, we extracted the above variables within a 4 km upstream flow distance buffer for each sampling site and the calculated the median as the estimates. Human population density and gHM were found to be highly correlated with nighttime lights (Supplementary Fig. 5, correlation coefficients of 0.61 and 0.73, respectively), while cropland cover showed weaker associations (correlation coefficient of 0.13). Based on these correlations, we ultimately selected two variables as human activity proxies: nighttime light as an urbanization proxy and cropland cover as an agricultural proxy." Lines 713 to 726: "*The full model further incorporated climate- and human activity–related variables, including their interactions with catchment area. Specifically, we included mean annual temperature as a proxy for climate, while nighttime light intensity and cropland cover as proxies for human activity, and tested whether spatial scaling relationships vary along climate or human activity gradients using interactions with catchment area. Nested models were compared using likelihood ratio tests to evaluate whether the inclusion of additional terms significantly improved model fit (Supplementary Table 5)83. Based on the results, we excluded the interaction between catchment area and cropland cover from the final model because likelihood ratio tests showed it was generally non-significant for the biodiversity metrics and its inclusion could lead to overparameterization (Extended Data Table 2). Therefore, the final model retained mean annual temperature, nighttime light intensity, and cropland cover, as well as the interactions between catchment area and mean annual temperature and between catchment area and nighttime light intensity.*"

2) "Results" section in Lines 259 to 406: we updated the modeling results accordingly.

3) "Discussion" section (Lines 473 to 481): "*Further, human activities generally weaken the species–area relationship, which is consistent with previous studies suggesting that human impacts reduce the likelihood of detecting rare, vulnerable, or habitat-specialist species, thereby flattening species – area relationship slopes24,58. In our case, this weakening was primarily associated with urbanization, whereas agriculture showed no significant effect. This generally aligns with recent reports of opposite effects of agricultural and urban land use on riverine fish assemblages, highlighting that the direction and strength of land-use impacts may be context dependent[59].*"

Comment #6: The key finding is that richness, functional redundancy, and genetic diversity respond differently from phylogenetic diversity. However, the mechanisms are discussed mostly qualitatively. Consider expanding the discussion to clarify why phylogenetic diversity shows opposite area–human activity interactions, which specific lineages drive changes in MNTD, and whether homogenisation signals reflect loss of ancient lineages or proliferation of recent, widespread taxa.

RESPONSE: We thank the reviewer for this suggestion. We were cautious in our interpretations and – for the comparative nature of our study – had largely focused on qualitative interpretations (also to avoid overinterpretations and implying mechanistic links). However, given your suggestion (and also in agreement with comments #4 and #6 by ***Reviewer 1***), we have conducted additional analyses to better understand the driving lineages of the observed MNTD patterns. Specifically, we assessed changes in site-level MNTD following the sequential removal of individual family, allowing us to identify those taxa that contribute most strongly to the observed shifts in phylogenetic structure. Across all 115 families, the correlation coefficient (Pearson's r) between baseline MNTD (no family removed) and drop-one-family MNTD ranged from 0.95 to 1.00, and we observed no meaningful changes in MNTD estimates attributable to the removal of any single family. Together, these results suggest that the phylogenetic signal is more likely to reflect lineage turnover distributed across many parts of the tree, rather than being driven by a small set of ecologically distinctive lineages.

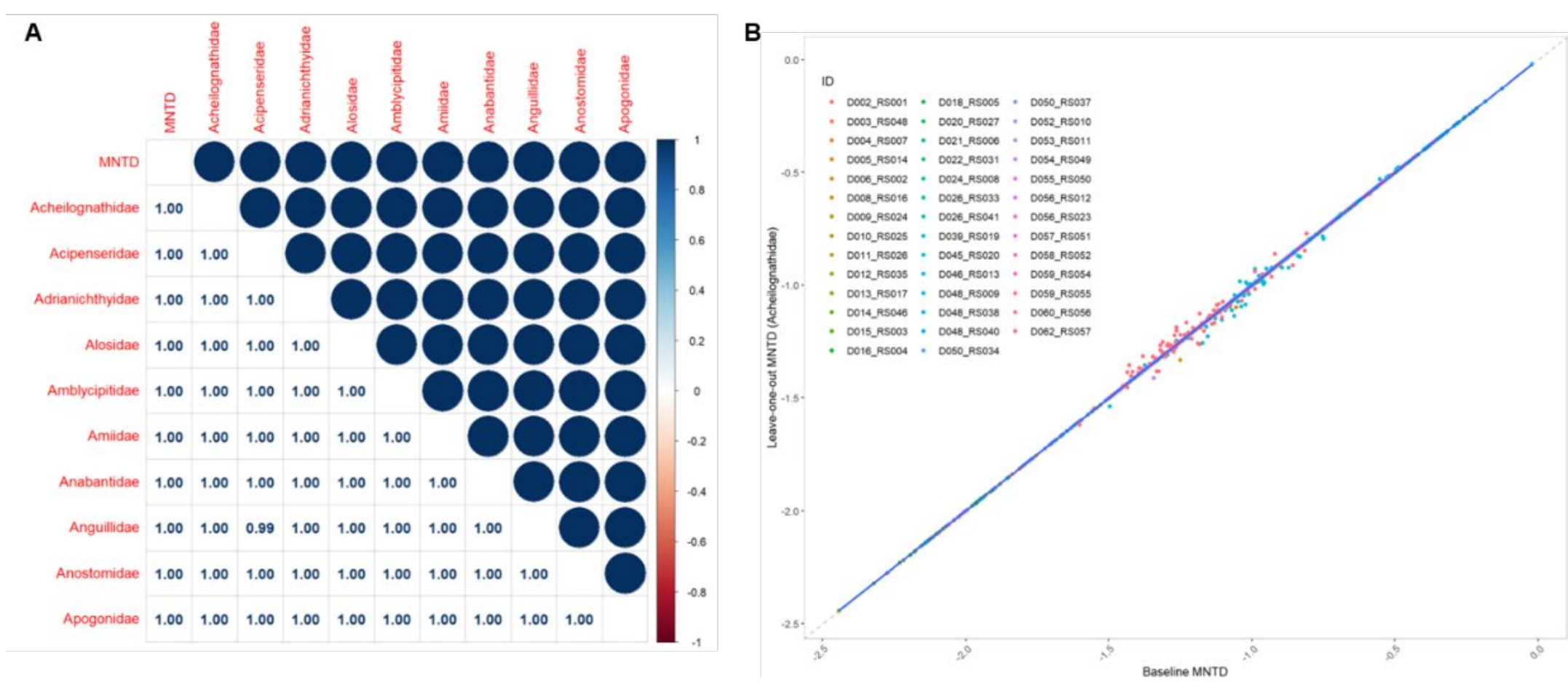

Correlation plot of drop-one-family MNTD, where "MNTD" denotes the baseline value with no family removed; only ten families are shown. Across all 115 families, the correlation coefficient (r) between baseline and drop-one-family MNTD ranges from 0.95 to 1.00.

Comment #7: Clarify whether COI barcode sequences from GenBank were filtered for quality and geographic relevance.

RESPONSE: Thank you for this comment. We confirm that the COI barcode sequences

downloaded from GenBank/NCBI were filtered for basic quality and comparability. Specifically, we retained only deduplicated sequences longer than 600 bp (following Manel et al.) and removed obvious duplicates prior to calculating within-species nucleotide diversity. Unfortunately, we could not filter sequences by geographic relevance because location metadata are incomplete and inconsistent across many NCBI records for the species considered. Accordingly, our COI-based π_s should be interpreted as a species-level, globally aggregated approximation of within-species sequence diversity rather than a direct estimate of local population genetic diversity. We have clarified this limitation more explicitly in lines 596 to 605: "*COI was selected for its high resolution in species discrimination. Only deduplicated sequences longer than 600 bp were retained, and π_s was calculated as described above. Across 520 species, the number of qualifying COI sequences per species ranged from 1 to 215 (Supplementary Table 3, median = 8, IQR = 3–18). Nucleotide diversity was calculated for the 460 species with at least two sequences (range = 2–215, median = 9, IQR = 4–21). We use for each species all available COI data as a first approximation of within-species genetic diversity, acknowledging this does not control for different sampling efforts between species or for the geographic distribution of barcode sequences, thus may not completely reflect a species' local genetic diversity, but is rather an integrated metric.*"

Comment #8: Consider discussing the potential influence of invasive fish species on patterns of functional and genetic diversity.

RESPONSE: Thank you for this suggestion. We indeed had this considered already during our main analysis, as it was also of interest to us. However, when aiming for it we realized quickly that invasion status is strongly context dependent (each non-native species is native someplace else, and non-native species can be either invasive or not, yet we largely lacked this information for the local datasets). As such, we then had decided against explicitly model invasive status in this global synthesis. Specifically, we had invested quite some time to get information on native/non-native status and invasiveness, yet the same species can be native in one basin and non-native in another, and harmonized basin-level information on invasion status and introduction history is not consistently available across the studied catchments included here (even on small scales: non-nativeness is often reported at country level, yet many species can be non-native within countries when crossing watersheds). Given the heterogeneous distribution of our data, a further discretization of our dataset along invasive species was not feasible (we really tried, as this was an obvious question! But had to give up on this idea as the dataset was not large enough/robust enough for such an analysis).

However, realizing the overall interest in invasive species, we now conducted a descriptive assessment of the potential overlap between our dataset and widely reported invasive taxa. We compiled the top ten invasive fish species for each ecoregion (data extracted from Bernery *et al.* 2022, 10.1146/annurev-ecolsys-032522-015551.hal-03781186v2) and quantified their occurrence across the catchments in our dataset. We did so, as these top invasive species are well known and unanimously reported as invasive (and often the focus of many studies too).

Yet, based on the resulting overlap analysis, 47.6% (49/103) datasets (with species-site table available; identified by the "ItemID" in Supplementary Table2) contained none of these top invasive species (see figure below). Among the remaining datasets, most contained only one species (29/103; 28.2%), indicating that top invasive species were generally rare across datasets (78/103; 75.7% with zero or one). This suggests that the broad functional and genetic diversity patterns reported here are unlikely to be driven by a small set of widely recognized invaders. The main reason for this rare occurrence of these invasive species in our datasets may be mostly due to the strong bias of eDNA studies to streams and small rivers: non-native species are predominantly found in large rivers, yet many eDNA studies are conducted in small headwater catchments. Despite our extensive meta-analysis, the eDNA data are (yet) still to sparse for such a global analysis on invasive species.

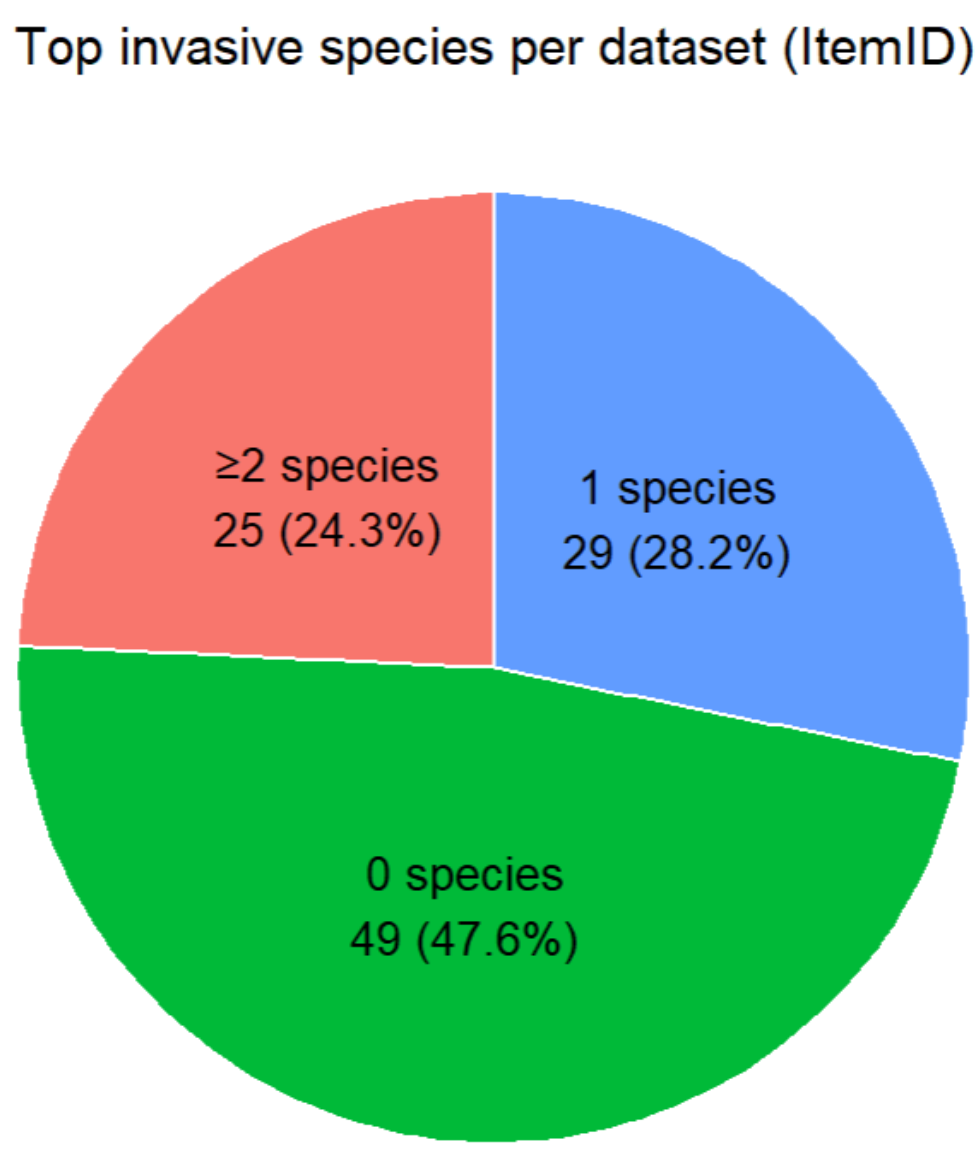

Comment #9: Provide effect sizes in addition to significance for all main models, preferably in a standardised format.

RESPONSE: Thank you for this comment and we completely agree. Actually, effect sizes

for all main models had already been provided in the previous manuscript version in a standardized format in Extended Data Table 2 reporting the standardized fixed-effect estimates for each biodiversity metric). We apologize if this was not clear/obvious, and we now clarified it and made it more standardized/obvious to the reader.

Comment #10: Provide details on how pseudo-replicated sites (multiple samples at the same coordinates) were handled.

RESPONSE: Thank you for raising this point. A few (19 out of 113) river catchments indeed included observations at identical or similar coordinates (Supplementary Table 2), arising from (i) multi-marker assays where the same eDNA sample was analyzed with multiple primer/barcode regions, and/or (ii) repeated sampling at the same location within a study (normally with different sampling years and/or seasons). We now provide this information. For the analysis, we did not pool these observations a priori because they represent either distinct marker-specific detection processes (case i) or repeated measurements intended to characterize the same site (case ii). Instead, we handled them within the modelling framework to account for non-independence and methodological heterogeneity. Specifically, in the null model we included sampling volume as a covariate, spatial autocorrelation as a fixed effect, and a random intercept for primer identity or barcode region (depending on the response metric). This structure accommodates multi-marker pseudo-replication by attributing systematic differences in detectability/amplification among markers to the Primer/Barcode random effect, rather than averaging across loci. We now clarified this in Lines 705 to 709: "*Notably, few (19 out of 113) river catchments reported multiple observations at identical or near-identical coordinates due to multi-marker assays and/or repeated sampling across seasons or years. Because this can create non-independence and overweight a site, we did not merge these a priori. Instead, we accounted for them within the null model.*"

In addition, to ensure that our inferences were not driven by the small number of pseudo-replicated coordinates (or by any single subset of sites), we evaluated robustness using our resampling-based validation framework: we repeatedly re-fit the full models under 1,000× downsampling (randomly retaining 70% of sites each iteration) and compared the resulting effect-size distributions to 1,000× covariate reshuffles. This procedure reduces sensitivity to any pseudo-replicated observations because no small group of co-located samples is guaranteed to be retained in every iteration, and it provides a conservative null benchmark for inference (Lines 754 to 761).

Comment #11: Expand the explanation for why species richness correlates more strongly with historical data in the unified dataset compared to the original datasets.

RESPONSE: In the unified analysis, we reanalyzed all datasets with available raw sequencing data using a single, standardized bioinformatics pipeline. This approach minimizes potential biases that could arise from differences in bioinformatics processing across the original studies. This may explain why the unified dataset showed a slightly higher marginal R^2 than the original isolated reported datasets (marginal R^2 0.16 versus 0.10). However, the improvement is modest and does not provide strong evidence for a systematic or substantially stronger relationship. Therefore, we revised the wording in the Results section to avoid overstating the unified correlation as "stronger." Instead, we clarified that both the original and unified richness estimates are positively correlated with historical species richness (Lines 241–243): "*Similarly, the correlations were also significant for the original individually reported species richness (Extended Data Fig. 1, GLMM, $p < 0.001$, marginal $R^2 = 0.08$–0.10, conditional $R^2 = 0.39$–0.68).*"

Comment #12: Clarify whether rarefaction was applied to reads before ASV calling.

RESPONSE: We did not apply rarefaction to the reads before ASV calling, also as the use (and justification) of rarefaction in eDNA data is highly debated. Given that we include datasets spanning >10 years, were sequencing depth across studies increased massively, one would need to apply a between-study rarefaction, which would not reflect increasing sequencing depths and also waste a lot of data. In our unified analysis, we rather employed a consistent bioinformatics pipeline, particularly for annotating ASVs using a local species list. One important consideration is that using local barcode reference databases for taxonomy assignment can help minimize bias across these sporadic and isolated datasets. Additionally, sequencing depth varied between datasets, and for ASVs with low abundance, they may indeed represent true biological diversity. Overall, we think there is no objective approach to establish a universal, reasonable cutoff or sequencing depth that could filter ASVs consistently across different datasets. Therefore, we focused on whether ASVs could be assigned to species with documented historical records, rather than applying rarefaction. We have clarified this approach in the Supplementary Methods for better transparency: "*To ensure accurate species annotation, we used historical species records to correct the species identification for each eDNA raw sequencing dataset. The historical species records were extracted from a global freshwater fish occurrence database encompassing 3,119 drainage basins worldwide[14]. This dataset compiles comprehensive lists of native, introduced, and invasive species. Each eDNA dataset was linked to its corresponding drainage basin and in cases without a direct match, the*

geographically nearest basin was used. Only species with documented historical records from the relevant drainage basin were retained for annotation. In this process, we avoided ASV rarefaction as sequencing depth varied significantly across studies."

Again, many thanks for your very constructive and detailed comments that helped improve our study!
